# *Bis*-Iridoids: Occurrence, Chemophenetic Evaluation and Biological Activities—A Review

**DOI:** 10.3390/molecules29235646

**Published:** 2024-11-28

**Authors:** Claudio Frezza, Alessandro Venditti, Daniela De Vita, Marcella Guiso, Armandodoriano Bianco

**Affiliations:** 1 Dipartimento di Scienze della Vita, della Salute e delle Professioni Sanitarie, Università degli Studi Link Campus, 00165 Rome, Italy; 2 Independent Researcher, 00040 Rome, Italy; alessandro.venditti@gmail.com; 3 Dipartimento di Biologia Ambientale, Università degli Studi di Roma “La Sapienza”, 00185 Rome, Italy; daniela.devita@uniroma1.it; 4 Dipartimento di Chimica, Università degli Studi di Roma “La Sapienza”, 00185 Rome, Italy; marcella.guiso@fondazione.uniroma1.it (M.G.); armandodoriano.bianco@fondazione.uniroma1.it (A.B.)

**Keywords:** *bis*-iridoids, occurrence, chemophenetic value, biological activities

## Abstract

In this work, the first review paper about *bis*-iridoids was presented. In particular, their detailed occurrence, chemophenetic evaluation and biological activities were reported. To the best of our knowledge, two hundred and eighty-eight *bis*-iridoids have been evidenced so far, bearing different structural features, with the link between two *seco*-iridoids sub-units as the major one. Different types of base structures have been found, with catalpol, loganin, paederosidic acid, olesoide methyl ester, secoxyloganin and loganetin as the major ones. Even *bis*-irdioids with non-conventional structures like intra-cyclized and non-alkene six rings have been reported. Some of these compounds have been individuated as chemophenetic markers at different levels, such as cantleyoside, laciniatosides, sylvestrosides, GI-3, GI-5, oleonuezhenide, (*Z*)-aldosecologanin and centauroside. Only one hundred and fifty-nine *bis*-iridoids have been tested for their biological effects, including enzymatic, antioxidant, antimicrobial, antitumoral and anti-inflammatory. Sylvestroside I was the compound with the highest number of biological tests, whereas cantleyoside was the compound with the highest number of specific biological tests. *Bis*-iridoids have not always shown activity, and when active, their effectiveness values have been both higher and lower than the positive controls, if present. All these aspects have been deeply discussed in this paper, which also shows some critical issues and even suggests possible arguments for future research, since there is still a lot unknown about *bis*-iridoids.

## 1. Introduction

*Bis*-iridoids are a sub-class of iridoids characterized by the link of two iridoidic *sensu lato* sub-units to form a bigger molecule. Actually, these sub-units may be extremely different, and the bond may occur in different positions of both the sub-units, including the glucose moiety but also after conjugation with other classes of natural compounds like phenolics and terpenes to act as a bridge between them [1,2,3,4,5].

They are biosynthesized following the general route for the biosynthesis of simple iridoids and *seco*-iridoids but with the further passage of the intermolecular bond of the two sub-units alone or after conjugation with bridges [6].

In the literature, there is no specific review paper on *bis*-iridoids, whereas several review papers have dealt with the topic of iridoids in general on several aspects [1,2,3,4,5,7,8,9,10].

In this review paper, the occurrence, chemophenetic value and biological activities of *bis*-iridoids are presented and discussed in detail. The literature search was conducted on renowned scientific databases such as PubMed, PubChem, Google Scholar and Reaxys using keywords like *bis*-iridoid, *bis*-iridoids, occurrence, biological activities alone or together and specific names of compounds or plant species, as recovered from previous papers. All the papers written in English in spite of their publication year and journal were considered. Not fully accessible papers were also included. Indeed, all the papers not concerning plant species, concerning a mixture of plants where the identification of this type of compounds has not been clearly attributed, deriving from cell cultures or from sure enhancement of their production in a botanical or biotechnological manner, were neglected.

## 2. Occurrence of *Bis*-Iridoids in Plants

Table 1 reports on the occurrence of *bis*-iridoids in plants in alphabetical order. In this, the organs of the plants where they have been recovered and the collection area of the species, as well as the methodologies adopted for their extraction, separation and identification, are also presented.

To the best of our knowledge, two hundred and eighty-eight *bis*-iridoids have been identified in plants, so far. Sixty are structurally characterized by the link between two iridoid sub-units, fifty-four by the link between one iridoid sub-unit and one *seco*-iridoid sub-unit, ninety-two by the link between two *seco*-iridoid sub-units, nine by the link between two non-glucosidic iridoid sub-units, eleven by the link between one non-glucosidic iridoid sub-unit and one non-glucosidic *seco*-iridoid sub-unit, six by the link between one iridoid sub-unit and one non-glucosidic iridoid sub-unit, thirty-four by the link between one non-glucosidic iridoid sub-unit and one *seco*-iridoid sub-unit, twenty-two by a non-conventional *bis*-iridoid structure. By consequence, *bis*-iridoids with two *seco*-iridoid sub-units are the most abundant, whereas *bis*-iridoids with one iridoid sub-unit and one non-glucosidic iridoid sub-unit are the least abundant.

Different types of iridoid, *seco*-iridoid and non-glucosidic iridoid base structures are used to form *bis*-iridoids. Catalpol, loganic acid, loganin and paederosidic acid, together with their derivatives, are the most common for iridoids, whereas oleoside methyl ester and secoxyloganin, together with their derivatives, are the most common for *seco*-iridoids and loganetin, together with its derivatives, is the most common for non-glucosidic iridoids. Other present base structures for iridoids include 8-*O*-acetyl-harpagide, adoxoside, arborescoside, ajugoside, anthirride, anthirrinoside, aucubin, euphroside, gardenoside, gardoside, geniposide, scandoside and their derivatives. Other present base structures for *seco*-iridoids include morronoside, *seco*-loganol, *seco*-loganoside, swertiamarin, 9-oxo-swerimuslactone A and their derivatives. Other present base structures for non-glucosidic iridoids include *iso*-boonein, alyxialactone and their derivatives. Indeed, among the non-conventional bonds, there are intra-cyclic *bis*-iridoids, bonds with differently functionalized five carbon rings fused with other rings or not, and bonds with iridoids deprived of their classical double bond between carbons 3 and 4. From a specific observation of these base structures, it can be easily established that not all the existing base structures for iridoids, *seco*-iridoids and non-glucosidic iridoids are present in *bis*-iridoids, as well as not all the possible non-conventional bonds, and this may, indeed, represent an interesting research line for the future.

For what concerns the general structures of *bis*-iridoids, the literature survey has displayed some important issues. The first one regards the real existence of compounds having methyl, ethyl and dimethyl acetal groups, like in abelioside A methyl acetal, abeliforoside C, abeliforoside E, cantleyoside dimethyl acetal, cocculoside, dipsanoside J, saugmaygasoside D, sylvestroside III dimethyl acetal, sylvestroside IV dimethyl acetal, triplostoside A and tripterospermumcin B methyl acetal or having methyl ester, ethyl and butyl groups, like in aldosecolohanin B, atropurpurins A–B, pterocesides A–C, cornuside K, hookerinoid A, hookerinoid B, pterhookeroside and tricoloroside methyl ester. Given the methodologies adopted for their extraction and isolation, these compounds are likely to be artifacts [239], even if they are often found, thus evidencing their extreme ease of formation. Yet, these have not been considered as artifacts but as natural. It is not very simple to establish which is correct, but this whole situation can be easily solved by a simple analytical procedure constituted of steps of maceration, separation and identification using non-corresponding solvents, meaning not methanol for methyl acetal, dimethyl acetal and methyl ester compounds and not ethanol and butanol for ethylated and butylated compounds. The presence of these functional groups in the same compounds obtained following this way will be clear evidence of the fact they are not artifacts. In this sense, this topic may also be an involved line for future research. Another detected issue regards (*E*)-aldosecologanin and centauroside. Indeed, they are often considered as different compounds, but they present the same structure, and thus, they are the same compound. In the future, more attention must be paid to this aspect. Another issue is surely the need for major harmonization on the names of these compounds. This has been widely shown for the compounds named GI-3 and GI-5 in this paper. Actually, in others, they are named Gl-3 and Gl-5 or GL-3 and GL-5, but they are all the same. One single name for each compound is compulsory in order to avoid confusion and possible identification mistakes. Lastly, it is important to underline that most of the existing *bis*-iridoids have trivial names but not in a few cases: dimer of alpinoside and alpinoside, dimer of aperuloside and asperulosidic acid, dimer of nuezhenide and 11-methyl-oleoside, dimer of oleoside and 11-methyl-oleoside, dimer of paederosidic acids, dimer of paederosidic acid and paederoside, dimer of paederosidic acid and paederosidic acid methyl ester. The choice of giving trivial names to new compounds is always up to the authors, but this should always be encouraged, since it can really diminish the possibility of giving different names to the same structure, considering them to be new when they are not. The most fitting example of this is the compound named in this review as iridoid glycoside dimer I.

The most present compound in plants is cantleyoside, which has been reported in twenty-one different species belonging to ten different genera and four different families. Its highest occurrence is in four different genera (*Cephalaria*, *Dipsacus*, *Pterocephalus* and *Strychnos*), whereas, in two genera (*Abelia* and *Lomelosia*), its presence is singular. Conversely, several compounds have been found in single species. The presence of specific compounds in different species of the same genus, in different genera of the same family and in different families of the same order is extremely important, since it allows the individuation of chemophenetic markers at these levels. On the contrary, the presence of specific compounds in single species has no chemophenetic relevance due to their extremely limited distribution. The compound with the highest number of reports in the same species is centauroside in *Lonicera japonica* with twenty-three citations. Centauroside is also the compound with the highest number of studies for different populations of the same species (*Lonicera japonica*) collected in different countries. The multiple presence of the same compound at every classification level confirms that this compound is usually biosynthesized here, which is extremely important under the chemophenetic standpoint, potentially considering it as a chemophenetic marker.

For what concerns the organs of the species studied, flowers, flower buds, seeds, twigs, leaves, stems, stem bark, bark, wood, heartwood, roots and rhizomes have all been mentioned. A combination of two different organs has also been studied (stems and leaves, leaves and branches, flowers and twigs, bark and wood and roots and rhizomes), as well as more organs (whole plant, aerial parts, flowering aerial parts, foliage and underground parts). In some papers, the organs studied have been dried (generally, in the open air) prior to the phytochemical analysis, as dictated by the local Pharmacopeias (roots of *Dipsacus inermis*, flower buds and roots of *Lonicera* spp. and dried fruits of *Ligustrum* spp.). In all the other cases, the organs were fresh. For non-volatile secondary metabolites like *bis*-iridoids, the renowned issue regarding the utilization of dried or fresh organs for the phytochemical analysis is not so relevant given that they are generally stable at high temperatures but not too high [240,241].

For what concerns the collection areas of the species, all the continents are included. The highest number of reports where *bis*-iridoids have been found is in Asian countries, with China as the most numerous. The countries with the highest numbers of reports are Italy for Europe, Algeria for Africa, the USA for America and New Caledonia for Oceania. On the other hand, some countries (Montenegro, Namibia and Tanzania) have been mentioned only once. The number of reports for the occurrence of *bis*-iridoids in the plants of different territories is strictly correlated with the number of species in the territory that biosynthesize them, but it is not an absolute mirror of their worldwide distribution, since this also depends on their search. Either way, a little parallelism between the distribution of iridoids and *bis*-iridoids is present [242].

For what concerns the methodologies for the extraction, isolation and identification of *bis*-iridoids, classical procedures have been utilized. Maceration has been the most common extraction method. Column chromatography and HPLC techniques have been mostly employed as separation methodologies, whilst different spectroscopic and spectrometric techniques together have been used for the identification. All these methods are widely accepted for the analysis of non-volatile metabolites, not causing big issues, except for those previously discussed.

The structures of all the fully characterized *bis*-iridoids isolated from plants are reported in Figure 1, Figure 2, Figure 3, Figure 4, Figure 5, Figure 6, Figure 7, Figure 8, Figure 9, Figure 10, Figure 11, Figure 12, Figure 13, Figure 14, Figure 15, Figure 16, Figure 17, Figure 18, Figure 19, Figure 20, Figure 21, Figure 22, Figure 23, Figure 24, Figure 25, Figure 26, Figure 27, Figure 28, Figure 29, Figure 30, Figure 31, Figure 32, Figure 33, Figure 34 and Figure 35.

The dimer of alpinoside and alpinoside, the dimer of nuezhenide and 11-methyl-oleoside, the dimer of oleoside and 11-methyl-oleoside, demethyl-hydroxy-oleonuezhenide, demethyl-oleonuezhenide, hydroxy-oleonuezhenide and oleoneonuezhenide have not been fully characterized, and their structures have not been drawn. This may surely be an argument for future research. Additionally, the structures of premnaodoroside F and premnaodoroside G have not been drawn, since they are constituted by two isomers.

## 3. Chemophenetic Evaluation of *Bis*-Iridoids

As Table 1 clearly displays, *bis*-iridoids have been found in many families: Apiaceae Lindl., Aquifoliaceae Bercht. & J.Presl, Bignoniaceae Juss., Calyceraceae R.Br. ex Rich., Caprifoliaceae Juss., Cornaceae Bercht. ex J.Presl, Gentianaceae Juss., Goodeniaceae R.Br., Lamiaceae Martinov, Loasaceae Juss., Loganiaceae R.Br. ex Mart., Oleaceae Hoffmanns. & Link, Orobanchaceae Vent., Plantaginaceae Juss., Rubiaceae Juss., Sarraceniaceae Dumort., Stemonuraceae Kårehed and Viburnaceae Raf. Their highest occurrence is in Rubiaceae, reported from fourteen different genera (*Adina* Salisb., *Catunaregam* Wolf, *Coelospermum* Blume, *Coptosapelta* Korth., *Galium* L., *Gardenia* J.Ellis, *Gynochthodes* Blume, *Lasianthus* Jack, *Morinda* L., *Mussaenda* Burm. ex L., *Neonauclea* Merr., *Paederia* L., *Palicourea* Aubl. and *Saprosma* Blume), whereas the lowest was in ten families, having been reported in one only genus each (Apiaceae: *Heracleum* L.; Aquifoliaceae: *Ilex* L.; Calyceraceae: *Acicarpha* Juss.; Cornaceae: *Cornus* L.; Cyperaceae: *Cyperus* L.; Goodeniaceae: *Scaevola* L.; Loganiaceae: *Strychnos* L.; Orobanchaceae: *Pedicularis* L.; Sarraceniaceae: *Sarracenia* Tourn. ex L.; Stemonuraceae: *Cantleya* Ridl.; Viburnaceae: *Viburnum* L.). *Bis*-iridoids have been reported in two Bignoniaceae genera (*Argylia* D.Don and *Handroanthus* Mattos), in twelve Caprifoliaceae genera (*Abelia* Gronov., *Cephalaria* Schrad., *Dipsacus* L., *Linnaea* Gronov., *Lomelosia* Raf., *Lonicera* L., *Patrinia* Juss., *Pterocephalus* Vaill. ex Adans., *Scabiosa* L., *Triosteum* L., *Triplostegia* Wall. ex DC. and *Valeriana* L.), in six Gentianaceae genera (*Centaurium* Hill, *Fagraea* Thunb., *Gentiana* Tourn. ex L., *Gentianella* Moench, *Swertia* L. and *Tripterospermum* Blume), in five Lamiaceae genera (*Caryopteris* Bunge, *Clinopodium* L., *Leonotis* (Pers.) R.Br. and *Premna* L., *Salvia* L.), in two Loasaceae genera (*Kissenia* R.Br. ex Endl. and *Loasa* Adans.); in seven Oleaceae genera (*Fraxinus* Tourn. ex L., *Jasminum* L., *Ligustrum* L., *Olea* L., *Osmanthus* Lour., *Picconia* DC. and *Syringa* L.) and in six Plantaginaceae genera (*Anarrhinum* Desf., *Globularia* Tourn. ex L., *Kickxia* Dumort., *Linaria* Mill., *Picrorhiza* Royle ex Benth. and *Wulfenia* Jacq.). This occurrence is not in perfect agreement with the one for simple iridoids [242]. In fact, several families (Acanthaceae Juss., Actinidiaceae Gilg & Werderm., Apocynaceae Juss., Asteraceae Giseke, Cardiopteridaceae Blume, Celastraceae R.Br., Centroplacaceae Doweld & Reveal, Columelliaceae D.Don, Cucurbitaceae Juss., Cyperaceae Juss., Daphniphyllaceae Müll.Arg., Ericaceae Juss., Escalloniaceae R.Br. ex Dumort., Eucommiaceae Engl., Fabaceae Juss., Euphorbiaceae Juss., Fouquieriaceae DC., Garryaceae Lindl., Gel-miaceae Struwe & V.A.Albert, Gri-liniaceae J.R.Forst. & G.Forst. ex A.Cunn., Hamamelidaceae R.Br, Hydrangeaceae Dumort., Icacinaceae Miers, Lentibulariaceae Rich., Malpighiaceae Juss., Malvaceae Juss., Martyniaceae Horan., Meliaceae Juss., Menyanthaceae Dumort., Metteniusaceae H.Karst. ex Schnizl., Montiniaceae Nakai, Nyssaceae Juss. ex Dumort., Passifloraceae Juss. ex Rous-l, Paulowniaceae Nakai, Pedaliaceae R.Br., Roridulaceae Martinov, Salicaceae Mirb., Sarraceniaceae Dumort., Scrophulariaceae Juss., Stilbaceae Kunth, Stylidiaceae R.Br. Symplocaceae Desf. and Verbenaceae J.St.-Hil.) are absent from Table 1, as well as a myriad of genera [242,243,244,245], and this clearly demonstrates that *bis*-iridoids must be separately considered from simple iridoids for biochemical, chemophenetic and pharmacological purposes and that their biosynthesis is only due to genetic factors and not to a combination of genetic and environmental factors.

Simple iridoids are generally considered as chemophenetic markers at different systematic levels from subspecies to orders [242]. The order with the highest occurrence of *bis*-iridoids is Lamiales, presenting a certain parallelism with simple iridoids [242]. From a careful and exhaustive evaluation of Table 1, some chemophenetic markers among *bis*-iridoids could be individuated at different levels. In particular, given their distribution, cantleyoside, laciniatosides and sylvestrosides can be used as chemophenetic markers for the Caprifoliaceae family, GI3 and GI5 for the Oleaceae family, oleonuezhenide for the *Ligustrum* genus and (*Z*)-aldosecologanin and centauroside for the *Lonicera* genus. For what concerns the other compounds, some have been reported in single species, while others in too many. For this, at the moment, they do not have the necessary characteristics to act as chemophenetic markers. Yet, future phytochemical studies might be useful in this sense, providing further information.

## 4. Biological Activities of *Bis*-Iridoids

Table 2 displays the biological activities associated with *bis*-iridoids. These are divided according to the type of activity, considering the methods employed and the effectiveness values of *bis*-iridoids in comparison with the positive controls.

Only one hundred and fifty-nine *bis*-iridoids have been studied for their biological activities. The highest number of biological studies has been observed for sylvestroside I, whereas cantleyoside is the compound presenting the highest number of biological studies for the same type. Conversely, only one type of biological assay has been performed for several *bis*-iridoids. Among the types, not all of them have been performed with the enzymatic assay as the major one. Not all the *bis*-iridoids have shown biological activity, and some have shown activities only for some assays, with effectiveness values both higher and lower than the positive controls when present. No clear preference of *bis*-iridoids for a specific biological activity among the studied ones has been observed, given that they exert, at least, one, except immunosuppressive. However, *bis*-iridoids have mostly shown anti-inflammatory, antibacterial, antiviral and enzymatic inhibitory effects, which are in perfect agreement with those reported for simple iridoids [9,242]. In-depth structure—activity relation speeches are not so easy to perform at the moment, because biological studies on *bis*-iridoids have been few, too sectorial and generally not specific from this point of view. Nevertheless, a generic conclusion from the careful observation of Table 2 indicates that the presence and the type of substituent, as well as the type of sub-unity, greatly affect the activity and the effectiveness of *bis*-iridoids, as already observed for simple iridoids [9,242]. At the moment, the comparison of the effectiveness values between *bis*-iridoids and simple iridoids cannot be performed as well, for the same previous reasons but also because some *bis*-iridoids are unconventionally structured (there is no base structure to compare to), almost all *bis*-iridoids are constituted by different sub-units (it is impossible to establish the starting compound) and the bond between the sub-units of *bis*-iridoids transforms the base structure and modifies its geometry (the comparison may not be reliable due to possible different mechanisms of action). Under all these last aspects, it is obvious that *bis*-iridoids need to be further studied.

## 5. Conclusions

In this review paper, two hundred and eighty-eight *bis*-iridoids have been listed and detailed with their occurrence in plants and the methodologies of extraction, isolation and identification and also one hundred and fifty-nine out of these with their biological activities. The *bis*-iridoids reported so far in the literature are mainly characterized by the link between two *seco*-iridoids sub-units under the structural profile and mostly exert anti-inflammatory, antibacterial, antiviral and enzymatic inhibitory activities, both with good and low effectiveness values. The chemophenetic evaluation has allowed to individuate cantleyoside, laciniatosides, sylvestrosides and GI3 and GI5 as chemophenetic markers for the Caprifoliaceae and Oleaceae families, respectively, and oleonuezhenide and (*Z*)-aldosecologanin and centauroside as chemophenetic markers for the *Ligustrum* and *Lonicera* genus, respectively. Yet, many aspects of *bis*-iridoids are still to be discovered, elucidated and completed, and this review paper, meaning to work as a multi-comprehensive database for the future, has clearly proven this.

## Figures and Tables

**Figure 1:**
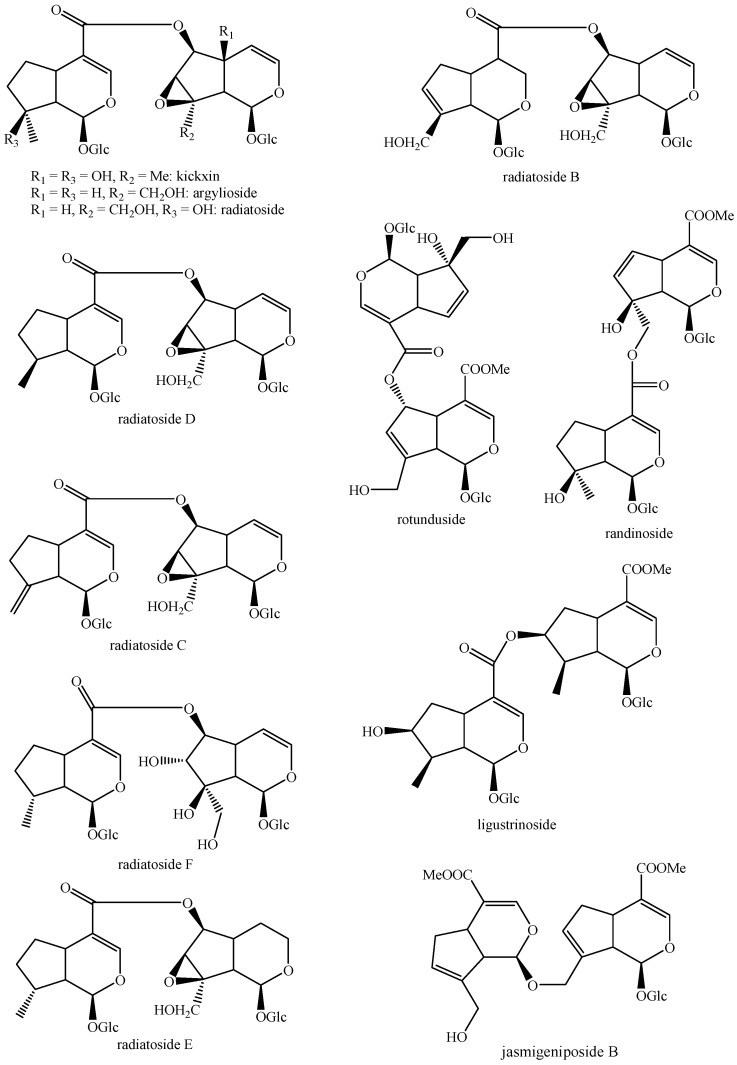
Structures of *bis*-iridoids in plants—iridoid plus iridoid part 1.

**Figure 2:**
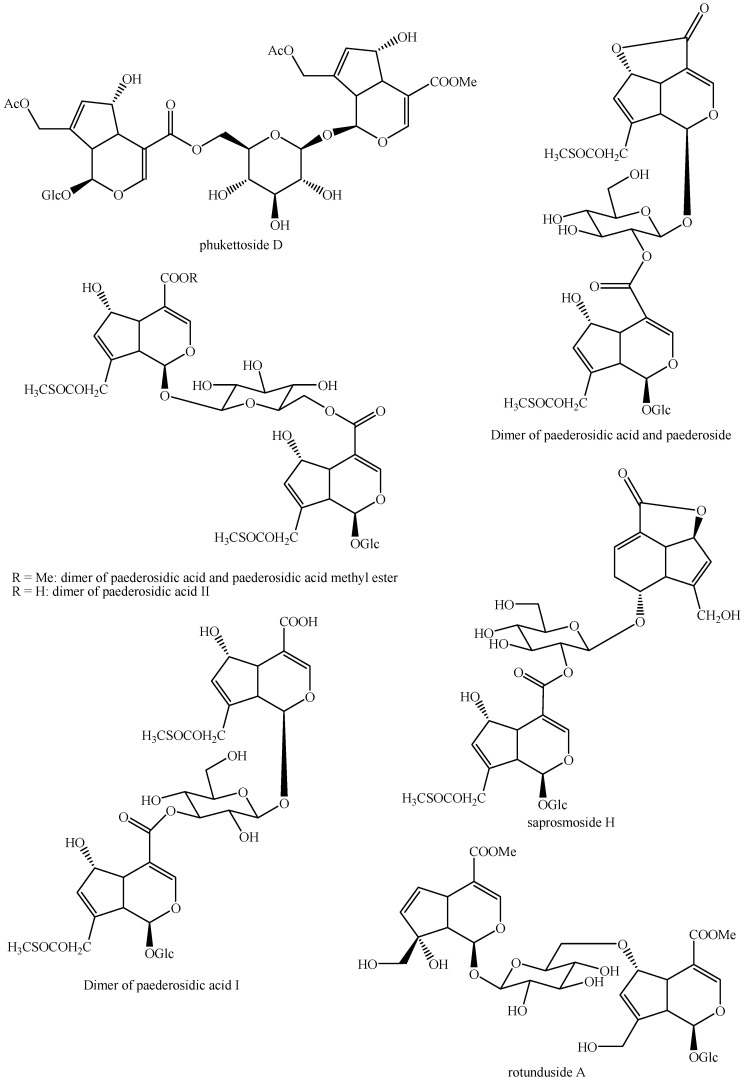
Structures of *bis*-iridoids in plants—iridoid plus iridoid part 2.

**Figure 3:**
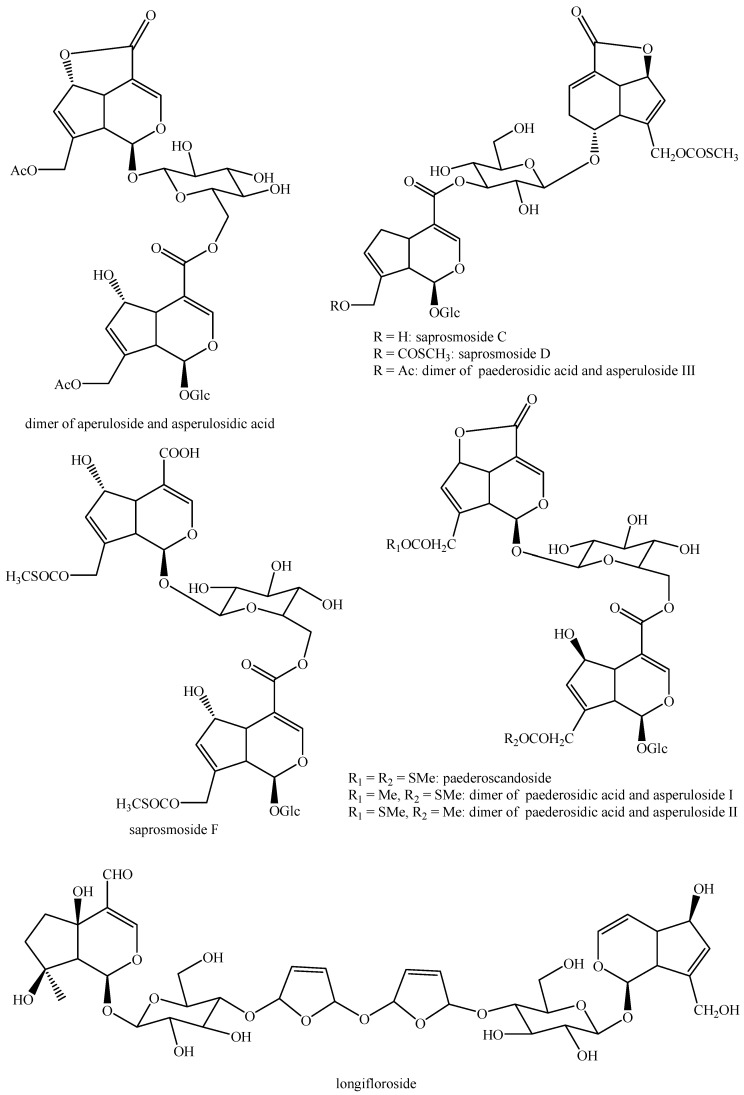
Structures of *bis*-iridoids in plants—iridoid plus iridoid part 3.

**Figure 4:**
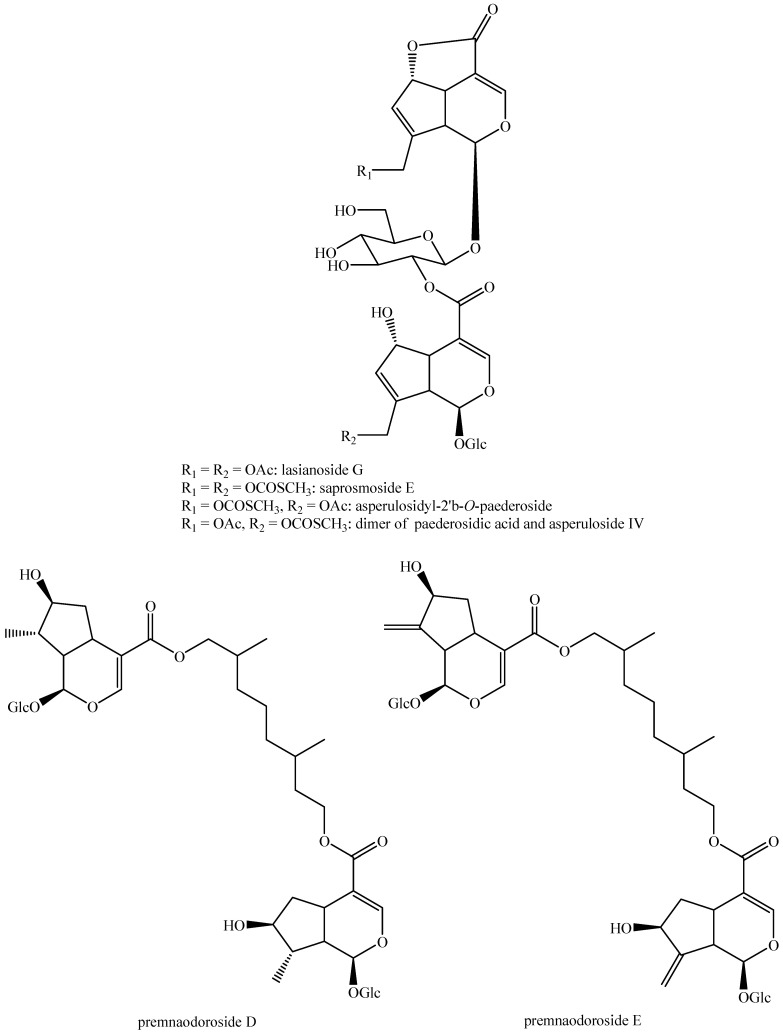
Structures of *bis*-iridoids in plants—iridoid plus iridoid part 4.

**Figure 5:**
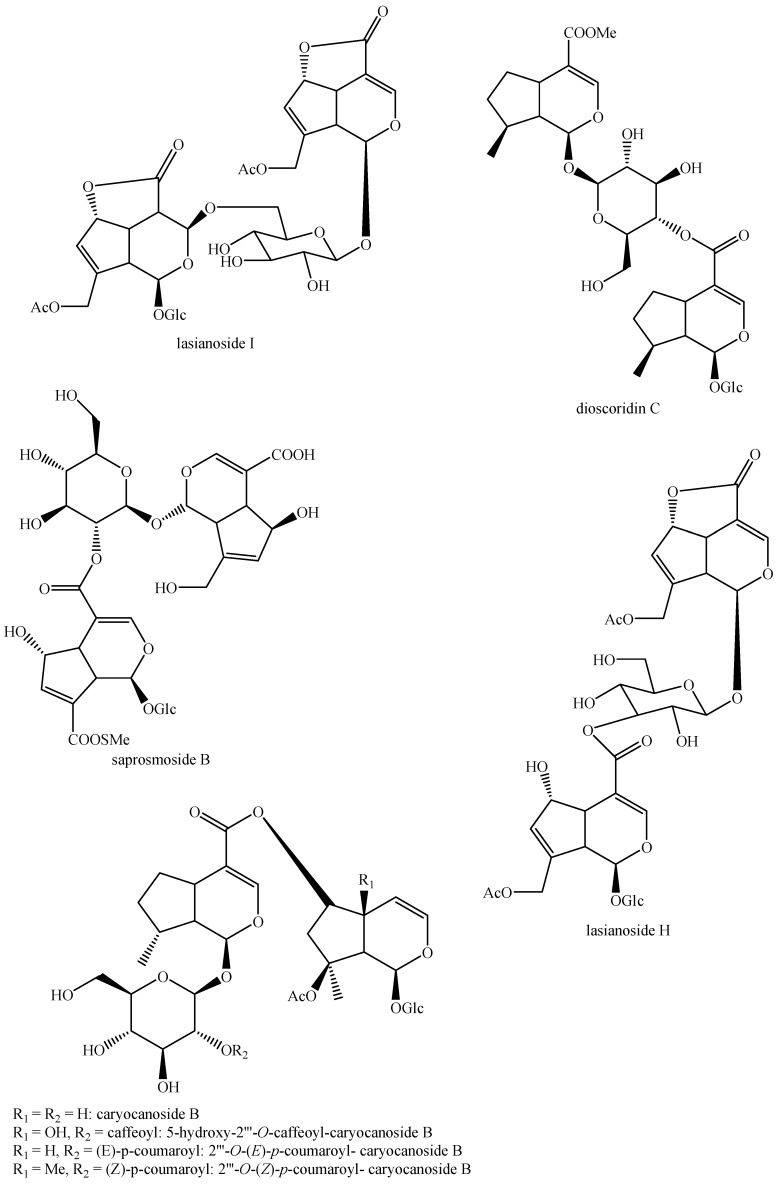
Structures of *bis*-iridoids in plants—iridoid plus iridoid part 5.

**Figure 6:**
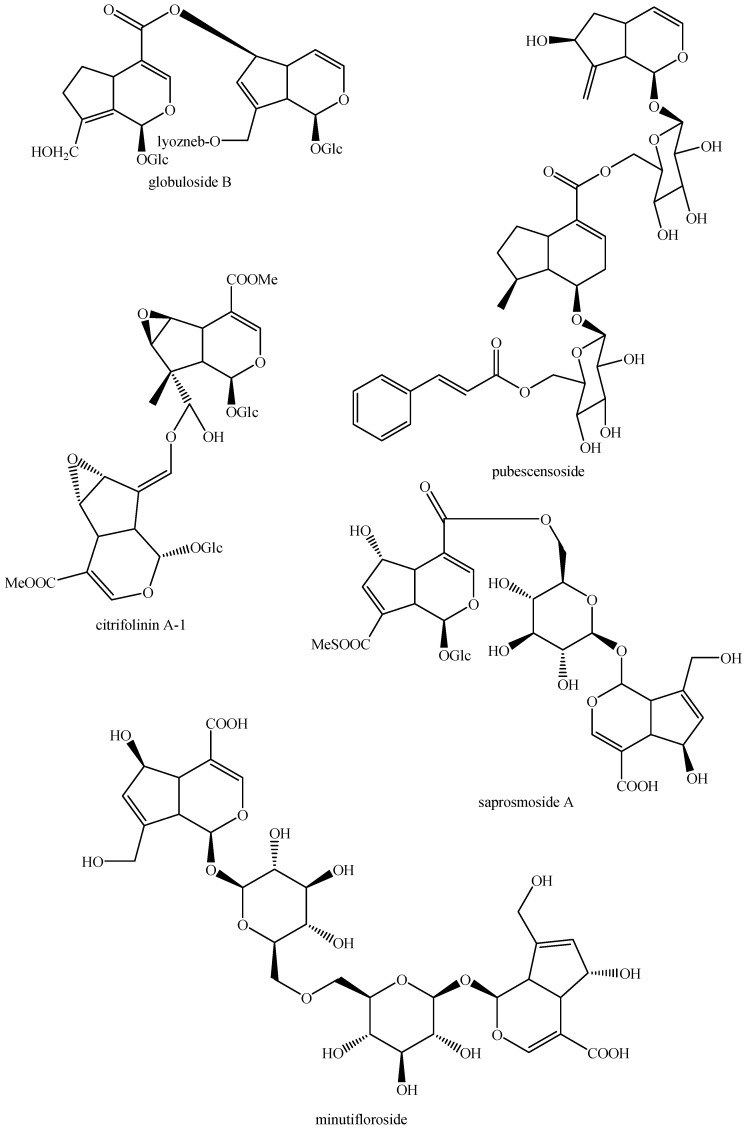
Structures of *bis*-iridoids in plants—iridoid plus iridoid part 6.

**Figure 7:**
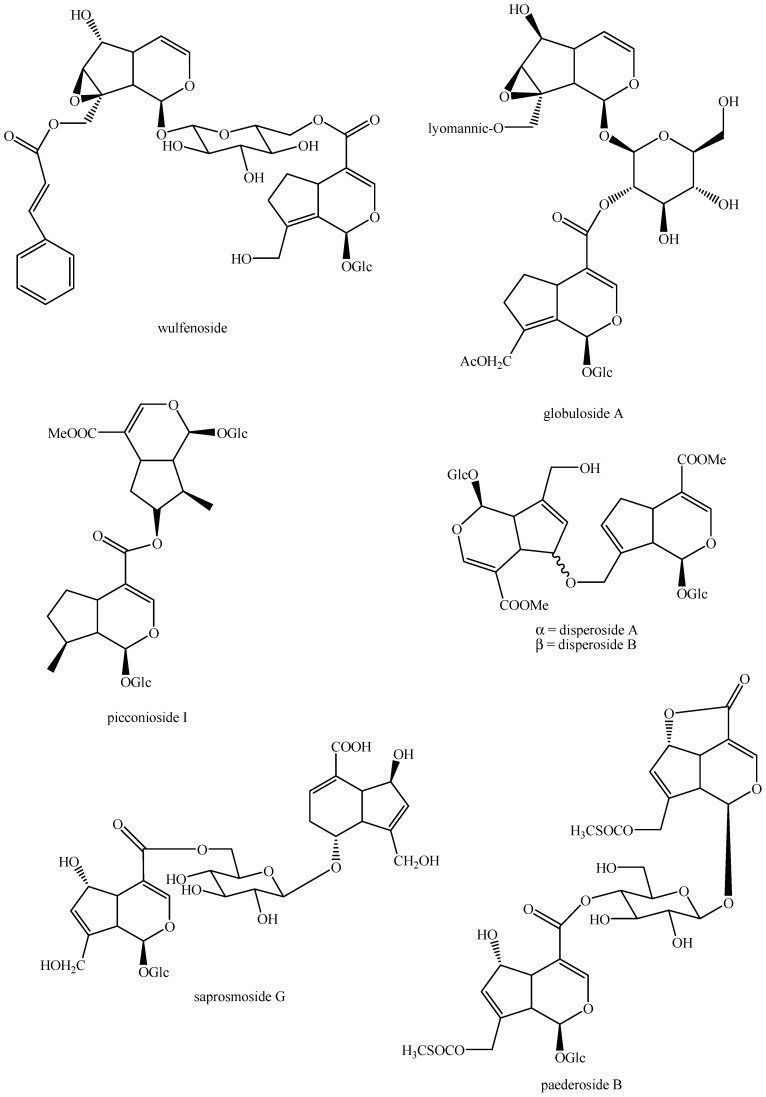
Structures of *bis*-iridoids in plants—iridoid plus iridoid part 7.

**Figure 8:**
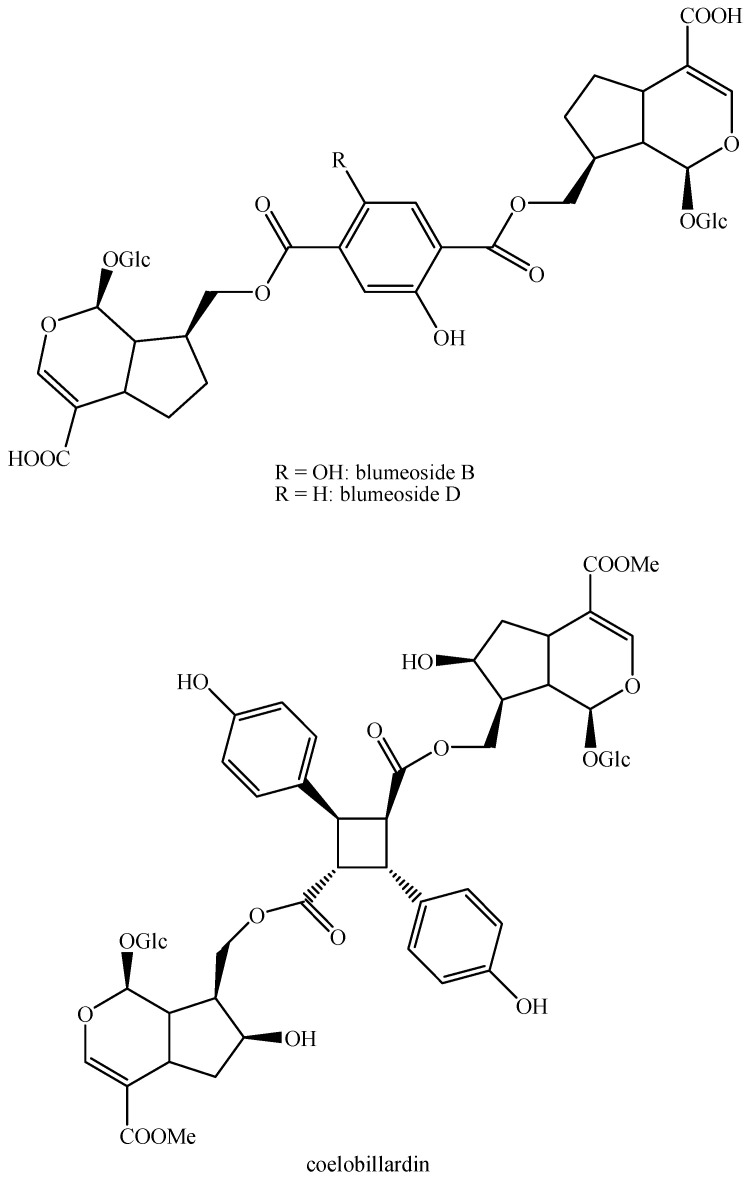
Structures of *bis*-iridoids in plants—iridoid plus iridoid part 8.

**Figure 9:**
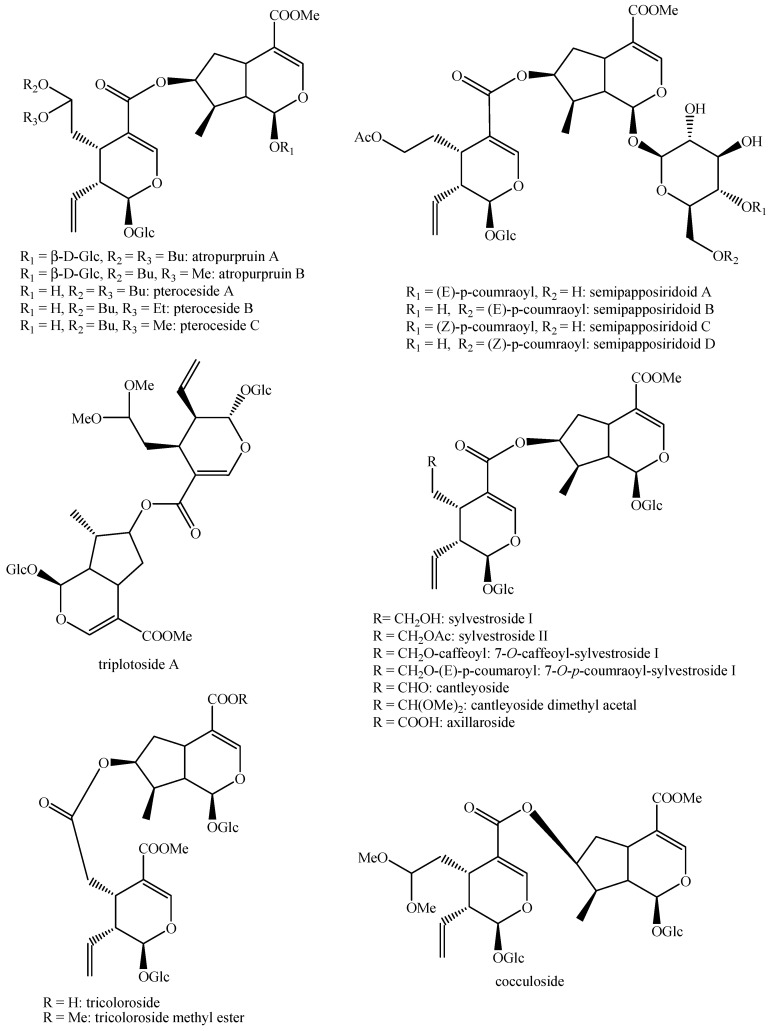
Structures of *bis*-iridoids in plants—iridoid plus *seco*-iridoid part 1.

**Figure 10:**
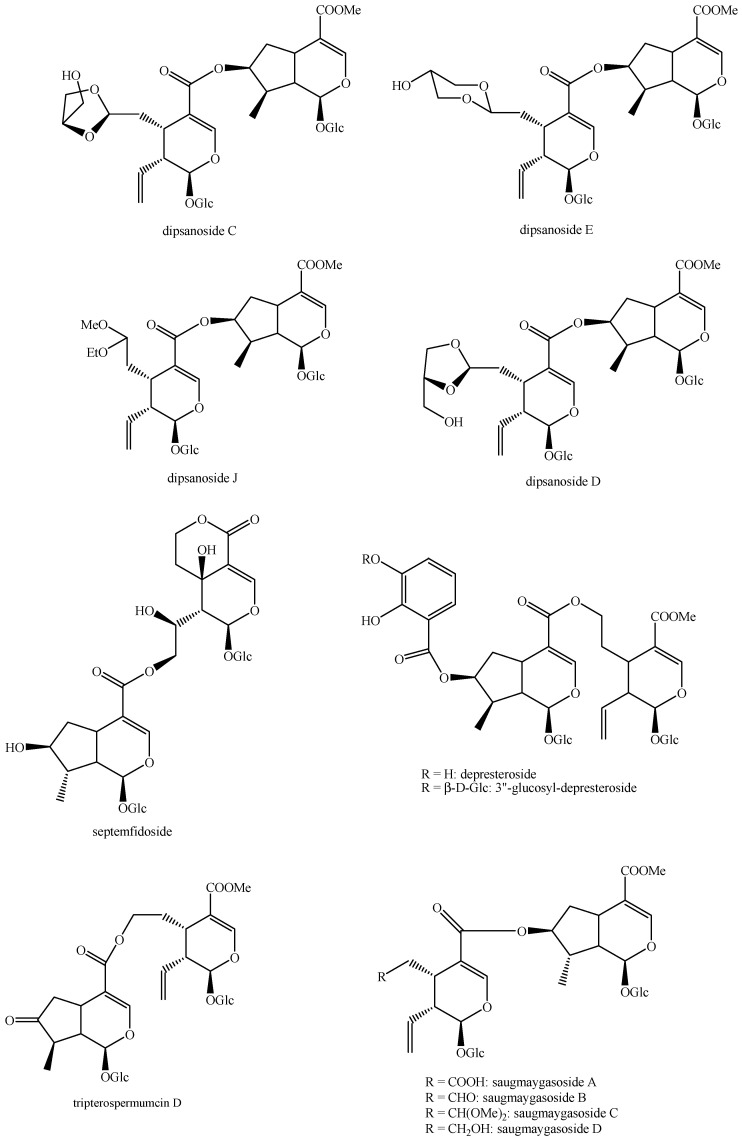
Structures of *bis*-iridoids in plants—iridoid plus *seco*-iridoid part 2.

**Figure 11:**
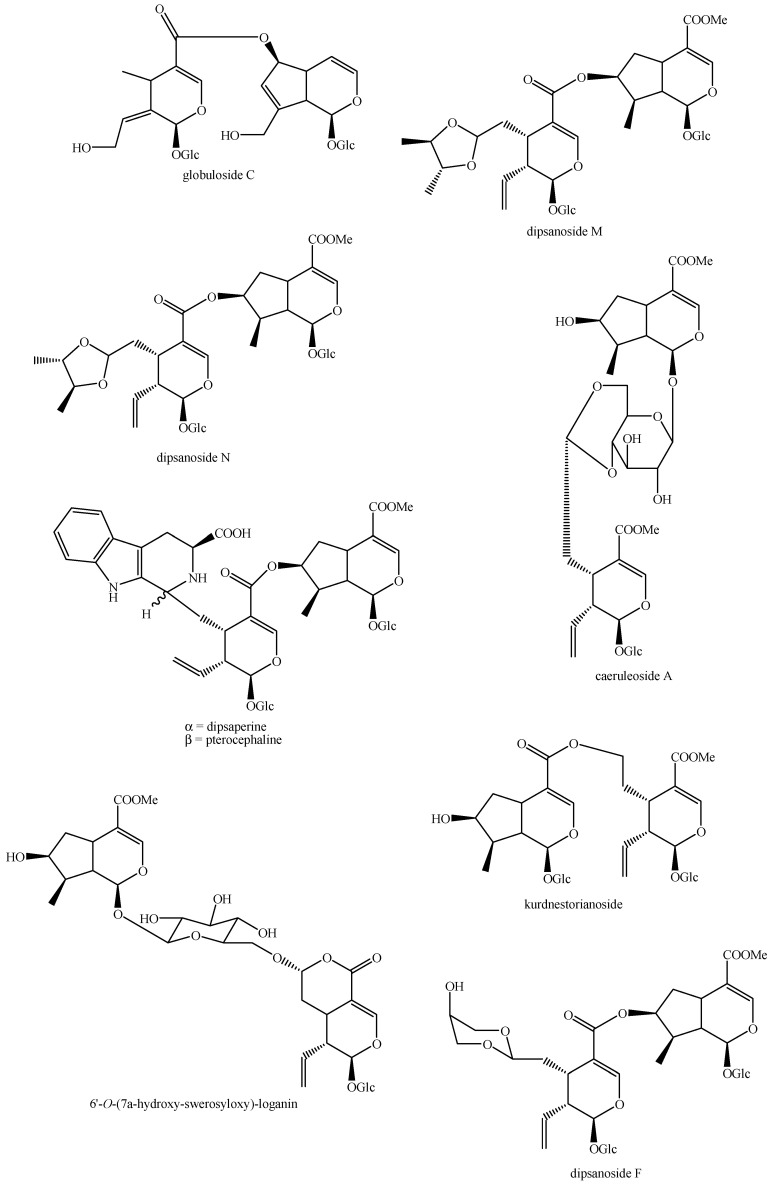
Structures of *bis*-iridoids in plants—iridoid plus *seco*-iridoid part 3.

**Figure 12:**
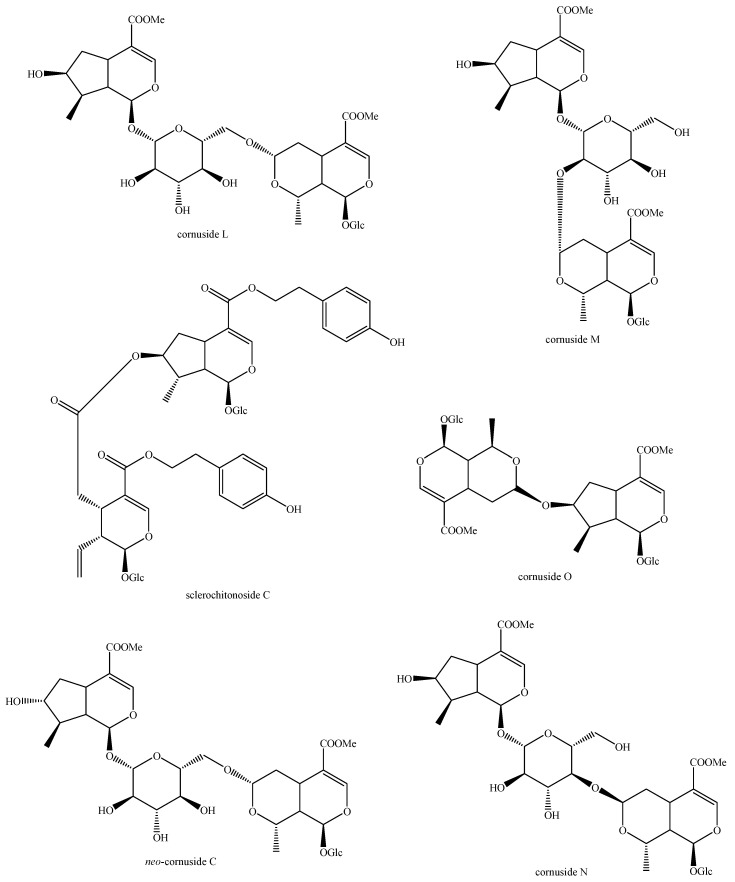
Structures of *bis*-iridoids in plants—iridoid plus *seco*-iridoid part 4.

**Figure 13:**
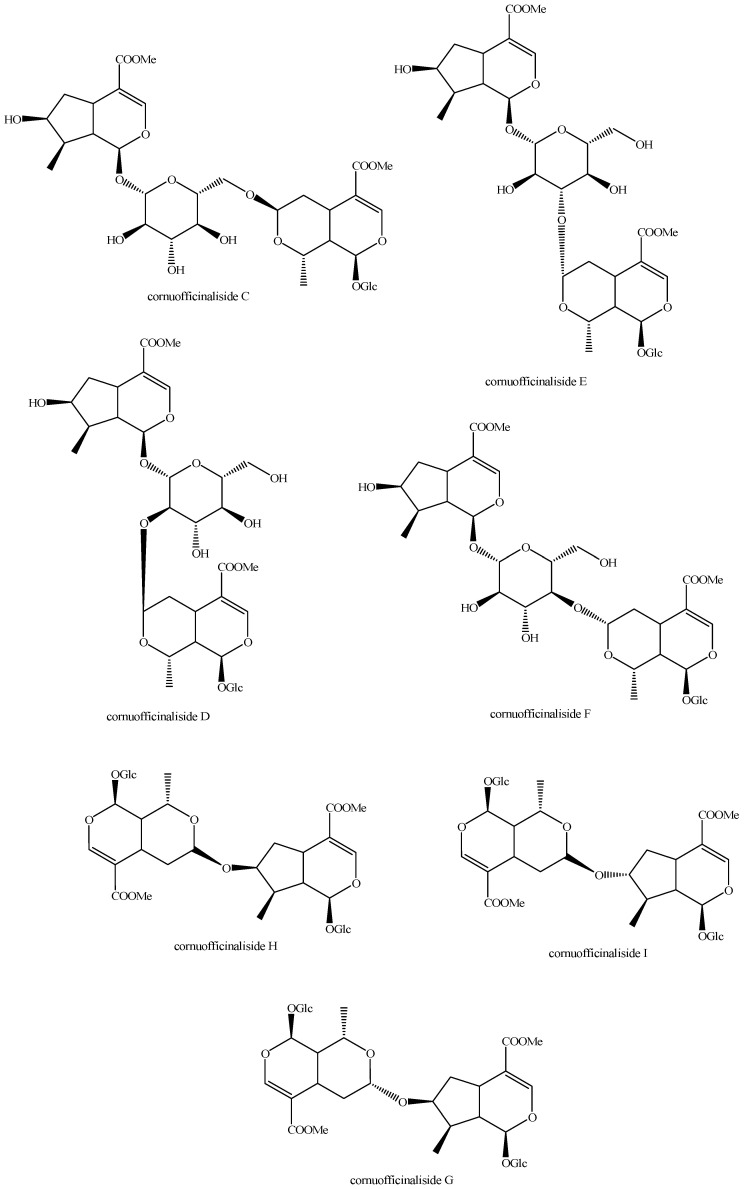
Structures of *bis*-iridoids in plants—iridoid plus *seco*-iridoid part 5.

**Figure 14:**
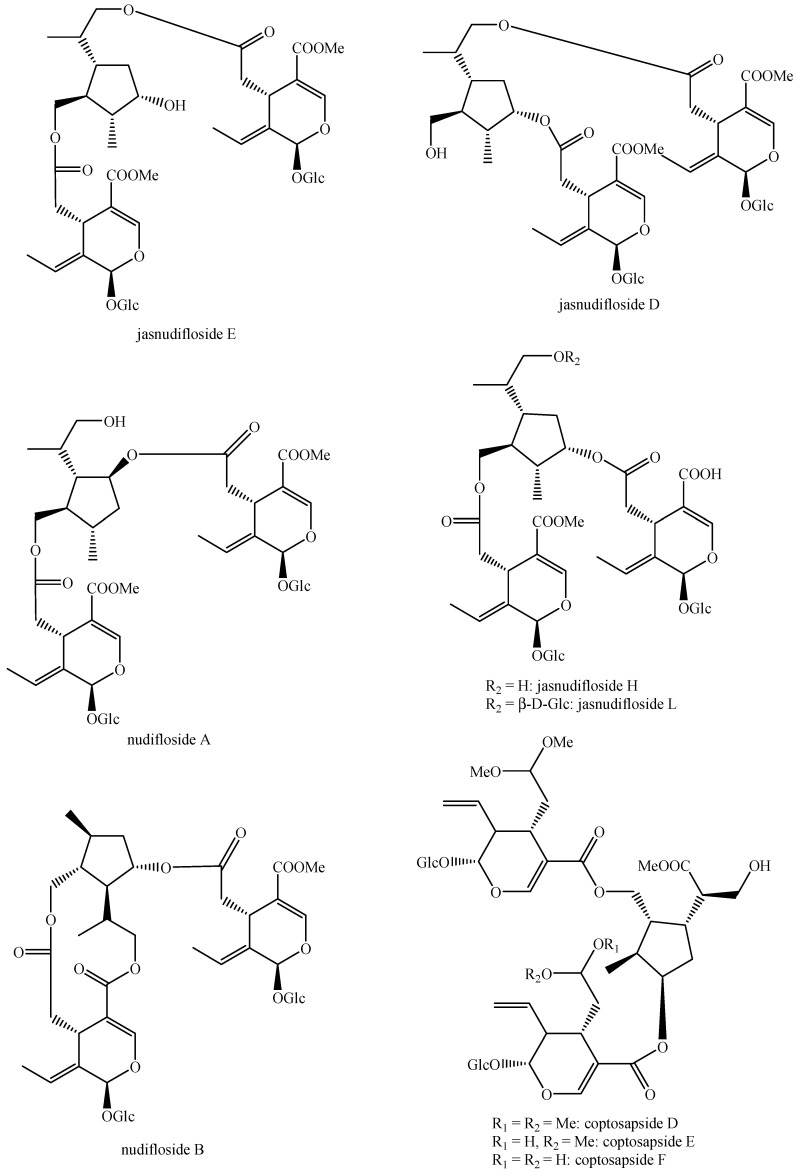
Structures of *bis*-iridoids in plants—*seco*-iridoid plus *seco*-iridoid part 1.

**Figure 15:**
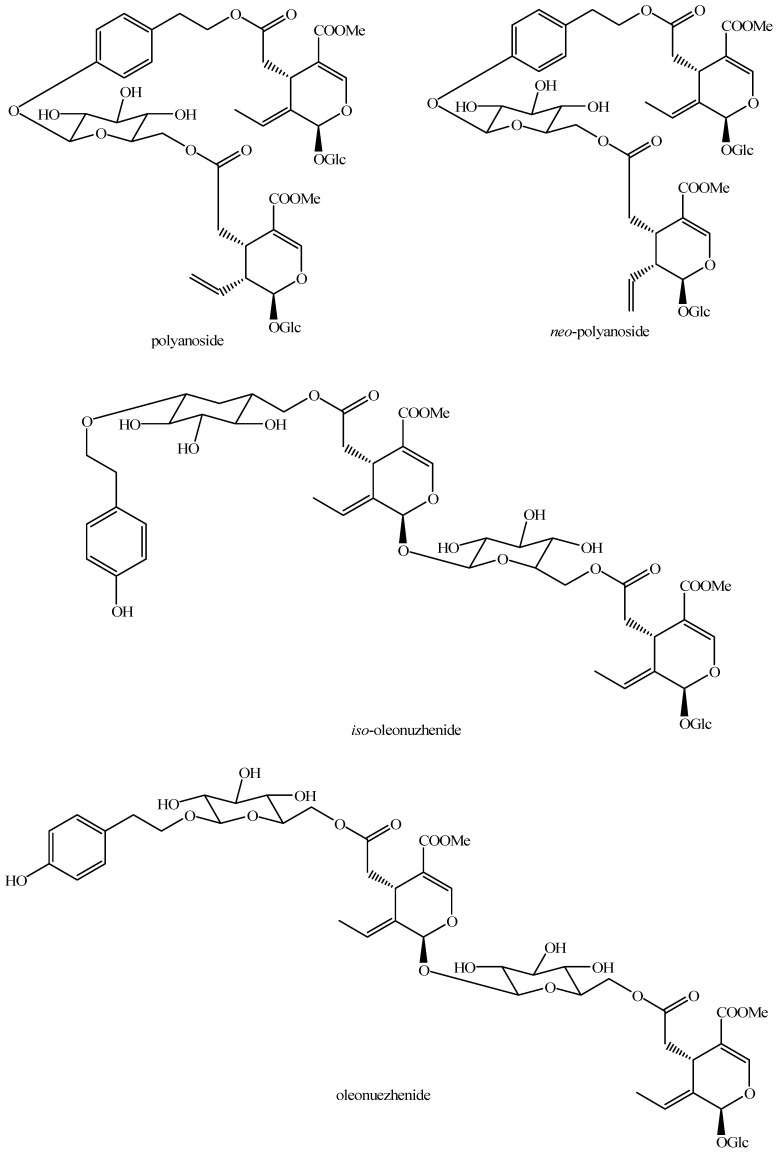
Structures of *bis*-iridoids in plants—*seco*-iridoid plus *seco*-iridoid part 2.

**Figure 16:**
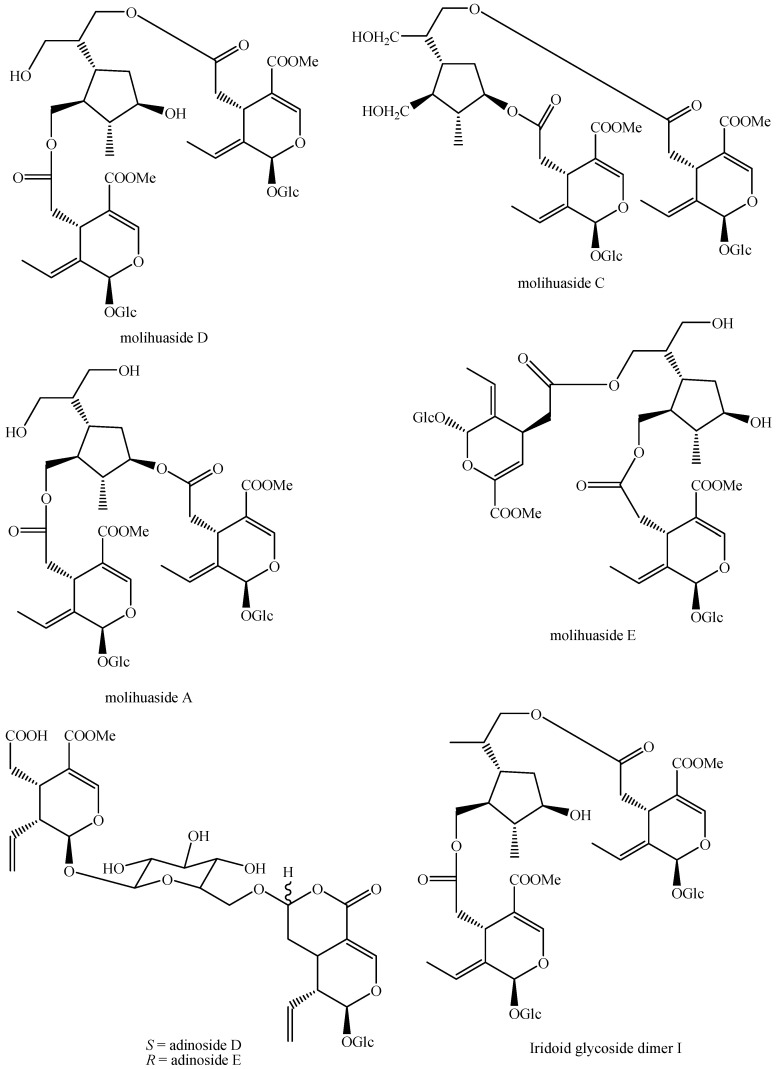
Structures of *bis*-iridoids in plants—*seco*-iridoid plus *seco*-iridoid part 3.

**Figure 17:**
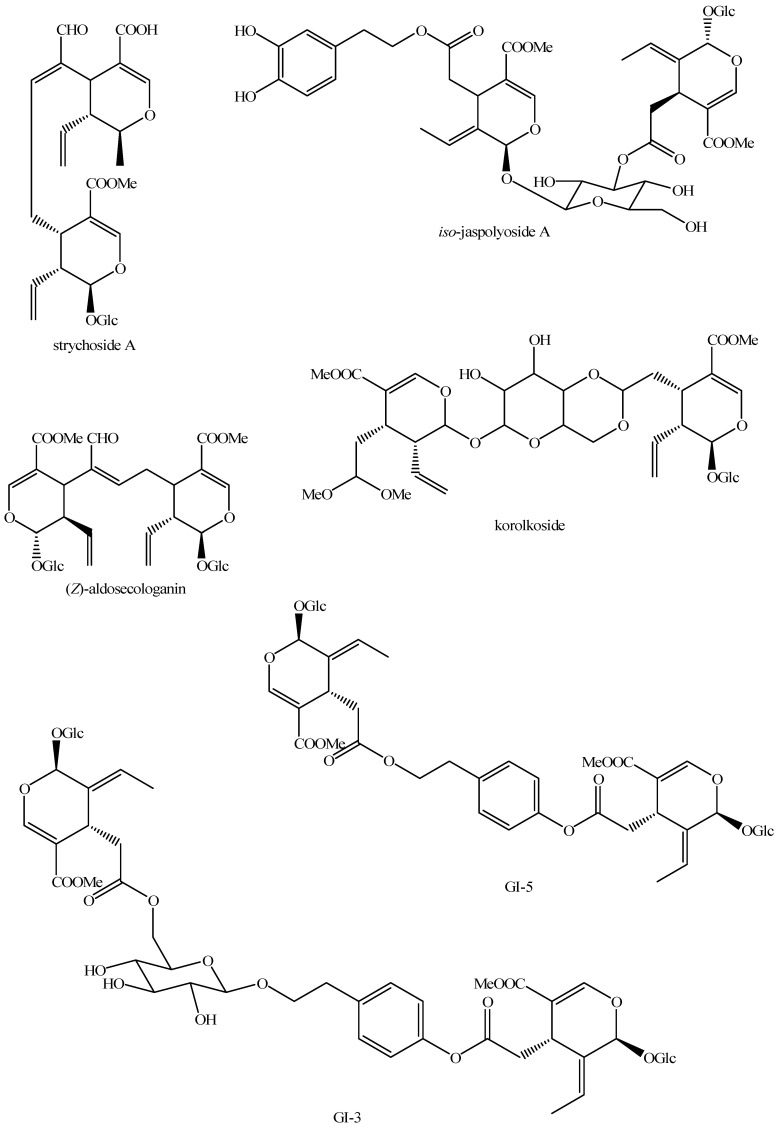
Structures of *bis*-iridoids in plants—*seco*-iridoid plus *seco*-iridoid part 4.

**Figure 18:**
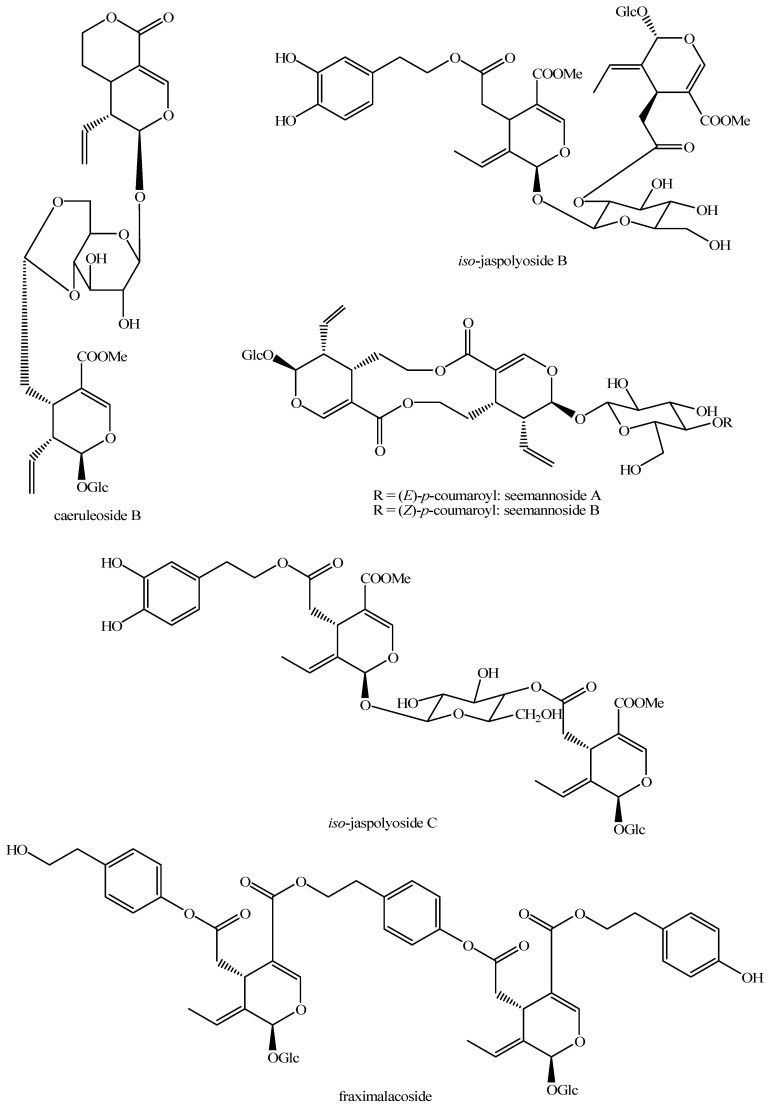
Structures *bis*-iridoids in plants—*seco*-iridoid plus *seco*-iridoid part 5.

**Figure 19:**
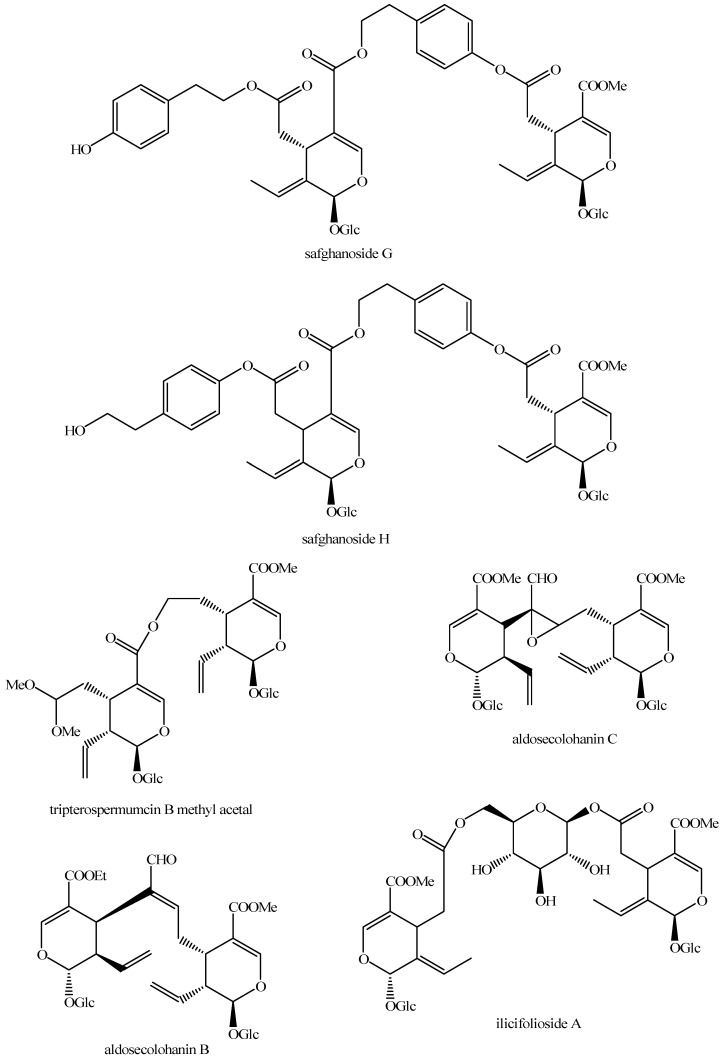
Structures of *bis*-iridoids in plants—*seco*-iridoid plus *seco*-iridoid part 6.

**Figure 20:**
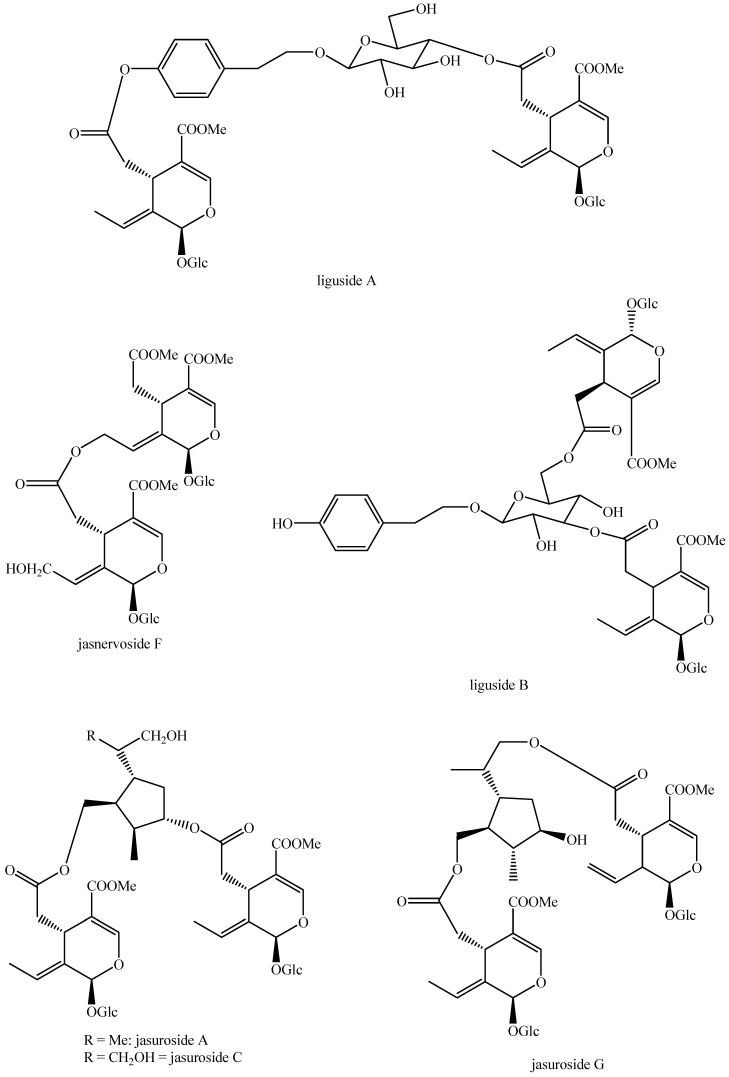
Structures of *bis*-iridoids in plants—*seco*-iridoid plus *seco*-iridoid part 7.

**Figure 21:**
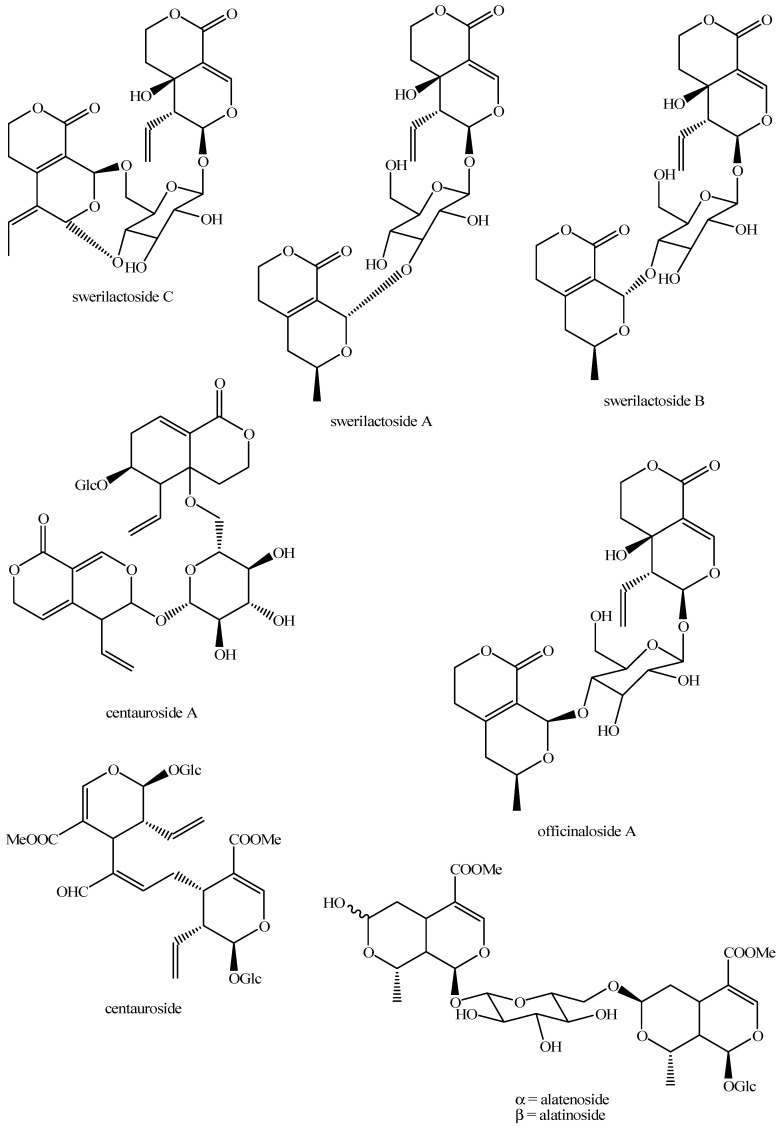
Structures of *bis*-iridoids in plants—*seco*-iridoid plus *seco*-iridoid part 8.

**Figure 22:**
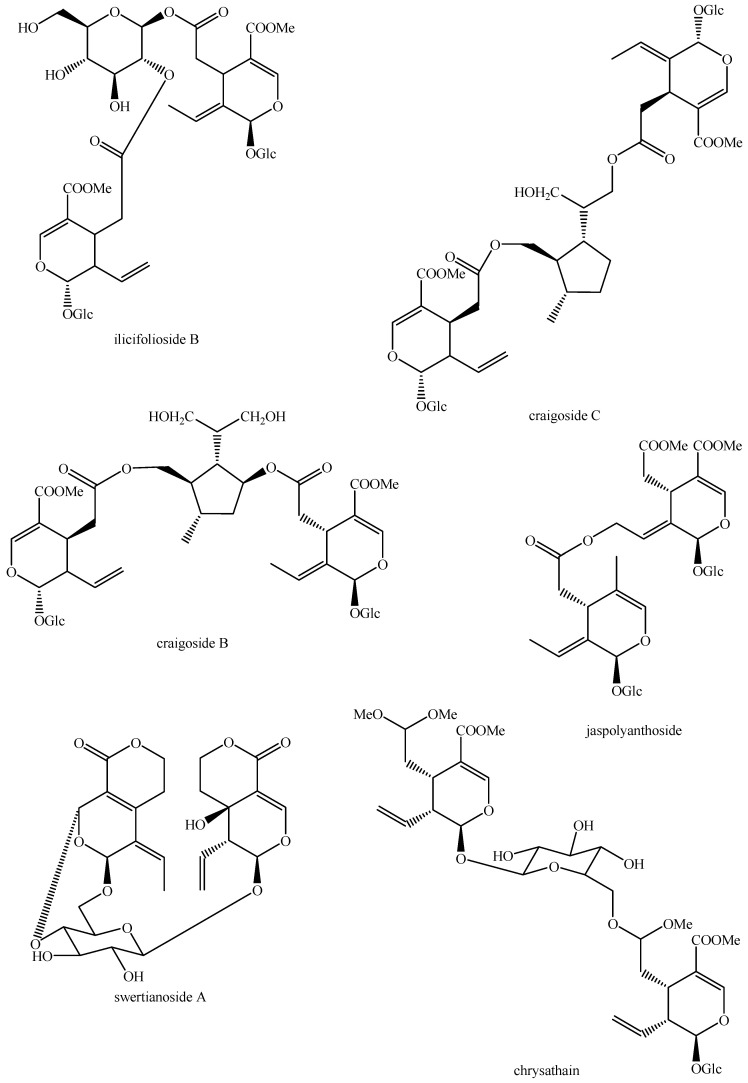
Structures of *bis*-iridoids in plants—*seco*-iridoid plus *seco*-iridoid part 9.

**Figure 23:**
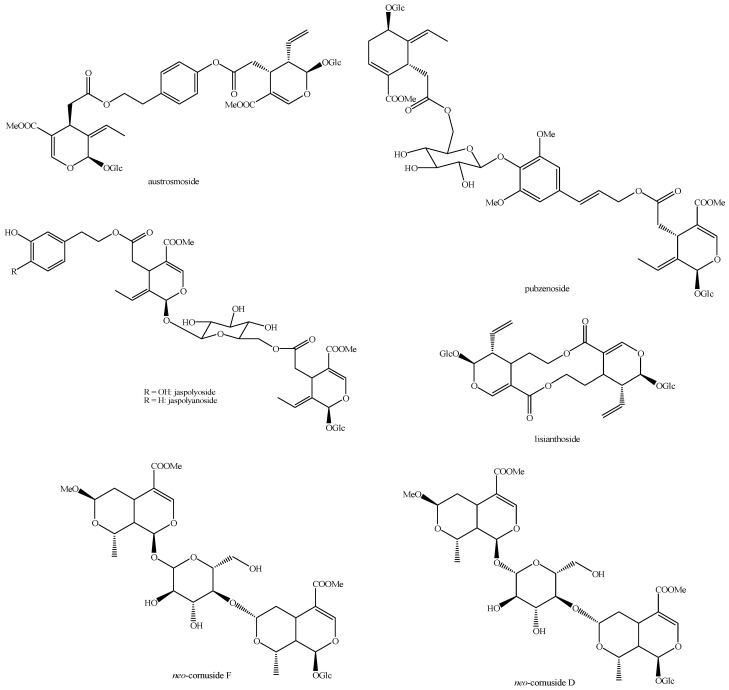
Structures of *bis*-iridoids in plants—*seco*-iridoid plus *seco*-iridoid part 10.

**Figure 24:**
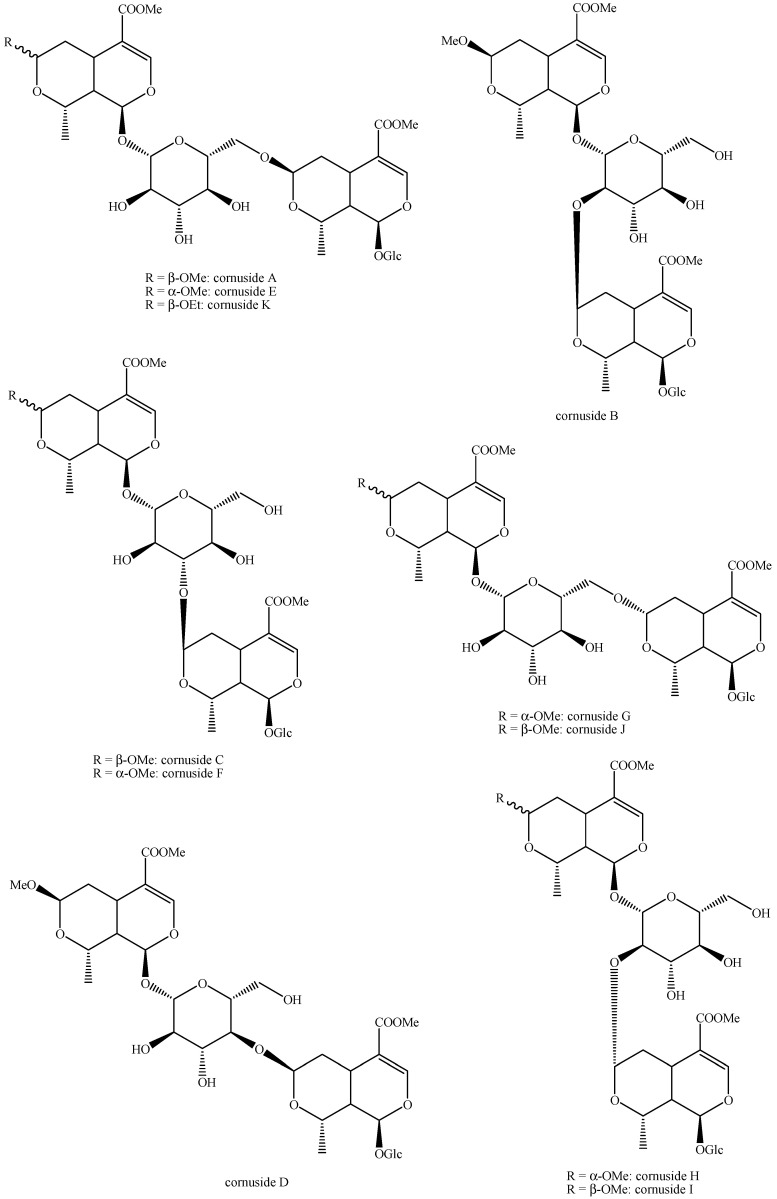
Structures of *bis*-iridoids in plants—*seco*-iridoid plus *seco*-iridoid part 11.

**Figure 25:**
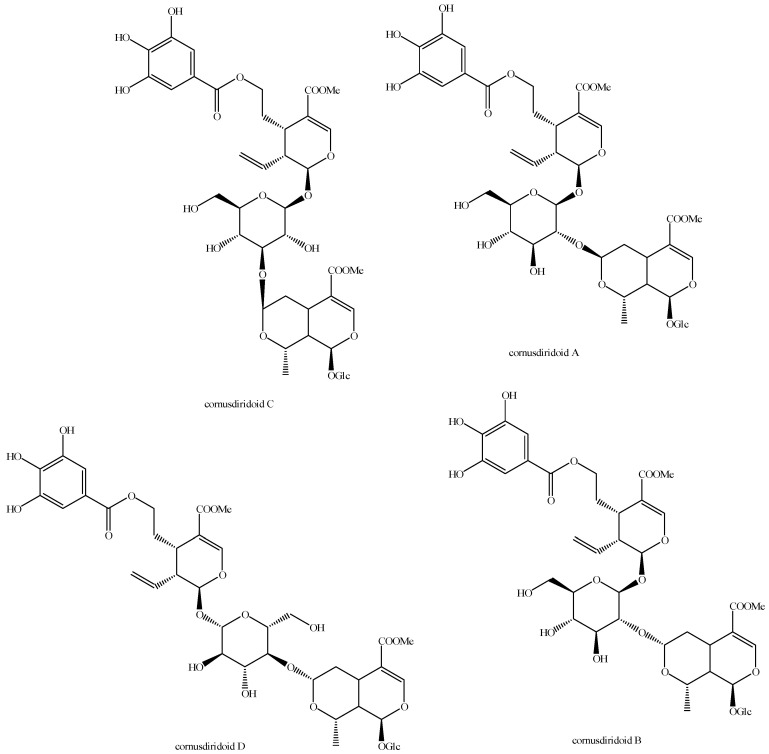
Structures of *bis*-iridoids in plants—*seco*-iridoid plus *seco*-iridoid part 12.

**Figure 26:**
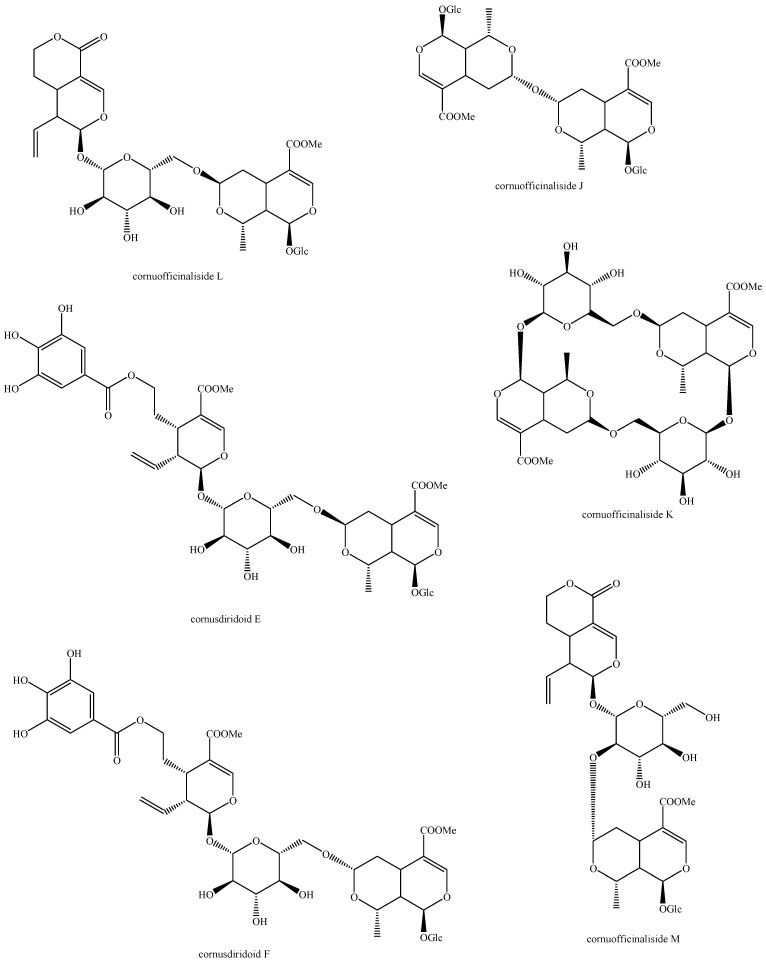
Structures of *bis*-iridoids in plants—*seco*-iridoid plus *seco*-iridoid part 13.

**Figure 27:**
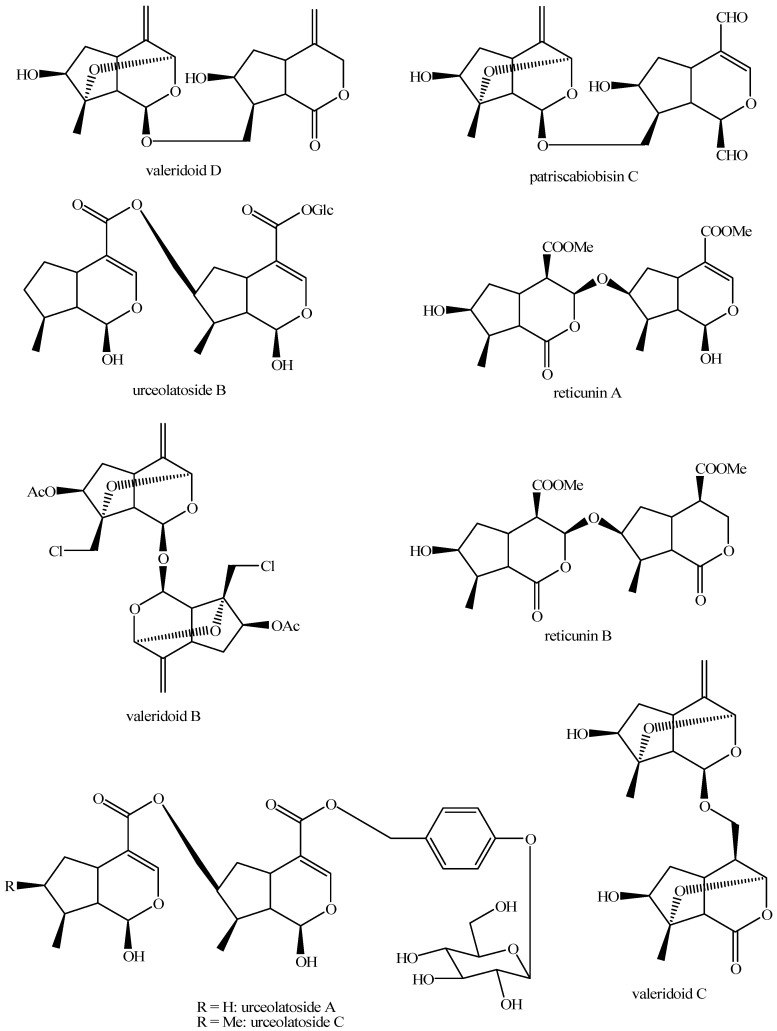
Structures of *bis*-iridoids in plants—non-glucosidic iridoid plus non-glucosidic iridoid.

**Figure 28:**
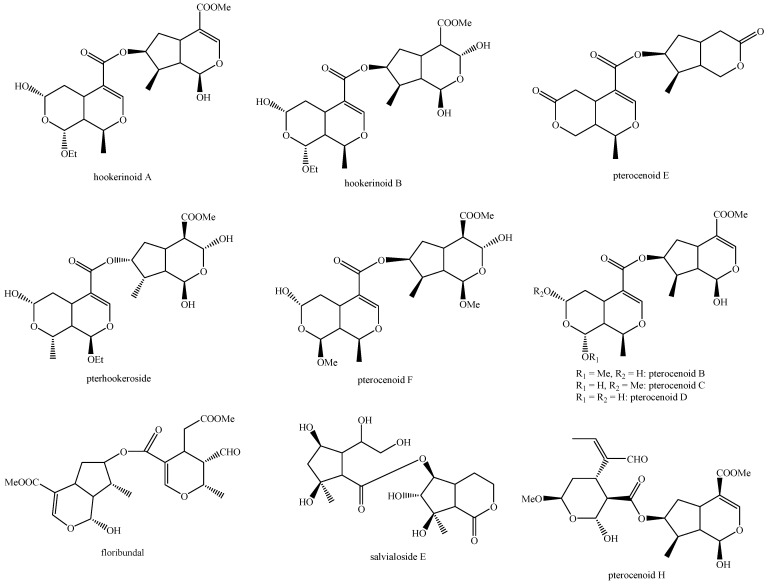
Structures of *bis*-iridoids in plants—non-glucosidic iridoid plus non-glucosidic *seco*-iridoid.

**Figure 29:**
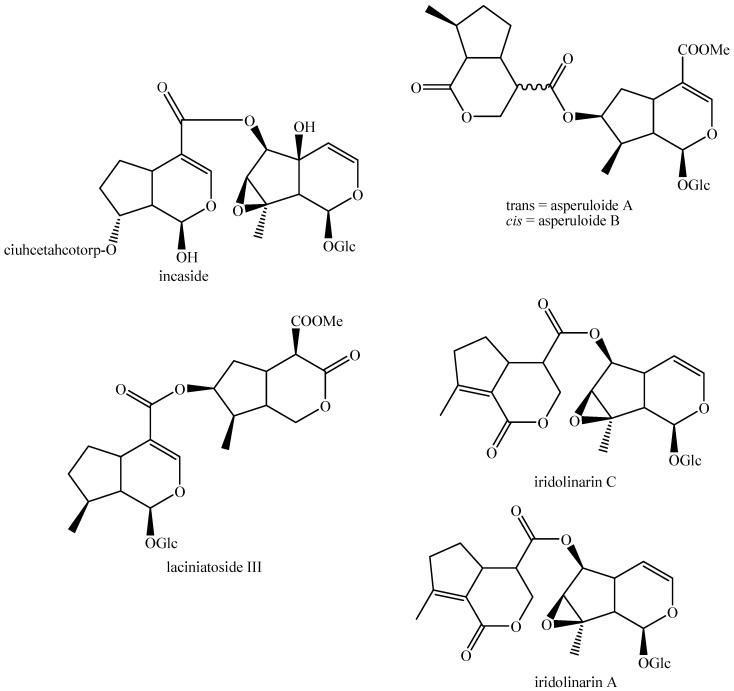
Structures of *bis*-iridoids in plants—iridoid plus non-glucosidic iridoid.

**Figure 30:**
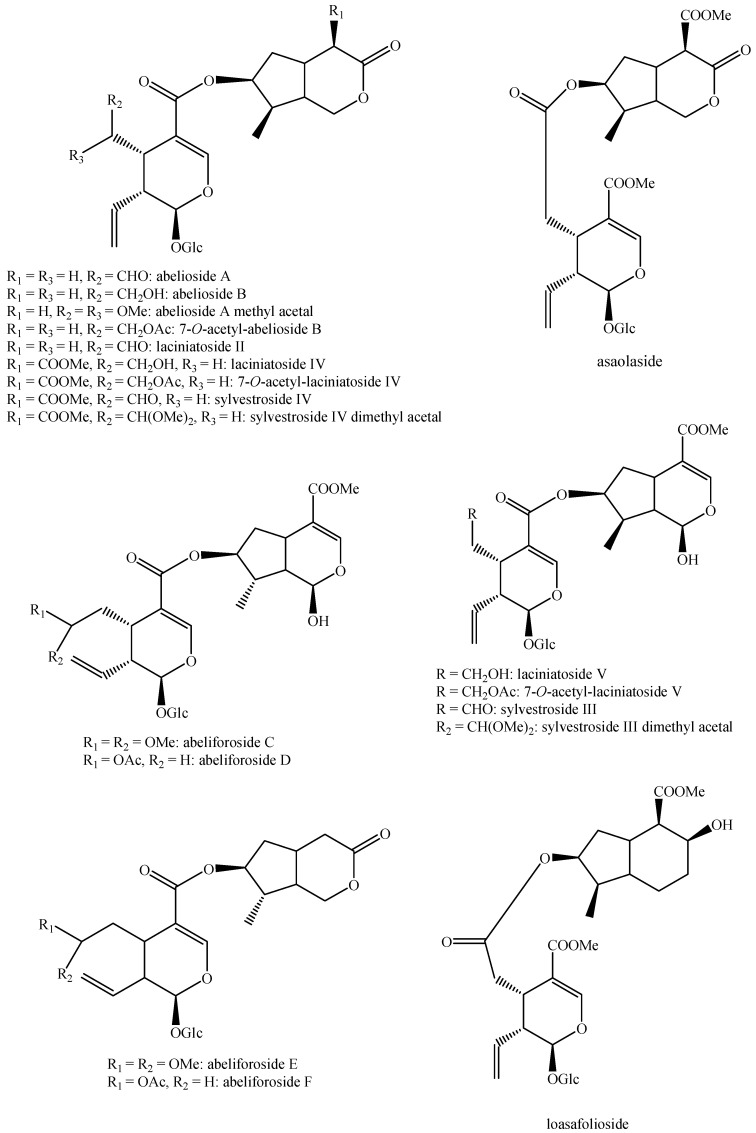
Structures of *bis*-iridoids in plants—non-glucosidic iridoid plus *seco*-iridoid part 1.

**Figure 31:**
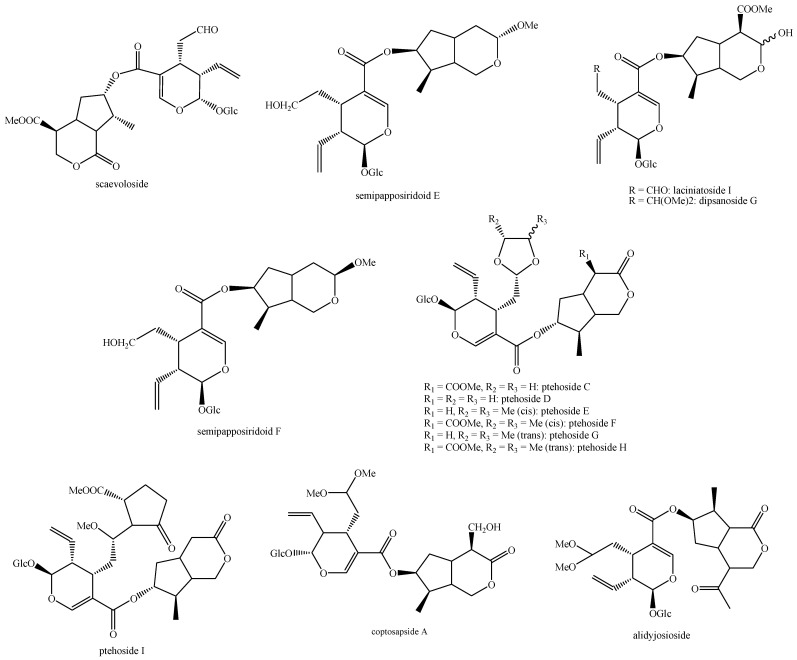
Structures of *bis*-iridoids in plants—non-glucosidic iridoid plus *seco*-iridoid part 2.

**Figure 32:**
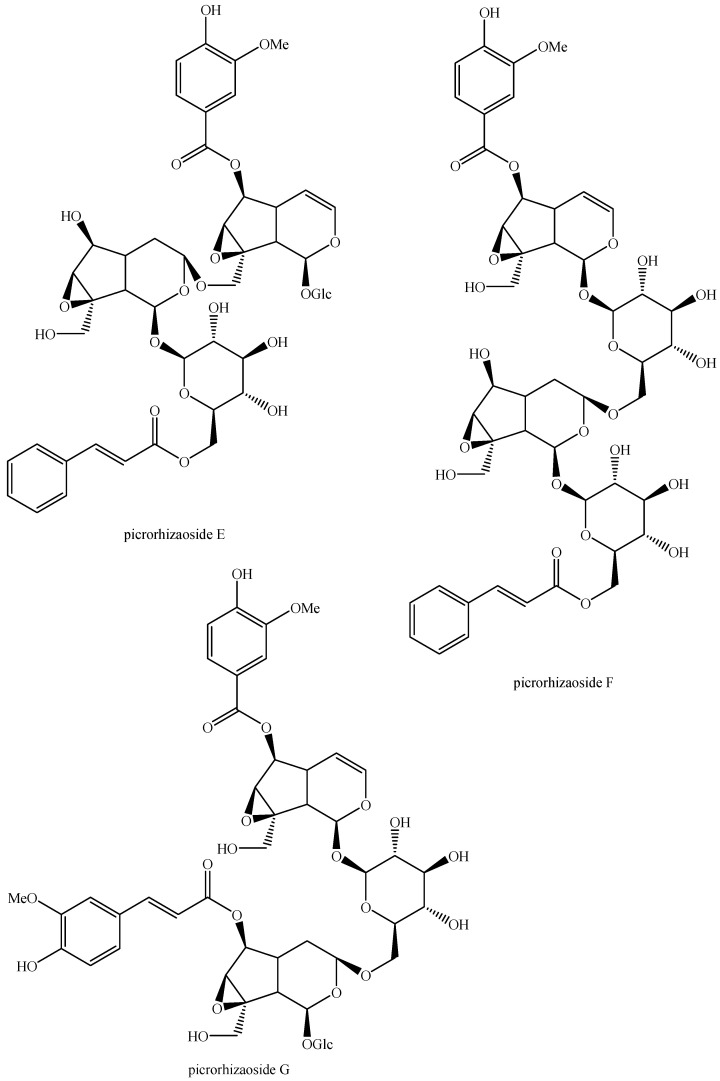
Structures of non-conventional *bis*-iridoids in plants—part 1.

**Figure 33:**
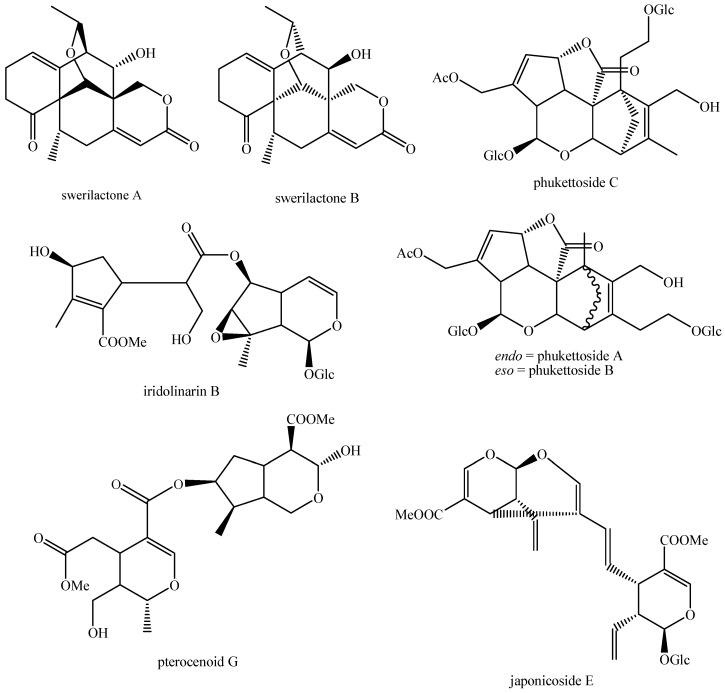
Structures of non-conventional *bis*-iridoids in plants—part 2.

**Figure 34:**
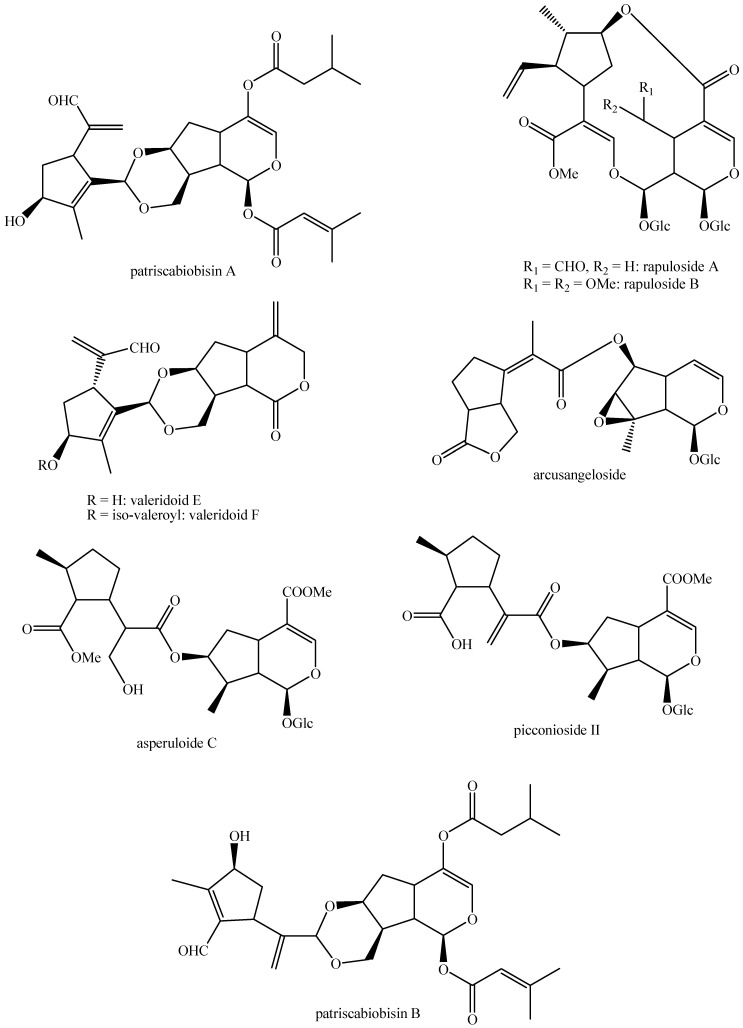
Structures of non-conventional *bis*-iridoids in plants—part 3.

**Figure 35:**
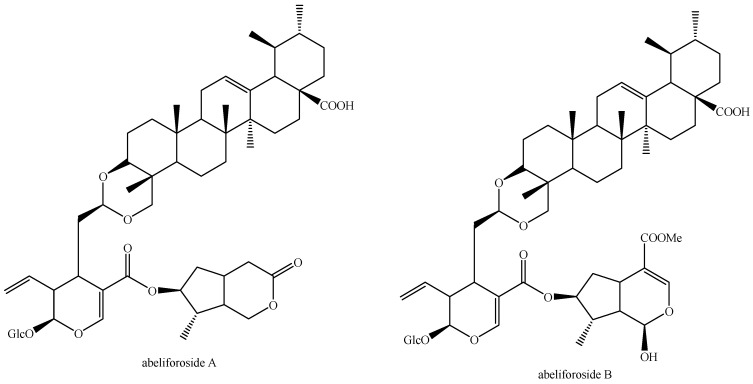
Structures of non-conventional *bis*-iridoids in plants—part 4.

**Table 1:** List of all the identified *bis*-iridoids in plants.

Name of the Compound	Plant Species	Studied Organ	Collection Area	Methodology of Extraction, Separation and Identification	Reference
5-hydroxy-2‴-*O*-caffeoyl-caryocanoside B (Figure 5)	*Caryopteris incana* (Thunb. ex Houtt.) Miq.	Whole plant	China	SE, PP, CC, α_[D]_, IR, NMR, HR-MS	[10]
7-O-acetyl-abelioside B (Figure 30)	*Linnaea chinensis* A.Braun & Vatke	Aerial parts	Italy	SE, PP, CC, α_[D]_, IR, UV, NMR, MS	[11]
7-O-acetyl-laciniatoside IV (Figure 30)	*Linnaea chinensis* A.Braun & Vatke	Aerial parts	Italy	SE, PP, CC, α_[D]_, IR, UV, NMR, MS	[11]
7-O-acetyl-laciniatoside V (Figure 30)	*Linnaea chinensis* A.Braun & Vatke	Aerial parts	Italy	SE, PP, CC, α_[D]_, IR, UV, NMR, MS	[11]
7-O-caffeoyl- sylvestroside I (Figure 9)	*Lomelosia stellata* (L.) Raf.	Whole plant	Algeria	SE, CC, CPC, rp-FC, HPLC-UV, α_[D]_, UV, NMR, HR-MS	[12]
7-O-(p-coumaroyl)-sylvestroside I (Figure 9)	*Lomelosia stellata* (L.) Raf.	Whole plant	Algeria	SE, CC, CPC, rp-FC, HPLC-UV, α_[D]_, UV, NMR, HR-MS	[12]
6′-O-(7α-hydroxy-swerosyloxy)-loganin (Figure 11)	*Lonicera japonica* Thunb.	Stems and leaves	Japan (purchased from a company)	SE, PP, VV, p-HPLC-UV, NMR	[13]
2‴-O-(E)-p-coumaroyl-caryocanoside B (Figure 5)	*Caryopteris incana* (Thunb. ex Houtt.) Miq	Whole plant	China	SE, PP, CC, p-HPLC-UV, α_[D]_, IR, NMR, HR-MS	[10]
2‴-O-(Z)-p-coumaroyl-caryocanoside B (Figure 5)	*Caryopteris incana* (Thunb. ex Houtt.) Miq.	Whole plant	China	SE, PP, CC, p-HPLC-UV, α_[D]_, IR, NMR, HR-MS	[10]
3″-glucosyl-depresteroside (Figure 10)	*Gentiana depressa* D.Don	Aerial parts	Nepal	DP, SE, PP, CC, CCTLC, sp-HPLC-UV, UV, NMR, MS	[14]
(Z)-aldosecologanin (Figure 17)	*Lonicera japonica* Thunb.	Stems and leaves	Japan (purchased from a company)	SE, PP, CC, p-HPLC-UV, α_[D]_, UV, NMR, HR-MS	[13]
		Flower buds	China	HSE, CC, p-HPLC-UV, NMR	[15]
			China (purchased from a company)	SE, PP, CC, sp-HPLC-UV, NMR	[16]
			China (different populations)	USE, HPLC-MS^n^	[17]
				SE, HPLC-PDA	[18]
			China (different populations)	USE, UHPLC-MS^n^	[19]
		Aerial parts	China (cultivated)	USE, UHPLC-MS^n^	[20]
		Roots	China (cultivated)	USE, UHPLC-MS^n^	[20]
		Flowers	China (different populations)	USE, UHPLC-MS^n^	[19]
		Stems	China (different populations)	USE, UHPLC-MS^n^	[19]
		Leaves	China (different populations)	USE, UHPLC-MS^n^	[19]
	*Lonicera ferdinandi* Franch.	Aerial parts	China (cultivated)	USE, UHPLC-MS^n^	[20]
		Roots	China (cultivated)	USE, UHPLC-MS^n^	[20]
	*Lonicera maximowiczii* subsp. *sachalinensis* (Fr.Schmidt) Nedol.	Aerial parts	China (cultivated)	USE, UHPLC-MS^n^	[20]
		Roots	China (cultivated)	USE, UHPLC-MS^n^	[20]
	*Lonicera maackii* (Rupr.) Maxim.	Aerial parts	China (cultivated)	USE, UHPLC-MS^n^	[20]
		Roots	China (cultivated)	USE, UHPLC-MS^n^	[20]
	*Lonicera morrowii* A.Gray	Aerial parts	China (cultivated)	USE, UHPLC-MS^n^	[20]
		Roots	China (cultivated)	USE, UHPLC-MS^n^	[20]
	*Lonicera praeflorens* Batalin	Aerial parts	China (cultivated)	USE, UHPLC-MS^n^	[20]
		Roots	China (cultivated)	USE, UHPLC-MS^n^	[20]
Abeliforoside A (Figure 35)	*Abelia grandiflora* (Rovelli ex André) Rehder	Flower buds	China	SE, PP, CC, sp-HPLC-UV, α_[D]_, IR, UV, NMR, HR-MS	[21]
Abeliforoside B (Figure 35)	*Abelia grandiflora* (Rovelli ex André) Rehder	Flower buds	China	SE, PP, CC, sp-HPLC-UV, α_[D]_, IR, UV, NMR, HR-MS	[21]
Abeliforoside C (Figure 30)	*Abelia grandiflora* (Rovelli ex André) Rehder	Flower buds	China	SE, PP, CC, sp-HPLC-UV, α_[D]_, IR, UV, NMR, HR-MS	[21]
Abeliforoside D (Figure 30)	*Abelia grandiflora* (Rovelli ex André) Rehder	Flower buds	China	SE, PP, CC, sp-HPLC-UV, α_[D]_, IR, UV, NMR, HR-MS	[21]
Abeliforoside E (Figure 30)	*Abelia grandiflora* (Rovelli ex André) Rehder	Flower buds	China	SE, PP, CC, sp-HPLC-UV, α_[D]_, IR, UV, NMR, HR-MS	[21]
Abeliforoside F (Figure 30)	*Abelia grandiflora* (Rovelli ex André) Rehder	Flower buds	China	SE, PP, CC, sp-HPLC-UV, α_[D]_, IR, UV, NMR, HR-MS	[21]
Abelioside A (Figure 30)	*Abelia grandiflora* (Rovelli ex André) Rehder	Leaves	Japan	HSE, PP, ACT, CC, p-TLC, α_[D]_, IR, UV, NMR	[22]
	*Picrorhiza kurroa* Royle ex Benth.	Stems	Myanmar	USE, PP, CC, sp-HPLC-UV, NMR	[23]
Abelioside A methyl acetal (Figure 30)	*Abelia grandiflora* (Rovelli ex André) Rehder	Leaves	Japan	HSE, PP, ACT, CC, p-TLC, α_[D]_, IR, UV, NMR	[22]
	*Pterocephalus hookeri* (C.B.Clarke) E.Pritz.	Whole plant	Tibet	SE, PP, CC, sp-HPLC-UV, NMR	[24]
Abelioside B (Figure 30)	*Picrorhiza kurroa* Royle ex Benth.	Stems	Myanmar	USE, PP, CC, sp-HPLC-UV, NMR	[23]
	*Abelia grandiflora* (Rovelli ex André) Rehder	Leaves	Japan	HSE, PP, ACT, CC, p-TLC, α_[D]_, IR, UV, NMR	[22]
Adinoside D (Figure 16)	*Adina racemosa* (Siebold & Zucc.) Miq.	Leaves, flowers and twigs	Taiwan (obtained from a botanical garden)	HSE, PP, CC, rp-MPLC, p-HPLC-UV, p-TLC, α_[D]_, IR, UV, NMR, HR-MS	[25]
Adinoside E (Figure 16)	*Adina racemosa* (Siebold & Zucc.) Miq.	Leaves, flowers and twigs	Taiwan (obtained from a botanical garden)	HSE, PP, CC, rp-MPLC, p-HPLC-UV, p-TLC, α_[D]_, IR, UV, NMR, HR-MS	[25]
Alatenoside (Figure 21)	*Sarracenia alata* (Alph.Wood) Alph.Wood	Whole plant	USA	SE, PP, p-rp-HPLC-UV, HPLC-ELSD, α_[D]_, UV, NMR, HR-MS	[26]
Alatinoside (Figure 21)	*Sarracenia alata* (Alph.Wood) Alph.Wood	Whole plant	USA	SE, PP, p-rp-HPLC-UV, HPLC-ELSD, α_[D]_, UV, NMR, HR-MS	[26]
Aldosecolohanin B (Figure 19)	*Lonicera japonica* Thunb.	Flower buds	China (purchased from a company)	SE, PP, CC, sp-HPLC-UV, α_[D]_, IR, UV, NMR, HR-MS	[16]
Aldosecolohanin C (Figure 19)	*Lonicera japonica* Thunb.	Flower buds	China (purchased from a company)	SE, PP, CC, sp-HPLC-UV, α_[D]_, IR, UV, NMR, HR-MS	[16]
Alidyjosioside (Figure 31)	*Scaevola taccada* (Gaertn.) Roxb.	Leaves	Egypt (obtained from a botanical garden)	SE, PP, VLC, CC, MP, NMR,	[27]
Arcusangeloside (Figure 34)	*Linaria arcusangeli* Atzei & Camarda	Whole plant	Italy	SE, ACT, CC, α_[D]_, IR, UV, NMR, MS	[28]
	*Linaria flava* subsp. *sardoa* (Sommier) Arrigoni	Whole plant	Italy	SE, ACT, CC, α_[D]_, IR, UV, NMR, MS	[28]
Argylioside (Figure 1)	*Argylia radiata* (L.) D.Don	Whole plant	Chile	SE, ACT, CC, rp-LPLC, α_[D]_, IR, UV, NMR	[29]
				SE, CC, NMR	[30]
Asaolaside (Figure 30)	*Loasa acerifolia* Dombey ex A.Juss.	Leaves	Germany (obtained from a botanical garden)	SXE, PP, CC, sp-HPLC-UV, α_[D]_, IR, UV, NMR, MS	[31]
Asperuloide A (Figure 29)	*Galium maximowiczii* (Kom.) Pobed.	Whole plant	South Korea	SE, PP, CC, p-HPLC-UV, α_[D]_, IR, UV, NMR, MS	[32]
Asperuloide B (Figure 29)	*Galium maximowiczii* (Kom.) Pobed.	Whole plant	South Korea	SE, PP, CC, p-HPLC-UV, α_[D]_, IR, UV, NMR, MS	[32]
Asperuloide C (Figure 34)	*Galium maximowiczii* (Kom.) Pobed.	Whole plant	South Korea	SE, PP, CC, p-HPLC-UV, α_[D]_, IR, UV, NMR, MS	[32]
Asperulosidyl-2’b-O-paederoside (Figure 4)	*Paederia foetida* L.	Aerial parts	China	SER, CC, sp-HPLC-UV, α_[D]_, IR, NMR, HR-MS	[33]
Atropurpurin A (Figure 9)	*Scabiosa atropurpurea* L.	Whole plant	Turkey	SE, CC, sp-HPLC-UV, HPLC-MS^n^, NMR	[34]
Atropurpurin B (Figure 9)	*Scabiosa atropurpurea* L.	Whole plant	Turkey	SE, CC, sp-HPLC-UV, HPLC-MS^n^, NMR	[34]
Austrosmoside (Figure 23)	*Osmanthus austrocaledonicus* (Vieill.) Knobl.	Aerial parts	New Caledonia	DP, CC, CC, VLC, α_[D]_, UV, NMR, HR-MS	[35]
Axillaroside (Figure 9)	*Strychnos axillaris* Colebr.	Bark and wood	Thailand	SER, PP, rp-MPLC, p-HPLC-UV, α_[D]_, IR, NMR, HR-MS	[36]
Blumeoside B (Figure 8)	*Fagraea blumei* G.Don	Stem bark	Indonesia	SE, CC, CPC, HPLC-DAD, α_[D]_, IR, NMR, MS	[37]
Blumeoside D (Figure 8)	*Fagraea blumei* G.Don	Stem bark	Indonesia	SE, CC, CPC, HPLC-DAD, α_[D]_, IR, NMR, MS	[37]
Caeruleoside A (Figure 11)	*Lonicera caerulea* L.	Leaves	Japan	SE, PP, CC, p-HPLC-UV, α_[D]_, IR, UV, NMR, MS	[38]
Caeruleoside B (Figure 18)	*Lonicera caerulea* L.	Leaves	Japan	SE, PP, CC, p-HPLC-UV, α_[D]_, IR, UV, NMR, MS	[38]
Cantleyoside (Figure 9)	*Cantleya corniculata* (Becc.) R.A.Howard	n.a.	n.a.	n.a.	[39]
	*Scabiosa japonica* Miq.	Roots	Japan	HSE, PP, CC, MP, α_[D]_, IR, UV, NMR	[40]
	*Dipsacus fullonum* L.	Seeds	Denmark	SE, p-TLC, α_[D]_, UV, NMR	[41]
		Leaves	Poland	USE, UHPLC-PDA-MS^n^	[42]
		Roots	Poland	USE, UHPLC-PDA-MS^n^	[42]
	*Abelia grandiflora* (Rovelli ex André) Rehder	Leaves	Japan	HSE, PP, ACT, CC, p-TLC, PLC, NMR	[22]
	*Linnaea spathulata* Graebn.	Leaves	Japan	SE, ACT, p-TLC, NMR	[22]
	*Linnaea serrata* Graebn.	Leaves	Japan	SE, ACT, p-TLC, NMR	[22]
	*Scaevola montana Labill.*	Aerial parts	New Caledonia	SE, CC, NMR	[43]
	*Scaevola racemigera* Däniker	Aerial parts	New Caledonia	SE, CC, NMR	[44]
	*Dipsacus laciniatus* L.	Roots	Hungary	SE, PP, CCD, CC, α_[D]_, IR, UV, NMR	[45]
	*Cephalaria ambrosioides* (Sm.) Roem. & Schult.	Roots	Greece	SE, PP, CC, rp-CC, NMR	[46]
	*Lomelosia variifolia* (Boiss.) Greuter & Burdet	Flowering aerial parts	Greece	SE, VLC, rp-MPLC, NMR, MS	[47]
	*Dipsacus inermis* Wall.	Roots	China	HSE, PP, CC, rp-CC, p-TLC, rp-HPLC-UV, NMR	[48]
				SER, PP, CC, NMR	[49]
			China (purchased from a company)	SER, PP, MPLC, p-TLC, NMR	[50]
		Dried Roots	China (purchased from a company)	USE, HPLC-MS^n^	[51]
			China (different populations)	SE, CC, UHPLC-PDA, UHPLC-MS^n^	[52]
	*Strychnos spinosa* Lam.	Branches	Japan (cultivated)	HSE, PP, rp-MPLC, p-HPLC-UV, p-TLC, NMR	[53]
	*Strychnos lucida* R.Br.	Bark and wood	Thailand	HSE, PP, MPLC, rp-MPLC, p-HPLC-UV, NMR	[54]
	*Strychnos axillaris* Colebr.	Bark and wood	Thailand	SER, PP, rp-MPLC, p-HPLC-UV, NMR	[36]
	*Pterocephalus pinardi* Boiss.	Aerial parts	Turkey	SE, PP, rp-VLC, CC, MPLC, NMR	[55]
	*Cephalaria kotschyi* Boiss. & Hohen.	Dried roots	Azerbaijan	SE, FC, LPLC, NMR	[56]
	*Cephalaria media* Litv.	Dried roots	Azerbaijan	SE, CC, rp-CC, TLC, NMR	[57]
	*Pterocephalus hookeri* (C.B.Clarke) E.Pritz.	Underground parts	Tibet	SER, PP, CC rp-CC, NMR	[58]
				SER, PP, TLC, sp-HPLC-MS, NMR	[59]
		n.a.	n.a.	n.a.	[60]
		Whole plant	China	SE, PP, CC, rp-CC, NMR	[61]
				SE, PP, HPLC-UV	[62]
				SER, CC, UPLC-PDA	[63]
				USE, UPLC-MS^n^	[64]
			Tibet	SE, PP, CC, p-HPLC-UV, p-TLC, NMR	[65]
			Tibet	SE, PP, CC, sp-HPLC-UV, NMR	[24]
			China (different populations)	USE, UPLC-MS^n^	[66]
	*Pterocephalus nestorianus* Nábelek	Roots	Iraq	DP, SE, PP, MPLC, p-TLC, NMR	[67]
	*Scabiosa atropurpurea* L.	Roots	Turkey	HSE, rp-CC, CC, NMR, MS	[68]
		Whole plant		SE, CC, sp-HPLC-UV, HPLC-MS^n^	[34]
		Leaves	Tunisia	SE, DP, HPLC-MS^n^	[69]
Cantleyoside dimethyl acetal (Figure 9)	*Scaevola montana Labill.*	Aerial parts	New Caledonia	SE, CC, NMR	[43]
	*Pterocephalus pterocephalus* (L.) Dörfl.	Aerial parts	Greece	SE, CC, rp-CC, α_[D]_, NMR, MS	[70]
	*Pterocephalus pinardi* Boiss.	Aerial parts	Turkey	SE, PP, rp-VLC, CC, MPLC, NMR	[55]
	*Scabiosa atropurpurea* L.	Whole plant	Turkey	SE, CC, sp-HPLC-UV, HPLC-MS^n^, NMR	[34]
Caryocanoside B (Figure 5)	*Caryopteris incana* (Thunb. ex Houtt.) Miq.	Whole plant	China	SE, PP, CC, p-TLC, α_[D]_, IR, NMR, HR-MS	[10]
Centauroside (Figure 21)	*Centaurium erythraea* Rafn	n.a.	n.a.	n.a.	[71]
	*Lonicera japonica* Thunb.	Stems and leaves	Japan (purchased from a company)	SE, PP, VV, p-HPLC-UV, α_[D]_, UV, NMR, HR-MS	[13]
		Dried flowers	South Korea (different populations)	USE, HPLC-UV	[72]
			South Korea (different commercial samples)	USE, HPLC-UV	
		Caulis	China (different populations)	USE, UFLC-MS^n^	[73]
			China (samples purchased from different companies)	USE, UFLC-MS^n^	
		Flowers	China (different populations)	USE, UFLC-MS^n^	[73]
			China (samples purchased from different companies)	USE, UFLC-MS^n^	
			China (different populations)	USE, UHPLC-MS^n^	[19]
		Flower buds	China	SER, HPLC-MS^n^	[74]
			China	DP, SER, HPLC-DAD-MS^n^	[75]
				SER, HPLC-MS	[76]
			China (different cultivated populations)	USE, HPLC-DAD-ELSD	[77]
			China (commercial samples)	USE, HPLC-DAD-ELSD	[77]
				SE, HPLC-DAD, HPLC-MS	[78]
			China and Korea (commercial samples)	SE, HPLC-DAD-MS	[79]
			China	n.a.	[80]
				HSE, CC, p-HPLC-UV, NMR	[15]
				USE, HPLC-DAD-CL, HPLC-DAD-MS^n^	[81]
			China (different populations)	USE, HPLC-MS^n^	[17]
				HSE, UHPLC-UV	[82]
				USE, UFLC-MS^n^	[73]
				SE, HPLC-PDA	[18]
				USE, UHPLC-MS^n^	[19]
			China (purchased from a company)	USE, rp-UHPLC-PDA-MS^n^	[83]
				USE, 2D-HPLC-UF-MS	[84]
				SE, PP, CC, sp-HPLC-UV, NMR	[16]
			China (samples purchased from different companies)	USE, UFLC-MS^n^	[73]
			China (cultivated)	USE, UPLC-MS^n^	[85]
		Leaves	South Korea (different populations)	USE, HPLC-UV	[72]
			China (purchased from a company)	USE, HPLC-DAD-MS^n^	[86]
			China	USE, rp-UHPLC-PDA-MS^n^	[83]
			China (different populations)	USE, UFLC-MS^n^	[73]
				USE, UHPLC-MS^n^	[19]
			China (cultivated)	USE, UPLC-MS^n^	[85]
		Aerial parts	China (cultivated)	USE, UHPLC-MS^n^	[20]
		Roots	China (cultivated)	USE, UHPLC-MS^n^	[20]
		Stems	China	USE, rp-UHPLC-PDA-MS^n^	[83]
			China (different populations)	USE, UHPLC-MS^n^	[19]
		Branches	China (cultivated)	USE, UPLC-MS^n^	[85]
		Fruits	China (cultivated)	USE, UPLC-MS^n^	[85]
	*Kissenia capensis* Endl.	Aerial parts	Namibia	SE, PP, CC, rp-CC, sp-rp-HPLC-UV, NMR, MS	[87]
	*Strychnos spinosa* Lam.	Branches	Japan (cultivated)	HSE, PP, rp-MPLC, p-HPLC-UV, p-TLC, NMR	[53]
	*Lonicera confusa* DC.	Flower buds	China (different cultivated populations)	USE, HPLC-DAD-ELSD	[77]
			China	DP, SER, HPLC-DAD-MS^n^	[75]
			China (different populations)	SER, HPLC-MS	[76]
		Dried flowers	South Korea (different populations)	USE, HPLC-UV	[72]
			South Korea (different commercial samples)	USE, HPLC-UV	[72]
	*Lonicera ferdinandi* Franch.	Aerial parts	China (cultivated)	USE, UHPLC-MS^n^	[20]
		Roots	China (cultivated)	USE, UHPLC-MS^n^	[20]
	*Lonicera hypoglauca Miq.*	Flower buds	China (different cultivated populations)	USE, HPLC-DAD-ELSD	[77]
			China	DP, SER, HPLC-DAD-MS^n^	[75]
				SER, HPLC-MS	[76]
	*Lonicera macrantha* Spreng.	Flower buds	China (different cultivated populations)	USE, HPLC-DAD-ELSD	[77]
			China (different populations)	DP, SER, HPLC-DAD-MS^n^	[75]
				SER, HPLC-MS	[76]
				HSE, UHPLC-UV	[82]
	*Lonicera maackii* (Rupr.) Maxim.	Aerial parts	China (cultivated)	USE, UHPLC-MS^n^	[20]
		Roots	China (cultivated)	USE, UHPLC-MS^n^	[20]
	*Lonicera maximowiczii* subsp. *sachalinensis* (Fr.Schmidt) Nedol.	Aerial parts	China (cultivated)	USE, UHPLC-MS^n^	[20]
		Roots	China (cultivated)	USE, UHPLC-MS^n^	[20]
	*Lonicera praeflorens* Batalin	Aerial parts	China (cultivated)	USE, UHPLC-MS^n^	[20]
		Roots	China (cultivated)	USE, UHPLC-MS^n^	[20]
	*Lonicera rupicola* var. *syringantha* (Maxim.) Zabel	Flower buds	China	SER, HPLC-MS	[76]
	*Lonicera similis* Hemsl. ex F.B.Forbes & Hemsl.	Flower buds	China	SER, HPLC-MS	[76]
	*Triosteum pinnatifidum* Maxim.	Roots	China	SER, PP, CC, NMR	[88]
	*Gentianella amarella* subsp. *acuta* (Michx.) J.M.Gillett	Whole plant	China	SER, PP, CC, p-HPLC-UV, NMR	[89]
	*Lonicera morrowii* A.Gray	Roots	South Korea (obtained from a botanical garden)	USE, PP, CC, p-HPLC-UV, NMR	[20]
		Aerial parts	China (cultivated)	USE, UHPLC-MS^n^	[20]
		Roots	China (cultivated)	USE, UHPLC-MS^n^	[20]
Centauroside A (Figure 21)	*Centaurium erythraea* Rafn	Whole plant	Turkey	SE, CC, rp-FC, α_[D]_, IR, UV, NMR, HR-MS	[90]
Chrysathain (Figure 22)	*Lonicera chrysantha* Turcz. ex Ledeb.	Leaves	China	SE, CC, α_[D]_, NMR, HR-MS	[91]
Citrifolinin A-1 (Figure 6)	*Morinda citrifolia* L.	Leaves	India	HSE, PP, CC, rp-CC, NMR, MS	[92]
Cocculoside (Figure 9)	*Strychnos cocculoides* Baker	Stem bark	Tanzania	SE, VLC, CC, α_[D]_, IR, UV, NMR, MS	[93]
	*Dipsacus inermis* Wall.	Roots	China (purchased from a local market)	SE, PP, CC, rp-CC, sp-HPLC-UV, NMR	[94]
Coelobillardin (Figure 8)	*Coelospermum balansanum* Baill.	Aerial parts	New Caledonia	SE, CC, MPLC, α_[D]_, IR, UV, NMR, HR-MS	[95]
Coptosapside A (Figure 31)	*Coptosapelta diffusa* (Champ. ex Benth.) Steenis	Aerial parts	China	SE, PP, MPLC, CC, α_[D]_, IR, UV, NMR, HR-MS	[96]
Coptosapside D (Figure 14)	*Coptosapelta diffusa* (Champ. ex Benth.) Steenis	Aerial parts	China	SE, PP, MPLC, CC, α_[D]_, IR, UV, NMR, HR-MS	[96]
Coptosapside E (Figure 14)	*Coptosapelta diffusa* (Champ. ex Benth.) Steenis	Aerial parts	China	SE, PP, MPLC, CC, α_[D]_, IR, UV, NMR, HR-MS	[96]
Coptosapside F (Figure 14)	*Coptosapelta diffusa* (Champ. ex Benth.) Steenis	Aerial parts	China	SE, PP, MPLC, CC, α_[D]_, IR, UV, NMR, HR-MS	[96]
Cornuofficinaliside C (Figure 13)	*Cornus officinalis* Siebold & Zucc.	Fruits	China	SE, CC, PP, sp-HPLC-UV, α_[D]_, IR, UV, NMR, HR-MS	[97]
Cornuofficinaliside D (Figure 13)	*Cornus officinalis* Siebold & Zucc.	Fruits	China	SE, CC, PP, sp-HPLC-UV, α_[D]_, IR, UV, NMR, HR-MS	[97]
Cornuofficinaliside E (Figure 13)	*Cornus officinalis* Siebold & Zucc.	Fruits	China	SE, CC, PP, sp-HPLC-UV, α_[D]_, IR, UV, NMR, HR-MS	[97]
Cornuofficinaliside F (Figure 13)	*Cornus officinalis* Siebold & Zucc.	Fruits	China	SE, CC, PP, sp-HPLC-UV, α_[D]_, IR, UV, NMR, HR-MS	[97]
Cornuofficinaliside G (Figure 13)	*Cornus officinalis* Siebold & Zucc.	Fruits	China	SE, CC, PP, sp-HPLC-UV, α_[D]_, IR, UV, NMR, HR-MS	[97]
Cornuofficinaliside H (Figure 13)	*Cornus officinalis* Siebold & Zucc.	Fruits	China	SE, CC, PP, sp-HPLC-UV, α_[D]_, IR, UV, NMR, HR-MS	[97]
Cornuofficinaliside I (Figure 13)	*Cornus officinalis* Siebold & Zucc.	Fruits	China	SE, CC, PP, sp-HPLC-UV, α_[D]_, IR, UV, NMR, HR-MS	[97]
Cornuofficinaliside J (Figure 26)	*Cornus officinalis* Siebold & Zucc.	Fruits	China	SE, CC, PP, sp-HPLC-UV, α_[D]_, IR, UV, NMR, HR-MS	[97]
Cornuofficinaliside K (Figure 26)	*Cornus officinalis* Siebold & Zucc.	Fruits	China	SE, CC, PP, sp-HPLC-UV, α_[D]_, IR, UV, NMR, HR-MS	[97]
Cornuofficinaliside L (Figure 26)	*Cornus officinalis* Siebold & Zucc.	Fruits	China	SE, CC, PP, sp-HPLC-UV, α_[D]_, IR, UV, NMR, HR-MS	[97]
Cornuofficinaliside M (Figure 26)	*Cornus officinalis* Siebold & Zucc.	Fruits	China	SE, CC, PP, sp-HPLC-UV, α_[D]_, IR, UV, NMR, HR-MS	[97]
Cornusdiridoid A (Figure 25)	*Cornus officinalis* Siebold & Zucc.	Fruits	China	HSE, CC, sp-HPLC-UV, α_[D]_, IR, UV, NMR, HR-MS	[98]
			China (purchased from a local market)	SER, PP, CC, sp-HPLC-UV, NMR	[99]
Cornusdiridoid B (Figure 25)	*Cornus officinalis* Siebold & Zucc.	Fruits	China	HSE, CC, sp-HPLC-UV, α_[D]_, IR, UV, NMR, HR-MS	[98]
Cornusdiridoid C (Figure 25)	*Cornus officinalis* Siebold & Zucc.	Fruits	China	HSE, CC, sp-HPLC-UV, α_[D]_, IR, UV, NMR, HR-MS	[98]
Cornusdiridoid D (Figure 25)	*Cornus officinalis* Siebold & Zucc.	Fruits	China	HSE, CC, sp-HPLC-UV, α_[D]_, IR, UV, NMR, HR-MS	[98]
Cornusdiridoid E (Figure 26)	*Cornus officinalis* Siebold & Zucc.	Fruits	China	HSE, CC, sp-HPLC-UV, α_[D]_, IR, UV, NMR, HR-MS	[98]
Cornusdiridoid F (Figure 26)	*Cornus officinalis* Siebold & Zucc.	Fruits	China	HSE, CC, sp-HPLC-UV, α_[D]_, IR, UV, NMR, HR-MS	[98]
Cornuside A (Figure 24)	*Cornus officinalis* Siebold & Zucc.	Fruits	China	SER, CC, p-HPLC-UV, α_[D]_, IR, UV, NMR, HR-MS	[99]
			China (purchased from a local market)	SER, PP, CC, sp-HPLC-UV, NMR	[100]
			China (different populations purchased from a company)	HSE, UHPLC-MS^n^	[101]
Cornuside B (Figure 24)	*Cornus officinalis* Siebold & Zucc.	Fruits	China	SER, CC, p-HPLC-UV, α_[D]_, IR, UV, NMR, HR-MS	[99]
Cornuside C (Figure 24)	*Cornus officinalis* Siebold & Zucc.	Fruits	China	SER, CC, p-HPLC-UV, α_[D]_, IR, UV, NMR, HR-MS	[99]
Cornuside D (Figure 24)	*Cornus officinalis* Siebold & Zucc.	Fruits	China	SER, CC, p-HPLC-UV, α_[D]_, IR, UV, NMR, HR-MS	[99]
Cornuside E (Figure 24)	*Cornus officinalis* Siebold & Zucc.	Fruits	China	SER, CC, p-HPLC-UV, α_[D]_, IR, UV, NMR, HR-MS	[99]
			China (purchased from a local market)	SER, PP, CC, sp-HPLC-UV, NMR	[100]
			China (different populations purchased from a company)	HSE, UHPLC-MS^n^	[101]
Cornuside F (Figure 24)	*Cornus officinalis* Siebold & Zucc.	Fruits	China	SER, CC, p-HPLC-UV, α_[D]_, IR, UV, NMR, HR-MS	[99]
Cornuside G (Figure 24)	*Cornus officinalis* Siebold & Zucc.	Fruits	China	SER, CC, p-HPLC-UV, α_[D]_, IR, UV, NMR, HR-MS	[99]
Cornuside H (Figure 24)	*Cornus officinalis* Siebold & Zucc.	Fruits	China	SER, CC, p-HPLC-UV, α_[D]_, IR, UV, NMR, HR-MS	[99]
Cornuside I (Figure 24)	*Cornus officinalis* Siebold & Zucc.	Fruits	China	SER, CC, p-HPLC-UV, α_[D]_, IR, UV, NMR, HR-MS	[99]
Cornuside J (Figure 24)	*Cornus officinalis* Siebold & Zucc.	Fruits	China	SER, CC, p-HPLC-UV, α_[D]_, IR, UV, NMR, HR-MS	[99]
Cornuside K (Figure 24)	*Cornus officinalis* Siebold & Zucc.	Fruits	China	SER, CC, p-HPLC-UV, α_[D]_, IR, UV, NMR, HR-MS	[99]
			China (purchased from a local market)	SER, PP, CC, sp-HPLC-UV, NMR	[100]
Cornuside L (Figure 12)	*Cornus officinalis* Siebold & Zucc.	Fruits	China	SER, CC, p-HPLC-UV, α_[D]_, IR, UV, NMR, HR-MS	[99]
			China (different populations purchased from a company)	HSE, UHPLC-MS^n^	[101]
Cornuside M (Figure 12)	*Cornus officinalis* Siebold & Zucc.	Fruits	China	SER, CC, p-HPLC-UV, α_[D]_, IR, UV, NMR, HR-MS	[99]
			China (different populations purchased from a company)	HSE, UHPLC-MS^n^	[101]
Cornuside N (Figure 12)	*Cornus officinalis* Siebold & Zucc.	Fruits	China	SER, CC, p-HPLC-UV, α_[D]_, IR, UV, NMR, HR-MS	[99]
			China (different populations purchased from a company)	HSE, UHPLC-MS^n^	[101]
Cornuside O (Figure 12)	*Cornus officinalis* Siebold & Zucc.	Fruits	China	SER, CC, p-HPLC-UV, α_[D]_, IR, UV, NMR, HR-MS	[99]
			China	SE, CC, PP, sp-HPLC-UV, α_[D]_, IR, UV, NMR, HR-MS	[97]
Craigoside B (Figure 22)	*Jasminum abyssinicum* Hochst. ex DC.	Root bark	Congo	SE, PP, CCD, α_[D]_, UV, CD, NMR, HR-MS	[102]
Craigoside C (Figure 22)	*Jasminum abyssinicum* Hochst. ex DC.	Root bark	Congo	SE, PP, CCD, α_[D]_, UV, CD, NMR, HR-MS	[102]
Demethyl-hydroxy-oleonuezhenide	*Syringa vulgaris* L.	Flowers	Poland	HSE, CC, p-HPLC-UV, α_[D]_, UV, NMR, HR-MS	[103]
Demethyl-oleonuezhenide	*Syringa vulgaris* L.	Flowers	Poland	HSE, CC, p-HPLC-UV, α_[D]_, UV, NMR, HR-MS	[103]
Depresteroside (Figure 10)	*Gentiana depressa* D.Don	Aerial parts	Nepal	DP, SE, PP, CC, CCTLC, UV, NMR, MS^n^	[104]
Dioscoridin C (Figure 5)	*Valeriana italica* Lam.	Roots	Turkey	HSE, PP, CC, MPLC, α_[D]_, IR, UV, NMR, HR-MS	[105]
Dipsanoside C (Figure 10)	*Dipsacus inermis* Wall.	Dried roots	China	HSE, PP, CC, rp-CC, p-TLC, rp-HPLC-UV, α_[D]_, IR, UV, NMR, HR-MS	[48]
Dipsanoside D (Figure 10)	*Dipsacus inermis* Wall.	Dried roots	China	HSE, PP, CC, rp-CC, p-TLC, rp-HPLC-UV, α_[D]_, IR, UV, NMR, HR-MS	[48]
Dipsanoside E (Figure 10)	*Dipsacus inermis* Wall.	Dried roots	China	HSE, PP, CC, rp-CC, p-TLC, rp-HPLC-UV, α_[D]_, IR, UV, NMR, HR-MS	[48]
Dipsanoside F (Figure 11)	*Dipsacus inermis* Wall.	Dried roots	China	HSE, PP, CC, rp-CC, p-TLC, rp-HPLC-UV, α_[D]_, IR, UV, NMR, HR-MS	[48]
Dipsanoside G (Figure 31)	*Dipsacus inermis* Wall.	Dried roots	China	HSE, PP, CC, rp-CC, p-TLC, rp-HPLC-UV, α_[D]_, IR, UV, NMR, HR-MS	[48]
Dipsanoside J (Figure 10)	*Dipsacus inermis* Wall.	Dried roots	China	HSE, PP, CC, p-TLC, p-rp-HPLC-UV, α_[D]_, IR, NMR, HR-MS	[106]
Dipsanoside M (Figure 11)	*Dipsacus inermis* Wall.	Dried roots	China	SER, CC, rp-CC, rp-FC, p-HPLC-UV, α_[D]_, IR, UV, NMR, HR-MS	[107]
Dipsanoside N (Figure 11)	*Dipsacus inermis* Wall.	Dried roots	China	SER, CC, rp-CC, rp-FC, p-HPLC-UV, α_[D]_, IR, UV, NMR, HR-MS	[107]
Dipsaperine (Figure 11)	*Dipsacus inermis* Wall.	Roots	China (purchased from a local market)	SE, PP, CC, rp-CC, sp-HPLC-UV, α_[D]_, IR, UV, ECD, NMR, HR-MS	[94]
				SER, PP, MPLC, p-HPLC-UV, α_[D]_, IR, UV, NMR, HR-MS	[108]
Disperoside A (Figure 7)	*Gardenia jasminoides* J.Ellis	Fruits	China	SE, PP, CC, sp-HPLC-UV, α_[D]_, IR, UV, NMR, HR-MS	[109]
Disperoside B (Figure 7)	*Gardenia jasminoides* J.Ellis	Fruits	China	SE, PP, CC, sp-HPLC-UV, α_[D]_, IR, UV, NMR, HR-MS	[109]
Floribundal (Figure 28)	*Scaevola floribunda* A.Gray	Heartwood	Japan	SXE, PP, VLC, MP, α_[D]_, IR, UV, NMR, MS	[110]
Fraximalacoside (Figure 18)	*Fraxinus malacophylla* Hemsl.	Leaves	China (obtained from a botanical garden)	HSE, PP, CC, HPLC-UV, α_[D]_, IR, UV, NMR, MS	[111]
	*Fraxinus mandshurica* Rupr.	Whole plant	China (different populations)	USE, HPLC-DAD, UPLC-MS	[112]
GI-3 (Figure 17)	*Fraxinus americana* L.	Seeds	USA	SE, PP, CC, MP, α_[D]_, TLC	[113]
		Leaves		SE, CC, TLC, IR, UV, NMR	[114]
	*Fraxinus excelsior* L.	Seeds	USA	SE, PP, CC, MP, α_[D]_, TLC	[113]
			Morocco	HSE, PP, CC, HPLC-UV, NMR	[115]
	*Fraxinus ornus* L.	Seeds	USA	SE, PP, CC, MP, α_[D]_, TLC	[113]
	*Fraxinus pennsylvanica* Marshall	Seeds	USA	SE, PP, CC, MP, α_[D]_, TLC	[113]
	*Olea europaea* L.	Seeds	USA	SE, PP, CC, MP, α_[D]_, TLC	[113]
	*Syringa vulgaris* L.	Seeds	USA	SE, PP, CC, MP, α_[D]_, TLC	[113]
	*Ligustrum lucidum* W.T.Aiton	Dried fruits	China	SE, PP, CC, p-HPLC-UV, NMR	[116]
				SER, PP, CC, NMR	[117]
				USE, UHPLC-MS^n^	[118]
		Fruits		SER, PP, CC, p-HPLC-UV, MP, α_[D]_, IR, UV, NMR, HR-MS	[119]
	*Osmanthus fragrans* Lour.	Seeds	China	SE, PP, CC, NMR	[120]
	*Ligustrum japonicum* Thunb.	Fruits	South Korea	SER, PP, CC, α_[D]_, IR, UV, NMR, HR-MS	[121]
		Dried fruits	South Korea	SE, PP, CC, rp-HPLC-UV, NMR, MS	[122]
	*Fraxinus mandshurica* Rupr.	Seeds	China (purchased from a company)	SE, PP, CC, HPLC-DAD, NMR	[123]
GI-5 (Figure 17)	*Fraxinus americana* L.	Seeds	USA	SE, PP, CC, MP, α_[D]_, TLC	[113]
		Leaves		SE, CC, TLC, IR, UV, NMR	[114]
	*Fraxinus excelsior* L.	Seeds	USA	SE, PP, CC, MP, α_[D]_, TLC	[113]
			Morocco	HSE, PP, CC, HPLC-UV, NMR	[115]
	*Fraxinus ornus* L.	Seeds	USA	SE, PP, CC, MP, α_[D]_, TLC	[113]
	*Fraxinus pennsylvanica* Marshall	Seeds	USA	SE, PP, CC, MP, α_[D]_, TLC	[113]
	*Olea europaea* L.	Seeds	USA	SE, PP, CC, MP, α_[D]_, TLC	[113]
	*Syringa vulgaris* L.	Seeds	USA	SE, PP, CC, MP, α_[D]_, TLC	[113]
	*Jasminum polyanthum* Franch.	Flowers	China (purcahsed from a company)	HSE, PP, CC, p-HPLC, α_[D]_, IR, UV, NMR, HR-MS	[124]
	*Fraxinus mandshurica* Rupr.	Seeds	China (purchased from a company)	SE, PP, CC, HPLC-DAD, NMR	[123]
Globuloside A (Figure 7)	*Globularia trichosantha* Fisch. & C.A.Mey.	Underground parts	Turkey	HSE, PP, rp-VLC, CC, MPLC, α_[D]_, IR, NMR, MS	[125]
	*Globularia meridionalis* (Podp.) O.Schwarz	Aerial parts	Italy	SE, PP, CC, NMR	[126]
	*Globularia alypum* L.	Aerial parts	Croatia	SER, HPLC-PDA, HPLC-PDA-MS^n^	[127]
		Leaves	Croatia	USE, HPLC-PDA-MS^n^	[128]
				SXE, HPLC-PDA-MS^n^	
Globuloside B (Figure 6)	*Globularia trichosantha* Fisch. & C.A.Mey.	Underground parts	Turkey	HSE, PP, rp-VLC, CC, MPLC, α_[D]_, IR, UV, NMR, MS	[125]
	*Globularia meridionalis* (Podp.) O.Schwarz	Aerial parts	Italy	SE, PP, CC, NMR	[126]
Globuloside C (Figure 11)	*Globularia cordifolia* L.	Roots and rhizomes	Turkey	HSE, PP, VLC, MPLC, CC, α_[D]_, IR, UV, NMR, HR-MS	[129]
Hookerinoid A (Figure 28)	*Pterocephalus hookeri* (C.B.Clarke) E.Pritz.	Underground parts	China	SER, PP, CC, sp-HPLC-UV, α_[D]_, IR, UV, NMR, HR-MS	[130]
Hookerinoid B (Figure 28)	*Pterocephalus hookeri* (C.B.Clarke) E.Pritz.	Underground parts	China	SER, PP, CC, sp-HPLC-UV, α_[D]_, IR, UV, NMR, HR-MS	[130]
Hydroxy-oleonuezhenide	*Syringa vulgaris* L.	Flowers	Poland	HSE, CC, p-HPLC-UV, α_[D]_, UV, NMR, HR-MS	[103]
Ilicifolioside A (Figure 19)	*Osmanthus heterophyllus* (G.Don) P.S.Green	Leaves	Japan	SE, PP, CC, p-HPLC-UV, α_[D]_, UV, NMR, HR-MS	[131]
Ilicifolioside B (Figure 22)	*Osmanthus heterophyllus* (G.Don) P.S.Green	Leaves	Japan	SE, PP, CC, p-HPLC-UV, α_[D]_, UV, NMR, HR-MS	[131]
Incaside (Figure 29)	*Mussaenda incana* Wall.	Stem bark	n.a.	n.a.	[132]
Iridolinarin A (Figure 29)	*Linaria japonica* Miq.	Whole plant	Japan	SE, PP, CC, α_[D]_, IR, UV, NMR, HR-MS	[133]
Iridolinarin B (Figure 33)	*Linaria japonica* Miq.	Whole plant	Japan	SE, PP, CC, α_[D]_, IR, UV, NMR, HR-MS	[133]
Iridolinarin C (Figure 29)	*Linaria japonica* Miq.	Whole plant	Japan	SE, PP, CC, α_[D]_, IR, UV, NMR, HR-MS	[133]
Iso-jaspolyoside A (Figure 17)	*Jasminum polyanthum* Franch.	Flowers	China (purcahsed from a company)	HSE, PP, CC, p-TLC, p-HPLC-UV, α_[D]_, IR, UV, NMR, HR-MS	[134]
	*Olea europaea* L.	Wood	Spain	SER, CC, rp-HPLC-DAD, NMR	[135]
			Spain (different populations)	SE, HPLC-DAD, HPLC-DAD-MS	[136]
Iso-jaspolyoside B (Figure 18)	*Jasminum polyanthum* Franch.	Flowers	China (purcahsed from a company)	HSE, PP, CC, p-TLC, p-HPLC-UV, α_[D]_, IR, UV, NMR, HR-MS	[134]
Iso-jaspolyoside C (Figure 18)	*Jasminum polyanthum* Franch.	Flowers	China (purcahsed from a company)	HSE, PP, CC, p-TLC, p-HPLC-UV, α_[D]_, IR, UV, NMR, HR-MS	[134]
Iso-oleonuzhenide (Figure 15)	*Ligustrum lucidum* W.T.Aiton	Dried fruits	China	SE, PP, CC, p-HPLC-UV, α_[D]_, IR, UV, NMR, HR-MS	[116]
	*Ligustrum japonicum* Thunb.	Fruits	South Korea	SER, PP, CC, rp-CC, α_[D]_, IR, UV, NMR, HR-MS	[121]
	*Fraxinus mandshurica* Rupr.	Seeds	China (purchased from a company)	SE, PP, CC, HPLC-DAD, NMR	[123]
Japonicoside E (Figure 33)	*Lonicera japonica* Thunb.	Flower buds	China (purchased from a company)	SER, CC, p-HPLC-UV, sp-HPLC-UV, α_[D]_, IR, UV, NMR, HR-MS	[137]
Jasmigeniposide B (Figure 1)	*Gardenia jasminoides* J.Ellis	Fruits	China (purchased from a company)	SER, PP, CC, rp-HPLC-UV, α_[D]_, IR, UV, NMR, HR-MS	[138]
Jasnervoside F (Figure 20)	*Jasminum nervosum* Lour.	Stems	China (purchased from a local market)	SER, PP, CC, α_[D]_, IR, UV, NMR, HR-MS	[139]
Jasnudifloside D (Figure 14)	*Jasminum nudiflorum* Lindl.	Stems	Japan (obtained from a botanical garden)	HSE, PP, CC, p-HPLC-UV, α_[D]_, IR, UV, NMR, HR-MS	[140]
Jasnudifloside E (Figure 14)	*Jasminum nudiflorum* Lindl.	Stems	Japan (obtained from a botanical garden)	HSE, PP, CC, p-HPLC-UV, α_[D]_, IR, UV, NMR, HR-MS	[140]
Jasnudifloside H (Figure 14)	*Jasminum nudiflorum* Lindl.	Leaves	Japan (obtained from a botanical garden)	HSE, PP, CC, p-TLC, p-HPLC-UV, α_[D]_, IR, UV, NMR, HR-MS	[141]
Jasnudifloside L (Figure 14)	*Jasminum nudiflorum* Lindl.	Leaves	Japan (obtained from a botanical garden)	HSE, PP, CC, p-TLC, p-HPLC-UV, α_[D]_, IR, UV, NMR, HR-MS	[141]
Jaspolyanoside (Figure 23)	*Jasminum polyanthum* Franch.	Flowers	China (purcahsed from a company)	HSE, PP, CC, p-TLC, p-HPLC-UV, α_[D]_, IR, UV, NMR, HR-MS	[134]
	*Olea europaea* L.	Wood	Spain	SER, CC, rp-HPLC-DAD, NMR	[135]
			Spain (different populations)	SE, HPLC-DAD, HPLC-DAD-MS	[136]
	*Syringa oblata* subsp. *dilatata* (Nakai) P.S.Green & M.C.Chang	Twigs	South Korea	SE, PP, CC, rp-CC, rp-HPLC-UV, NMR	[142]
	*Fraxinus mandshurica* Rupr.	Seeds	China (purchased from a company)	SE, PP, CC, HPLC-DAD, NMR	[123]
Jaspolyanthoside (Figure 22)	*Jasminum polyanthum* Franch.	Flowers	China (purcahsed from a company)	HSE, PP, CC, p-HPLC-UV, α_[D]_, IR, UV, NMR, HR-MS	[124]
	*Jasminum nervosum* Lour.	Stems	China (purchased from a local market)	SER, PP, CC, α_[D]_, IR, UV, NMR, HR-MS	[139]
	*Jasminum grandiflorum* subsp. *floribundum* (R.Br. ex Fresen.) P.S.Green	Aerial parts	Saudi Arabia	USE, PP, HPLC-DAD, UPLC-HR-MS	[143]
Jaspolyoside (Figure 23)	*Jasminum polyanthum* Franch.	Flowers	China (purchased from a company)	HSE, PP, CC, p-HPLC, α_[D]_, IR, UV, NMR, HR-MS	[124]
	*Syringa reticulata* (Blume) H.Hara	Bark	China	SE, PP, CC, rp-CC, NMR	[144]
	*Olea europaea* L.	Wood	Spain	SER, CC, rp-HPLC-DAD, NMR	[135]
			Spain (different populations)	SE, HPLC-DAD, HPLC-DAD-MS	[136]
	*Syringa oblata* subsp. *dilatata* (Nakai) P.S.Green & M.C.Chang	Twigs	South Korea	SE, PP, CC, rp-CC, rp-HPLC-UV, NMR	[142]
Jasuroside A (Figure 20)	*Jasminum urophyllum* Hemsl.	Whole plant	Taiwan	SE, PP, CC, CPC, p-TLC, α_[D]_, IR, UV, NMR, MS	[145]
	*Jasminum nudiflorum* Lindl.	Leaves and stems	Japan (obtained from a botanical garden)	HSE, PP, CC, p-TLC, α_[D]_, IR, UV, NMR, HR-MS	[146]
Jasuroside C (Figure 20)	*Jasminum urophyllum* Hemsl.	Whole plant	Taiwan	SE, PP, CC, CPC, p-TLC, α_[D]_, IR, UV, NMR, MS	[145]
	*Jasminum nudiflorum* Lindl.	Leaves and stems	Japan (obtained from a botanical garden)	HSE, PP, CC, p-TLC, α_[D]_, IR, UV, NMR, HR-MS	[146]
Jasuroside G (Figure 20)	*Jasminum urophyllum* Hemsl.	Leaves and stems	Taiwan	SE, PP, CC, rp-CC, α_[D]_, IR, UV, NMR, MS	[147]
Kickxin (Figure 1)	*Kickxia commutata* (Bernh. ex Rchb.) Fritsch	Flowering aerial parts	Bulgaria	SE, ACT, CC, α_[D]_, NMR	[148]
	*Kickxia elatine* (L.) Dumort.	Flowering aerial parts	Bulgaria	SE, ACT, CC, α_[D]_, NMR	[148]
	*Kickxia spuria* (L.) Dumort.	Flowering aerial parts	Bulgaria	SE, ACT, CC, α_[D]_, NMR	[148]
Korolkoside (Figure 17)	*Lonicera korolkowii* Stapf	Aerial parts	Japan (purchased from a company)	SE, PP, CC, rp-HPLC-UV, α_[D]_, NMR, HR-MS	[149]
	*Lonicera japonica* Thunb.	n.a.	n.a.	n.a.	[150]
Kurdnestorianoside (Figure 11)	*Pterocephalus nestorianus* Nábelek	Flowers	Iraq	DP, SE, MPLC, α_[D]_, IR, UV, NMR, HR-MS	[67]
Laciniatoside I (Figure 31)	*Dipsacus laciniatus* L.	Aerial parts	Hungary	SE, PP, CCD, CC, α_[D]_, IR, UV, NMR	[45]
	*Cephalaria scoparia* Contandr. & Quézel	Whole plant	Turkey	SE, PP, rp-MPLC, MPLC, NMR	[151]
	*Cephalaria gazipashensis* Sümbül	Aerial parts	Turkey	SE, PP, DF, rp-VLC, CC, MPLC, NMR	[152]
	*Pterocephalus hookeri* (C.B.Clarke) E.Pritz.	Underground parts	Tibet	SER, PP, CC rp-CC, NMR	[58]
		Underground parts	Tibet	SER, PP, TLC, sp-HPLC-MS, NMR	[59]
		n.a.	n.a.	n.a.	[60]
		Whole plant	China	USE, UPLC-MS^n^	[64]
Laciniatoside II (Figure 30)	*Dipsacus laciniatus* L.	Aerial parts	Hungary	SE, PP, CCD, CC, α_[D]_, IR, UV, NMR	[45]
	*Linnaea chinensis* A.Braun & Vatke	Aerial parts	Italy	SE, PP, CC, NMR	[11]
	*Dipsacus ferox* Loisel.	Leaves and branches	Italy	SE, CC, NMR	[153]
	*Pterocephalus hookeri* (C.B.Clarke) E.Pritz.	Underground parts	Tibet	SER, PP, CC rp-CC, NMR	[58]
		Underground parts	Tibet	SER, PP, TLC, sp-HPLC-MS, NMR	[59]
		n.a.	n.a.	n.a.	[60]
		Whole plant	China	SE, PP, HPLC-UV	[62]
				USE, UPLC-MS^n^	[64]
			Tibet	SE, PP, CC, sp-HPLC-UV, NMR	[24]
	*Handroanthus impetiginosus* (Mart. ex DC.) Mattos	Leaves	Egypt (obtained from a botanical garden)	PE, PP, HPLC-MS^n^	[154]
Laciniatoside III (Figure 29)	*Dipsacus laciniatus* L.	Aerial parts	Hungary	SE, PP, CCD, CC, α_[D]_, IR, UV, NMR	[45]
Laciniatoside IV (Figure 30)	*Dipsacus laciniatus* L.	Aerial parts	Hungary	SE, PP, CCD, CC, α_[D]_, IR, UV, NMR	[45]
Laciniatoside V (Figure 30)	*Dipsacus laciniatus* L.	Flowering aerial parts	Hungary	SE, CC, CCC, α_[D]_, IR, UV, NMR	[155]
		Aerial parts		SE, PP, CCD, CC, α_[D]_, IR, UV, NMR	[45]
	*Cephalaria balansae* Raus	Whole plant	Turkey	USE, PP, HPLC-MS^n^	[156]
	*Cephalaria elmaliensis* Hub.-Mor. & V.A.Matthews	Whole plant	Turkey	USE, PP, HPLC-MS^n^	[156]
	*Cephalaria isaurica* V.A.Matthews	Whole plant	Turkey	USE, PP, HPLC-MS^n^	[156]
	*Cephalaria scoparia* Contandr. & Quézel	Whole plant	Turkey	USE, PP, HPLC-MS^n^	[156]
	*Cephalaria speciosa* Boiss. & Kotschy	Whole plant	Turkey	USE, PP, HPLC-MS^n^	[156]
	*Cephalaria stellipilis* Boiss.	Whole plant	Turkey	USE, PP, HPLC-MS^n^	[156]
	*Cephalaria sumbuliana* Göktürk	Whole plant	Turkey	USE, PP, HPLC-MS^n^	[156]
	*Scabiosa atropurpurea* L.	Whole plant	Turkey	SE, CC, sp-HPLC-UV, HPLC-MS^n^, NMR	[34]
Lasianoside G (Figure 4)	*Lasianthus verticillatus* (Lour.) Merr.	Levaes	Japan	SE, PP, rp-CC, HPLC-UV, α_[D]_, IR, UV, NMR, MS	[157]
Lasianoside H (Figure 5)	*Lasianthus verticillatus* (Lour.) Merr.	Levaes	Japan	SE, PP, rp-CC, HPLC-UV, α_[D]_, IR, UV, NMR, MS	[157]
Lasianoside I (Figure 5)	*Lasianthus verticillatus* (Lour.) Merr.	Levaes	Japan	SE, PP, rp-CC, HPLC-UV, α_[D]_, IR, UV, NMR, MS	[157]
Liguside A (Figure 20)	*Ligustrum lucidum* W.T.Aiton	Fruits	China	SER, PP, CC, p-HPLC-UV, MP, α_[D]_, IR, UV, NMR, HR-MS	[119]
Liguside B (Figure 20)	*Ligustrum lucidum* W.T.Aiton	Fruits	China	SER, PP, CC, p-HPLC-UV, MP, α_[D]_, IR, UV, NMR, HR-MS	[119]
	*Ilex pubescens* Hook. & Arn.	Roots	China (purchased from a company)	SE, PP, CC, rp-HPLC-UV, NMR, HR-MS	[158]
Ligustrinoside (Figure 1)	*Strychnos lucida* R.Br.	Wood	Indonesia	SE, PP, CC, MPLC, α_[D]_, IR, UV, NMR, MS	[159]
Lisianthoside (Figure 23)	*Lisianthius jefensis* A.Robyns & T.S.Elias	n.r.	n.r.	SE, CC, sp-HPLC-UV, NMR	[160]
	*Dipsacus inermis* Wall.	Roots	China	HSE, PP, CC, rp-CC, p-TLC, rp-HPLC-UV, NMR	[48]
Loasafolioside (Figure 30)	*Loasa acerifolia* Dombey ex A.Juss.	Leaves	Germany (obtained from a botanical garden)	SXE, PP, CC, sp-HPLC-UV, α_[D]_, IR, UV, NMR, MS	[161]
Longifloroside (Figure 3)	*Pedicularis longiflora* Rudolph	Whole plant	China	SE, SER, DP, PP, CC, NMR, MS	[162]
Minutifloroside (Figure 6)	*Palicourea minutiflora* (Müll.Arg.) C.M.Taylor	Leaves and branches	Brazil	SE, PP, CC, α_[D]_, NMR, HR-MS	[163]
Molihuaside A (Figure 16)	*Jasminum sambac* (L.) Aiton	Flowers	China	SER, PP, CC, rp-CC, MP, α_[D]_, IR, UV, NMR, MS	[164]
	*Jasminum flexile* Vahl	Aerial parts	India	SE, PP, CC, p-TLC, NMR, MS	[165]
Molihuaside C (Figure 16)	*Jasminum sambac* (L.) Aiton	Flowers	China	SER, PP, CC, rp-CC, MP, α_[D]_, IR, UV, NMR, MS	[164]
Molihuaside D (Figure 16)	*Jasminum sambac* (L.) Aiton	Flowers	China	SER, PP, CC, rp-CC, MP, α_[D]_, IR, UV, NMR, MS	[164]
		Leaves and stems	Taiwan	SE, PP, CC, p-TLC, α_[D]_, NMR	[166]
Molihuaside E (Figure 16)	*Jasminum sambac* (L.) Aiton	Flowers	China	SER, PP, CC, rp-CC, MP, α_[D]_, IR, UV, NMR, MS	[164]
Neo-cornuside C (Figure 12)	*Cornus officinalis* Siebold & Zucc.	Fruits	China	SER, PP, CC, sp-HPLC-UV, α_[D]_, IR, UV, NMR, HR-MS	[167]
Neo-cornuside D (Figure 23)	*Cornus officinalis* Siebold & Zucc.	Fruits	China	SER, PP, CC, sp-HPLC-UV, α_[D]_, IR, UV, NMR, HR-MS	[167]
Neo-cornuside F (Figure 23)	*Cornus officinalis* Siebold & Zucc.	Fruits	China (purchased from a local market)	SER, PP, CC, sp-HPLC-UV, α_[D]_, IR, UV, NMR, HR-MS	[100]
Neo-polyanoside (Figure 15)	*Jasminum polyanthum* Franch.	Flowers	China (purcahsed from a company)	HSE, PP, CC, p-TLC, p-HPLC-UV, α_[D]_, IR, UV, NMR, HR-MS	[168]
Nudifloside A (Figure 14)	*Jasminum nudiflorum* Lindl.	Stems	Japan (obtained from a botanical garden)	HSE, PP, CC, p-HPLC-UV, α_[D]_, IR, UV, NMR, HR-MS	[140]
Nudifloside B (Figure 14)	*Jasminum nudiflorum* Lindl.	Stems	Japan (obtained from a botanical garden)	HSE, PP, CC, p-HPLC-UV, α_[D]_, IR, UV, NMR, HR-MS	[140]
Officinaloside A (Figure 21)	*Cornus officinalis* Siebold & Zucc.	Twigs	China	SE, PP, CC, rp-CC, HPLC-UV, α_[D]_, IR, UV, NMR, HR-MS	[169]
Oleoneonuezhenide	*Syringa vulgaris* L.	Bark	Poland	HSE, HPLC-DAD-MS^n^	[170]
Oleonuezhenide (Figure 15)	*Ligustrum japonicum* Thunb.	Fruits	Japan (purchased from a company)	SE, PP, CC, rp-CC, α_[D]_, IR, UV, NMR, MS	[171]
		Leaves	South Korea	USE, PP, CC, rp-CC, sp-HPLC-UV, NMR	[172]
	*Ligustrum obtusifolium* Siebold & Zucc.	Leaves	n.a.	n.a.	[173]
	*Ligustrum lucidum* W.T.Aiton	Fruits	China	SER, PP, CC, p-HPLC-UV, MP, α_[D]_, IR, UV, NMR, HR-MS	[119]
		Dried fruits		SE, PP, CC, NMR	[116]
			n.a.	n.a.	[174]
			China	USE, UHPLC-MS^n^	[113]
			China (purchased from a company)	SE, CC, HPLC-DAD, HPLC-MS	[175]
	*Ilex pubescens* Hook. & Arn.	Roots	China (purchased from a company)	SE, PP, CC, rp-HPLC-UV, NMR, HR-MS	[158]
	*Syringa oblata* subsp. *dilatata* (Nakai) P.S.Green & M.C.Chang	Twigs	South Korea	SE, PP, CC, rp-CC, rp-HPLC-UV, NMR	[142]
	*Ligustrum japonicum* Thunb.	Dried fruits	South Korea	SE, PP, CC, rp-HPLC-UV, NMR, MS	[122]
	*Syringa vulgaris* L.	Flowers	Poland	HSE, CC, p-HPLC-UV, α_[D]_, UV, NMR, HR-MS	[103]
		Whole plant		HSE, HPLC-DAD-MS^n^	[176]
		Bark		HSE, HPLC-DAD-MS^n^	[170]
Paederoscandoside (Figure 3)	*Paederia foetida* L.	n.a.	n.a.	n.a.	[177]
		Stems	China (purchased from a company)	SE, PP, CC, p-HPLC-UV, NMR	[178]
		Aerial parts	China	SER, CC, sp-HPLC-UV, NMR	[33]
Paederoside B (Figure 7)	*Paederia foetida* L.	Stems	China	SE, PP, CC, rp-CC, HPLC-UV, α_[D]_, IR, UV, NMR, HR-MS	[179]
		Whole plant		SER, PP, HPLC-MS^n^, HR-MS^n^	[180]
		Stems	China (purchased from a company)	SE, PP, CC, HPLC-MS	[178]
Patriscabiobisin A (Figure 34)	*Patrinia scabiosifolia* Link	Whole plant	China	SE, PP, CC, sp-HPLC-UV, α_[D]_, IR, UV, NMR, HR-MS	[181]
Patriscabiobisin B (Figure 34)	*Patrinia scabiosifolia* Link	Whole plant	China	SE, PP, CC, sp-HPLC-UV, α_[D]_, IR, UV, NMR, HR-MS	[181]
Patriscabiobisin C (Figure 27)	*Patrinia scabiosifolia* Link	Whole plant	China	SE, PP, CC, sp-HPLC-UV, α_[D]_, IR, UV, NMR, HR-MS	[181]
				SE, PP, CC, sp-HPLC-UV, α_[D]_, IR, UV, NMR, HR-MS	[182]
Phukettoside A (Figure 33)	*Gynochthodes umbellata* (L.) Razafim. & B.Bremer	Leaves	Thailand	SE, CC, p-HPLC-UV, α_[D]_, IR, UV, NMR, HR-MS	[183]
Phukettoside B (Figure 33)	*Gynochthodes umbellata* (L.) Razafim. & B.Bremer	Leaves	Thailand	SE, CC, p-HPLC-UV, α_[D]_, IR, UV, NMR, HR-MS	[183]
Phukettoside C (Figure 33)	*Gynochthodes umbellata* (L.) Razafim. & B.Bremer	Leaves	Thailand	SE, CC, p-HPLC-UV, α_[D]_, IR, UV, NMR, HR-MS	[183]
Phukettoside D (Figure 2)	*Gynochthodes umbellata* (L.) Razafim. & B.Bremer	Leaves	Thailand	SE, CC, p-HPLC-UV, α_[D]_, IR, UV, NMR, HR-MS	[183]
Picconioside I (Figure 7)	*Picconia excelsa* (Aiton) DC.	Foliage	Spain	SE, PP, CC, α_[D]_, NMR	[184]
	*Strychnos lucida* R.Br.	Bark and wood	Thailand	HSE, PP, MPLC, rp-MPLC, p-HPLC-UV, NMR	[54]
	*Leonotis nepetifolia* (L.) R.Br.	Aerial parts	Vietnam	SE, PP, CC, rp-CC, NMR, MS	[185]
Picconioside II (Figure 34)	*Galium maximowiczii* (Kom.) Pobed.	Whole plant	South Korea	SE, PP, CC, p-HPLC-UV, NMR	[32]
Picrorhizaoside E (Figure 32)	*Picrorhiza kurroa* Royle ex Benth.	Rhizomes	China (cultivated)	SER, PP, CC, rp-CC, HPLC-UV, α_[D]_, IR, UV, NMR, HR-MS	[186]
Picrorhizaoside F (Figure 32)	*Picrorhiza kurroa* Royle ex Benth.	Rhizomes	China (cultivated)	SER, PP, CC, rp-CC, HPLC-UV, α_[D]_, IR, UV, NMR, HR-MS	[186]
Picrorhizaoside G (Figure 32)	*Picrorhiza kurroa* Royle ex Benth.	Rhizomes	China (cultivated)	SER, PP, CC, rp-CC, HPLC-UV, α_[D]_, IR, UV, NMR, HR-MS	[186]
Polyanoside (Figure 15)	*Jasminum polyanthum* Franch.	Flowers	China (purcahsed from a company)	HSE, PP, CC, p-TLC, p-HPLC-UV, α_[D]_, IR, UV, NMR, HR-MS	[134]
	*Jasminum sambac* (L.) Ait	Leaves	Egypt (different populations)	PE, HPLC-PDA-MS^n^	[187]
	*Jasminum multiflorum* (Burm.f.) Andrews	Leaves	Egypt	PE, PP, VLC, HPLC-PDA-MS^n^	[188]
Premnaodoroside D (Figure 4)	*Premna odorata* Blanco	Leaves	Japan	SE, PP, CC, rp-CC, DCCC, HPLC-UV, α_[D]_, IR, UV, NMR, HR-MS	[189]
		Leaves	Egypt	SE, PP, HPLC-MS	[190]
Premnaodoroside E (Figure 4)	*Premna odorata* Blanco	Leaves	Japan	SE, PP, CC, rp-CC, DCCC, HPLC-UV, α_[D]_, IR, UV, NMR, HR-MS	[189]
Premnaodoroside F	*Premna odorata* Blanco	Leaves	Japan	SE, PP, CC, rp-CC, DCCC, HPLC-UV, α_[D]_, IR, UV, NMR, HR-MS	[189]
Premnaodoroside G	*Premna odorata* Blanco	Leaves	Japan	SE, PP, CC, rp-CC, DCCC, HPLC-UV, α_[D]_, IR, UV, NMR, HR-MS	[189]
Ptehoside C (Figure 31)	*Pterocephalus hookeri* (C.B.Clarke) E.Pritz.	Whole plant	Tibet	SE, PP, CC, sp-HPLC-UV, α_[D]_, IR, UV, NMR, HR-MS	[24]
Ptehoside D (Figure 31)	*Pterocephalus hookeri* (C.B.Clarke) E.Pritz.	Whole plant	Tibet	SE, PP, CC, sp-HPLC-UV, α_[D]_, IR, UV, NMR, HR-MS	[24]
Ptehoside E (Figure 31)	*Pterocephalus hookeri* (C.B.Clarke) E.Pritz.	Whole plant	Tibet	SE, PP, CC, sp-HPLC-UV, α_[D]_, IR, UV, NMR, HR-MS	[24]
Ptehoside F (Figure 31)	*Pterocephalus hookeri* (C.B.Clarke) E.Pritz.	Whole plant	Tibet	SE, PP, CC, sp-HPLC-UV, α_[D]_, IR, UV, NMR, HR-MS	[24]
Ptehoside G (Figure 31)	*Pterocephalus hookeri* (C.B.Clarke) E.Pritz.	Whole plant	Tibet	SE, PP, CC, sp-HPLC-UV, α_[D]_, IR, UV, NMR, HR-MS	[24]
Ptehoside H (Figure 31)	*Pterocephalus hookeri* (C.B.Clarke) E.Pritz.	Whole plant	Tibet	SE, PP, CC, sp-HPLC-UV, α_[D]_, IR, UV, NMR, HR-MS	[24]
Ptehoside I (Figure 31)	*Pterocephalus hookeri* (C.B.Clarke) E.Pritz.	Whole plant	Tibet	SE, PP, CC, sp-HPLC-UV, α_[D]_, IR, UV, NMR, HR-MS	[24]
Pterhookeroside (Figure 28)	*Pterocephalus hookeri* (C.B.Clarke) E.Pritz.	Underground parts	Tibet	SER, PP, CC, sp-HPLC-UV, α_[D]_, IR, UV, NMR, HR-MS	[191]
Pterocenoid B (Figure 28)	*Pterocephalus hookeri* (C.B.Clarke) E.Pritz.	Underground parts	Tibet	SER, PP, CC, sp-HPLC-UV, α_[D]_, IR, UV, NMR, HR-MS	[192]
		Whole plant	China	SE, PP, CC, rp-CC, HPLC-UV, NMR	[193]
Pterocenoid C (Figure 28)	*Pterocephalus hookeri* (C.B.Clarke) E.Pritz.	Underground parts	Tibet	SER, PP, CC, sp-HPLC-UV, α_[D]_, IR, UV, NMR, HR-MS	[192]
Pterocenoid D (Figure 28)	*Pterocephalus hookeri* (C.B.Clarke) E.Pritz.	Underground parts	Tibet	SER, PP, CC, sp-HPLC-UV, α_[D]_, IR, UV, NMR, HR-MS	[192]
Pterocenoid E (Figure 28)	*Pterocephalus hookeri* (C.B.Clarke) E.Pritz.	Whole plant	China	SE, PP, CC, rp-CC, HPLC-UV, α_[D]_, UV, NMR, HR-MS	[193]
Pterocenoid F (Figure 28)	*Pterocephalus hookeri* (C.B.Clarke) E.Pritz.	Whole plant	China	SE, PP, CC, rp-CC, HPLC-UV, α_[D]_, UV, NMR, HR-MS	[193]
Pterocenoid G (Figure 33)	*Pterocephalus hookeri* (C.B.Clarke) E.Pritz.	Whole plant	China	SE, PP, CC, rp-CC, HPLC-UV, α_[D]_, UV, NMR, HR-MS	[193]
Pterocenoid H (Figure 28)	*Pterocephalus hookeri* (C.B.Clarke) E.Pritz.	Whole plant	China	SE, PP, CC, rp-CC, HPLC-UV, α_[D]_, UV, NMR, HR-MS	[193]
Pterocephaline (Figure 11)	*Pterocephalus pinardi* Boiss.	Aerial parts	Turkey	SE, PP, rp-VLC, CC, α_[D]_, IR, NMR, HR-MS	[55]
	*Pterocephalus hookeri* (C.B.Clarke) E.Pritz.	Whole plant	China	USE, UPLC-MS^n^	[64]
	*Dipsacus inermis* Wall.	Roots	China	HSE, PP, CC, p-TLC, p-rp-HPLC-UV, NMR	[106]
			China (purchased from a local market)	SER, PP, MPLC, p-HPLC-UV, α_[D]_, IR, UV, NMR, HR-MS	[108]
Pteroceside A (Figure 9)	*Pterocephalus hookeri* (C.B.Clarke) E.Pritz.	Underground parts	Tibet	SER, PP, CC, rp-CC, sp-HPLC-UV, α_[D]_, IR, UV, NMR, HR-MS	[58]
	*Scabiosa atropurpurea* L.	Whole plant	Turkey	SE, CC, sp-HPLC-UV, HPLC-MS^n^, NMR	[34]
Pteroceside B (Figure 9)	*Pterocephalus hookeri* (C.B.Clarke) E.Pritz.	Underground parts	Tibet	SER, PP, CC, rp-CC, sp-HPLC-UV, α_[D]_, IR, UV, NMR, HR-MS	[58]
Pteroceside C (Figure 9)	*Pterocephalus hookeri* (C.B.Clarke) E.Pritz.	Underground parts	Tibet	SER, PP, CC, rp-CC, sp-HPLC-UV, α_[D]_, IR, UV, NMR, HR-MS	[58]
	*Scabiosa atropurpurea* L.	Whole plant	Turkey	SE, CC, sp-HPLC-UV, HPLC-MS^n^, NMR	[34]
Pubescensoside (Figure 6)	*Anarrhinum forskaohlii* subsp. *pubescens* D.A.Sutton	Aerial parts	Egypt	SE, DP, PP, CC, NMR, HR-MS	[194]
Pubzenoside (Figure 23)	*Ilex pubescens* Hook. & Arn.	Roots	China (purchased from a company)	SER, PP, CC, rp-HPLC-UV, α_[D]_, IR, UV, NMR, HR-MS	[195]
Radiatoside (Figure 1)	*Argylia radiata* (L.) D.Don	Whole plant	Chile	SE, ACT, PC, TLC, CC, α_[D]_, IR, UV, NMR	[196]
Radiatoside B (Figure 1)	*Argylia radiata* (L.) D.Don	Whole plant	Chile	SE, ACT, PC, TLC, CC, α_[D]_, IR, UV, NMR	[197]
Radiatoside C (Figure 1)	*Argylia radiata* (L.) D.Don	Whole plant	Chile	SE, ACT, PC, TLC, CC, α_[D]_, IR, UV, NMR	[197]
Radiatoside D (Figure 1)	*Argylia radiata* (L.) D.Don	Whole plant	Chile	SE, ACT, PC, TLC, α_[D]_, IR, UV, NMR	[198]
Radiatoside E (Figure 1)	*Argylia radiata* (L.) D.Don	Whole plant	Chile	SE, CC, α_[D]_, IR, UV, NMR, MS	[30]
Radiatoside F (Figure 1)	*Argylia radiata* (L.) D.Don	Whole plant	Chile	SE, CC, α_[D]_, IR, UV, NMR, MS	[30]
Randinoside (Figure 1)	*Catunaregam spinosa* (Thunb.) Tirveng.	Stems	Brazil	SE, PP, CC, p-HPLC-UV, α_[D]_, IR, UV, NMR, HR-MS	[199]
Rapulaside A (Figure 34)	*Heracleum rapula* Franch.	Roots	China	SE, PP, CC, p-HPLC-UV, α_[D]_, NMR, MS	[200]
Rapulaside B (Figure 34)	*Heracleum rapula* Franch.	Roots	China	SE, PP, CC, p-HPLC-UV, α_[D]_, NMR, MS	[200]
Reticunin A (Figure 27)	*Neonauclea reticulata* (Havil.) Merr.	Stems	Taiwan	SE, PP, CC, HPLC-UV, α_[D]_, IR, UV, NMR, HR-MS	[201]
Reticunin B (Figure 27)	*Neonauclea reticulata* (Havil.) Merr.	Stems	Taiwan	SE, PP, CC, HPLC-UV, α_[D]_, IR, UV, NMR, HR-MS	[201]
Rotunduside (Figure 1)	*Cyperus rotundus* L.	Rhizomes	China	SER, PP, CC, α_[D]_, IR, NMR, HR-MS	[202]
Rotunduside A (Figure 2)	*Cyperus rotundus* L.	Rhizomes	China	SER, PP, CC, α_[D]_, IR, NMR, HR-MS	[203]
Safghanoside G (Figure 19)	*Syringa persica* L.	Leaves	Japan (obtained from a botanical garden)	HSE, PP, CC, p-TLC, p-HPLC-UV, α_[D]_, IR, UV, NMR, HR-MS	[204]
	*Fraxinus mandshurica* Rupr.	Seeds	China (purchased from a company)	SE, PP, CC, HPLC-DAD, NMR	[123]
Safghanoside H (Figure 19)	*Syringa persica* L.	Leaves	Japan (obtained from a botanical garden)	HSE, PP, CC, p-TLC, p-HPLC-UV, α_[D]_, IR, UV, NMR, HR-MS	[204]
Salvialoside E (Figure 28)	*Salvia digitaloides* Diels	Roots	China	SER, PP, CC, α_[D]_, IR, UV, NMR, HR-MS	[205]
Saprosmoside A (Figure 6)	*Saprosma scortechinii* King & Gamble	Leaves and stems	Malaysia	SE, PP, CC, α_[D]_, IR, UV, NMR, HR-MS	[206]
Saprosmoside B (Figure 5)	*Saprosma scortechinii* King & Gamble	Leaves and stems	Malaysia	SE, PP, CC, α_[D]_, IR, UV, NMR, HR-MS	[206]
Saprosmoside C (Figure 3)	*Saprosma scortechinii* King & Gamble	Leaves and stems	Malaysia	SE, PP, CC, α_[D]_, IR, UV, NMR, HR-MS	[206]
Saprosmoside D (Figure 3)	*Saprosma scortechinii* King & Gamble	Leaves and stems	Malaysia	SE, PP, CC, α_[D]_, IR, UV, NMR, HR-MS	[206]
	*Paederia foetida* L.	Stems	China (purchased from a company)	SE, PP, CC, p-HPLC-UV, NMR	[178]
Saprosmoside E (Figure 4)	*Saprosma scortechinii* King & Gamble	Leaves and stems	Malaysia	SE, PP, CC, α_[D]_, IR, UV, NMR, HR-MS	[206]
	*Paederia foetida* L.	Stems	China	SE, PP, CC, rp-CC, NMR	[179]
		Whole plant		SER, PP, HPLC-MS^n^, HR-MS^n^	[180]
		Stems	China (purchased from a company)	SE, PP, CC, p-HPLC-UV, NMR	[178]
		Aerial parts	China	SER, CC, sp-HPLC-UV, NMR	[33]
Saprosmoside F (Figure 3)	*Saprosma scortechinii* King & Gamble	Leaves and stems	Malaysia	SE, PP, CC, α_[D]_, IR, UV, NMR, HR-MS	[206]
	*Paederia foetida* L.	Stems	China (purchased from a company)	SE, PP, CC, HPLC-MS	[178]
		Aerial parts	China	SER, CC, sp-HPLC-UV, NMR	[33]
Saprosmoside G (Figure 7)	*Saprosma scortechinii* King & Gamble	Leaves and stems	Malaysia	SE, PP, CC, α_[D]_, IR, UV, NMR, HR-MS	[207]
Saprosmoside H (Figure 2)	*Saprosma scortechinii* King & Gamble	Leaves and stems	Malaysia	SE, PP, CC, α_[D]_, IR, UV, NMR, HR-MS	[207]
Saungmaygaoside A (Figure 10)	*Picrorhiza kurroa* Royle ex Benth.	Stems	Myanmar	USE, PP, CC, p-TLC, α_[D]_, IR, UV, NMR, HR-MS	[23]
Saungmaygaoside B (Figure 10)	*Picrorhiza kurroa* Royle ex Benth.	Stems	Myanmar	USE, PP, CC, sp-HPLC-UV, α_[D]_, IR, UV, NMR, HR-MS	[23]
Saungmaygaoside C (Figure 10)	*Picrorhiza kurroa* Royle ex Benth.	Stems	Myanmar	USE, PP, CC, sp-HPLC-UV, α_[D]_, IR, UV, NMR, HR-MS	[23]
Saungmaygaoside D (Figure 10)	*Picrorhiza kurroa* Royle ex Benth.	Stems	Myanmar	USE, PP, CC, p-TLC, α_[D]_, IR, UV, NMR, HR-MS	[23]
Scaevoloside (Figure 31)	*Scaevola racemigera* Däniker	Aerial parts	New Caledonia	SE, CC, α_[D]_, IR, UV, NMR	[44]
Sclerochitonoside C (Figure 12)	*Sclerochiton harveyanus* Nees	Leaves	England (obtained from a botanical garden)	SE, PP, CC, HPLC-UV, NMR, MS	[208]
Seemannoside A (Figure 18)	*Lisianthius seemanii* Perkins	Aerial parts	Panama	SE, CC, rp-MPLC, sp-HPLC-UV-NMR, MP, α_[D]_, IR, MS	[209]
Seemannoside B (Figure 18)	*Lisianthius seemanii* Perkins	Aerial parts	Panama	SE, CC, rp-MPLC, sp-HPLC-UV-NMR, MP, α_[D]_, IR, MS	[209]
Semipapposiridoid A (Figure 9)	*Scabiosa semipapposa* Salzm. ex DC.	Aerial parts	Algeria	SE, rp-VLC, FC, rp-MPLC, α_[D]_, IR, UV, NMR, HR-MS	[210]
Semipapposiridoid B (Figure 9)	*Scabiosa semipapposa* Salzm. ex DC.	Aerial parts	Algeria	SE, rp-VLC, FC, rp-MPLC, α_[D]_, IR, UV, NMR, HR-MS	[210]
Semipapposiridoid C (Figure 9)	*Scabiosa semipapposa* Salzm. ex DC.	Aerial parts	Algeria	SE, rp-VLC, FC, rp-MPLC, α_[D]_, IR, UV, NMR, HR-MS	[210]
Semipapposiridoid D (Figure 9)	*Scabiosa semipapposa* Salzm. ex DC.	Aerial parts	Algeria	SE, rp-VLC, FC, rp-MPLC, α_[D]_, IR, UV, NMR, HR-MS	[210]
Semipapposiridoid E (Figure 31)	*Scabiosa semipapposa* Salzm. ex DC.	Aerial parts	Algeria	SE, rp-VLC, FC, rp-MPLC, α_[D]_, IR, UV, NMR, HR-MS	[210]
Semipapposiridoid F (Figure 31)	*Scabiosa semipapposa* Salzm. ex DC.	Aerial parts	Algeria	SE, rp-VLC, FC, rp-MPLC, α_[D]_, IR, UV, NMR, HR-MS	[210]
Septemfidoside (Figure 10)	*Gentiana septemfida* Pall.	Aerial parts	Turkey	SE, PP, CC, MPLC, α_[D]_, IR, UV, NMR, HR-MS	[211]
		Whole plant	Azerbaijan	SE, HPLC-DAD, HPLC-DAD-MS^n^	[212]
	*Gentiana olivieri* Griseb.	Whole plant	Uzbekistan	SE, SER, PP, CC, p-HPLC-UV, NMR	[213]
	*Gentiana lutea* L.	Leaves	Montenegro (different populations)	USE, HPLC-DAD, HPLC-MS^n^	[214]
	*Lomelosia stellata* (L.) Raf.	Whole plant	Algeria	SE, CC, CPC, FC, HPLC-UV, NMR	[12]
Strychoside A (Figure 17)	*Strychnos spinosa* Lam.	Branches	Japan (cultivated)	HSE, PP, rp-MPLC, p-HPLC-UV, p-TLC, α_[D]_, IR, UV, NMR, HR-MS	[53]
Swerilactone A (Figure 33)	*Swertia mileensis* T.N.Ho & W.L.Shih	Whole plant	China	SER, PP, CC, rp-CC, MP, α_[D]_, IR, UV, NMR, HR-MS	[215]
Swerilactone B (Figure 33)	*Swertia mileensis* T.N.Ho & W.L.Shih	Whole plant	China	SER, PP, CC, rp-CC, MP, α_[D]_, IR, UV, NMR, HR-MS	[215]
Swerilactoside A (Figure 21)	*Swertia mileensis* T.N.Ho & W.L.Shih	Whole plant	China	SER, PP, CC, sp-HPLC-UV, α_[D]_, IR, UV, NMR, HR-MS	[216]
Swerilactoside B (Figure 21)	*Swertia mileensis* T.N.Ho & W.L.Shih	Whole plant	China	SER, PP, CC, sp-HPLC-UV, α_[D]_, IR, UV, NMR, HR-MS	[216]
Swerilactoside C (Figure 21)	*Swertia mileensis* T.N.Ho & W.L.Shih	Whole plant	China	SER, PP, CC, α_[D]_, IR, UV, NMR, HR-MS	[216]
Swertianoside A (Figure 22)	*Swertia angustifolia Buch.-Ham. ex D.Don*	Whole plant	China	SER, PP, CC, α_[D]_, IR, UV, NMR, HR-MS	[217]
Sylvestroside I (Figure 9)	*Dipsacus fullonum* L.	Seeds	Denmark	SE, p-TLC, α_[D]_, UV, NMR	[41]
	*Acicarpha tribuloides* Juss.	Aerial parts	Peru	SE, PP, CC, HPLC-UV, α_[D]_, NMR, MS	[218]
	*Linnaea chinensis* A.Braun & Vatke	Aerial parts	Italy	SE, PP, CC, NMR	[11]
	*Strychnos lucida* R.Br.	Bark and wood	Thailand	HSE, PP, MPLC, rp-MPLC, p-HPLC-UV, NMR	[54]
	*Pterocephalus hookeri* (C.B.Clarke) E.Pritz.	Underground parts	Tibet	SER, PP, CC rp-CC, NMR	[58]
		Aerial parts	n.a.	n.a.	[17]
		Whole plant	China	SE, PP, HPLC-UV	[62]
				SER, CC, UPLC-PDA	[63]
		Underground parts	Tibet	SER, PP, TLC, sp-HPLC-MS, NMR	[59]
		n.a.	n.a.	n.a.	[60]
		Whole plant	China	USE, UPLC-MS^n^	[64]
		Whole plant	Tibet	SE, PP, CC, p-HPLC-UV, p-TLC, NMR	[65]
			China (different populations)	USE, UPLC-MS^n^	[66]
	*Lomelosia stellata* (L.) Raf.	Whole plant	Algeria	SE, CC, CPC, FC, HPLC-UV, NMR	[12]
	*Scabiosa atropurpurea* L.	Whole plant	Turkey	SE, CC, sp-HPLC-UV, HPLC-MS^n^	[34]
	*Dipsacus inermis* Wall.	Roots	China (purchased from a company)	SER, PP, MPLC, p-TLC, NMR	[50]
		Dried Roots	China (different populations)	SE, CC, UHPLC-PDA, UHPLC-MS^n^	[52]
		n.a.	n.a.	n.a.	[219]
	*Scabiosa semipapposa Salzm. ex DC.*	Aerial parts	Algeria	SE, rp-VLC, FC, rp-MPLC, NMR	[210]
Sylvestroside II (Figure 9)	*Dipsacus fullonum* L.	Seeds	Denmark	SE, p-TLC, α_[D]_, UV, NMR	[41]
	*Abelia grandiflora* (Rovelli ex André) Rehder	Leaves	Japan	HSE, PP, ACT, CC, p-TLC, PLC, NMR	[22]
	*Linnaea chinensis* A.Braun & Vatke	Aerial parts	Italy	SE, PP, CC, NMR	[11]
	*Scabiosa semipapposa Salzm. ex DC.*	Aerial parts	Algeria	SE, rp-VLC, FC, rp-MPLC, NMR	[210]
Sylvestroside III (Figure 30)	*Dipsacus fullonum* L.	Seeds	Denmark	SE, p-TLC, α_[D]_, UV, NMR	[41]
		Leaves	Poland	USE, UHPLC-PDA-MS^n^	[42]
		Roots	Poland	USE, UHPLC-PDA-MS^n^	[42]
		Leaves	Estonia	DESE, HPLC-DAD-MS	[220]
		Leaves	Estonia	SE, CC, rp-FC, HPLC-DAD-MS, NMR	[221]
	*Scaevola montana Labill.*	Aerial parts	New Caledonia	SE, CC, NMR	[43]
	*Scaevola racemigera* Däniker	Aerial parts	New Caledonia	SE, CC, NMR	[44]
	*Dipsacus laciniatus* L.	Aerial parts	Hungary	SE, PP, CCD, CC, α_[D]_, IR, UV, NMR	[45]
	*Acicarpha tribuloides* Juss.	Aerial parts	Peru	SE, PP, CC, HPLC-UV, α_[D]_, NMR, MS	[218]
	*Linnaea chinensis* A.Braun & Vatke	Aerial parts	Italy	SE, PP, CC, NMR	[11]
	*Pterocephalus hookeri* (C.B.Clarke) E.Pritz.	Underground parts	Tibet	SER, PP, CC rp-CC, NMR	[58]
		n.a.	n.a.	n.a.	[222]
		Underground parts	Tibet	SER, PP, TLC, sp-HPLC-MS, NMR	[59]
		n.a.	n.a.	n.a.	[60]
		Whole plant	China	SE, PP, HPLC-UV	[62]
				USE, UPLC-MS^n^	[64]
	*Scabiosa atropurpurea* L.	Whole plant	Turkey	SE, CC, sp-HPLC-UV, HPLC-MS^n^	[34]
Sylvestroside III dimethyl acetal (Figure 30)	*Scaevola montana Labill.*	Aerial parts	New Caledonia	SE, CC, NMR	[43]
	*Pterocephalus hookeri* (C.B.Clarke) E.Pritz.	Underground parts	Tibet	SER, PP, CC rp-CC, NMR	[58]
		n.a.	n.a.	n.a.	[60]
		Underground parts	Tibet	SER, PP, TLC, sp-HPLC-MS, NMR	[59]
	*Scabiosa atropurpurea* L.	Whole plant	Turkey	SE, CC, sp-HPLC-UV, HPLC-MS^n^	[34]
Sylvestroside IV (Figure 30)	*Dipsacus fullonum* L.	Seeds	Denmark	SE, p-TLC, α_[D]_, UV, NMR	[41]
		Leaves	Estonia	DESE, HPLC-DAD-MS	[220]
		Leaves	Estonia	SE, CC, rp-FC, HPLC-DAD-MS, NMR	[221]
	*Dipsacus laciniatus* L.	Aerial parts	Hungary	SE, PP, CCD, CC, α_[D]_, IR, UV, NMR	[45]
	*Dipsacus ferox* Loisel.	Leaves and branches	Italy	SE, CC, NMR	[153]
	*Pterocephalus hookeri* (C.B.Clarke) E.Pritz.	Underground parts	Tibet	SER, PP, CC rp-CC, NMR	[58]
		Underground parts	Tibet	SER, PP, TLC, sp-HPLC-MS, NMR	[59]
		n.a.	n.a.	n.a.	[60]
		Whole plant	China	SE, PP, HPLC-UV	[62]
			Tibet	SE, PP, CC, sp-HPLC-UV, NMR	[24]
	*Scabiosa atropurpurea* L.	Whole plant	Turkey	SE, CC, sp-HPLC-UV, HPLC-MS^n^	[34]
Sylvestroside IV dimethyl acetal (Figure 30)	*Pterocephalus hookeri* (C.B.Clarke) E.Pritz.	Underground parts	Tibet	SER, PP, CC rp-CC, NMR	[58]
		Underground parts	Tibet	SER, PP, TLC, sp-HPLC-MS, NMR	[59]
		n.a.	n.a.	n.a.	[60]
		Whole plant	Tibet	SE, PP, CC, sp-HPLC-UV, NMR	[24]
	*Picrorhiza kurroa* Royle ex Benth.	Stems	Myanmar	USE, PP, CC, sp-HPLC-UV, NMR	[23]
	*Clinopodium serpyllifolium* subsp. *fruticosum* (L.) Bräuchler	Leaves	Palestine	DP, USE, UHPLC-DAD-MS^n^	[223]
Tricoloroside (Figure 9)	*Loasa tricolor* Ker Gawl.	Whole plant	Chile	SE, ACT, CC, MP, α_[D]_, IR, UV, NMR	[224]
Tricoloroside methyl ester (Figure 9)	*Loasa acerifolia* Dombey ex A.Juss.	Leaves	Germany (obtained from a botanical garden)	SXE, PP, CC, sp-HPLC-UV, α_[D]_, IR, UV, NMR, MS	[225]
Triplostoside A (Figure 9)	*Triplostegia glandulifera* Wall. ex DC.	Roots	n.a.	n.a.	[226]
	*Strychnos spinosa* Lam.	Branches	Japan (cultivated)	HSE, PP, rp-MPLC, p-HPLC-UV, p-TLC, NMR	[53]
	*Strychnos lucida* R.Br.	Bark and wood	Thailand	HSE, PP, MPLC, rp-MPLC, p-HPLC-UV, NMR	[54]
	*Dipsacus inermis* Wall.	Roots	China	HSE, PP, CC, rp-CC, p-TLC, rp-HPLC-UV, NMR	[48]
				HSE, PP, CC, p-TLC, p-rp-HPLC-UV, NMR	[106]
			China (purchased from a company)	SER, PP, MPLC, p-TLC, NMR	[50]
		n.a.	n.a.	n.a.	[219]
		Dried Roots	China (purchased from a company)	USE, HPLC-MS^n^	[51]
			n.a.	n.a.	[227]
			China (different populations)	SE, CC, UHPLC-PDA, UHPLC-MS^n^	[52]
			n.a.	n.a.	[228]
	*Strychnos axillaris* Colebr.	Bark and wood	Thailand	SER, PP, rp-MPLC, p-HPLC-UV, NMR	[36]
	*Pterocephalus hookeri* (C.B.Clarke) E.Pritz.	Whole plant	China	SE, PP, CC, rp-CC, NMR	[61]
				USE, UPLC-MS^n^	[64]
		n.a.	n.a.	n.a.	[222]
		n.a.	n.a.	n.a.	[229]
		n.a.	n.a.	n.a.	[60]
		Whole plant	Tibet	SE, PP, CC, p-HPLC-UV, p-TLC, NMR	[65]
	*Scabiosa semipapposa Salzm. ex DC.*	Aerial parts	Algeria	SE, rp-VLC, FC, rp-MPLC, NMR	[210]
Tripterospermumcin B methyl acetal (Figure 19)	*Tripterospermum chinense* (Migo) Harry Sm.	Aerial parts	China	SE, PP, CC, α_[D]_, IR, UV, NMR, HR-MS	[230]
				SER, PP, CC, p-HPLC-UV, NMR	[231]
Tripterospermumcin D (Figure 10)	*Tripterospermum chinense* (Migo) Harry Sm.	Aerial parts	China	SER, PP, CC, p-HPLC-UV, α_[D]_, IR, UV, NMR, HR-MS	[231]
Urceolatoside A (Figure 27)	*Viburnum urceolatum* Siebold & Zucc.	Leaves	Japan	SE, PP, CC, α_[D]_, MP, IR, UV, NMR	[232]
Urceolatoside B (Figure 27)	*Viburnum urceolatum* Siebold & Zucc.	Leaves	Japan	SE, PP, CC, α_[D]_, MP, IR, UV, NMR	[232]
Urceolatoside C (Figure 27)	*Viburnum urceolatum* Siebold & Zucc.	Leaves	Japan	SE, PP, CC, α_[D]_, MP, IR, UV, NMR	[232]
Valeridoid B (Figure 27)	*Valeriana jatamansi* Jones	Roots and rhizomes	China (purchased from a local market)	SE, PP, CC, p-TLC, α_[D]_, IR, UV, NMR, HR-MS	[233]
Valeridoid C (Figure 27)	*Valeriana jatamansi* Jones	Roots and rhizomes	China (purchased from a local market)	SE, PP, CC, sp-HPLC-UV, α_[D]_, IR, UV, NMR, HR-MS	[233]
Valeridoid D (Figure 27)	*Valeriana jatamansi* Jones	Roots and rhizomes	China (purchased from a local market)	SE, PP, CC, sp-HPLC-UV, α_[D]_, IR, UV, NMR, HR-MS	[233]
Valeridoid E (Figure 34)	*Valeriana jatamansi* Jones	Roots and rhizomes	China (purchased from a local market)	SE, PP, CC, sp-HPLC-UV, α_[D]_, IR, UV, NMR, HR-MS	[233]
Valeridoid F (Figure 34)	*Valeriana jatamansi* Jones	Roots and rhizomes	China (purchased from a local market)	SE, PP, CC, sp-HPLC-UV, α_[D]_, IR, UV, NMR, HR-MS	[233]
Wulfenoside (Figure 7)	*Wulfenia carinthiaca* Jacq.	Underground parts	Austria	SE, CC, HPLC-UV, α_[D]_, IR, UV, NMR, HR-MS	[234]
Dimer of alpinoside and alpinoside	*Globularia alypum* L.	Aerial parts	Croatia	SER, HPLC-PDA, HPLC-PDA-MS^n^	[127]
		Leaves	Croatia	USE, HPLC-PDA-MS^n^	[128]
Dimer of aperuloside and asperulosidic acid (Figure 3)	*Lasianthus attenuatus* var. *attenuatus*	Leaves	Japan	SE, PP, CC, HPLC-UV, α_[D]_, IR, UV, NMR, HR-MS	[235]
	*Lasianthus verticillatus* (Lour.) Merr.	Leaves	Japan	SE, PP, rp-CC, HPLC-UV, α_[D]_, IR, UV, NMR, MS	[152]
Dimer of nuezhenide and 11-methyl-oleoside	*Olea europaea* L.	Fruits	Tunisia (cultivated)	SE, HPLC-UV, UHPLC-MS^n^	[236]
Dimer of oleoside and 11-methyl-oleoside	*Olea europaea* L.	Fruits	Tunisia (cultivated)	SE, HPLC-UV, UHPLC-MS^n^	[236]
Dimer of paederosidic acid I (Figure 2)	*Paederia foetida* L.	Roots	Vietnam	SXE, PP, CC, rp-HPLC-UV, α_[D]_, IR, UV, NMR, MS	[237]
		Stems	China (purchased from a company)	SE, PP, HPLC-MS^n^	[178]
Dimer of paederosidic acid II (Figure 2)	*Paederia foetida* L.	Stems	China (purchased from a company)	SE, PP, HPLC-MS^n^	[178]
Dimer of paederosidic acid and asperuloside I (Figure 3)	*Paederia foetida* L.	Stems	China (purchased from a company)	SE, PP, CC, HPLC-MS^n^	[178]
Dimer of paederosidic acid and asperuloside II (Figure 3)	*Paederia foetida* L.	Stems	China (purchased from a company)	SE, PP, HPLC-MS^n^	[178]
Dimer of paederosidic acid and asperuloside III (Figure 3)	*Paederia foetida* L.	Stems	China	SE, PP, HPLC-MS^n^	[178]
			China (purchased from a company)	SE, PP, HPLC-MS^n^	[178]
Dimer of paederosidic acid and asperuloside IV (Figure 4)	*Paederia foetida* L.	Stems	China (purchased from a company)	SE, PP, HPLC-MS^n^	[178]
Dimer of paederosidic acid and paederoside (Figure 2)	*Paederia foetida* L.	Roots	Vietnam	SXE, PP, CC, rp-HPLC-UV, α_[D]_, IR, UV, NMR, MS	[237]
Dimer of paederosidic acid and paederosidic acid methyl ester (Figure 2)	*Paederia foetida* L.	Roots	Vietnam	SXE, PP, CC, rp-HPLC-UV, α_[D]_, IR, UV, NMR, MS	[237]
Iridoid glycoside dimer I (Figure 16)	*Jasminum azoricum* L.	Leaves	Egypt (obtained from a botanical garden)	HSE, PP, CC, α_[D]_, MP, IR, UV, NMR, MS	[238]

Legend: 2D-HPLC-UF-MS: bidimensional high-performance liquid chromatography coupled to ultrafiltration and mass spectrometry; α_[D]_: optical rotation; ACT: active charcoal treatment; CC; column chromatography; CCC: counter current chromatography; CCD: countercurrent distribution chromatography; CC-TLC: countercurrent thin-layer chromatography; CPC: centrifugal partition chromatography; DCCC: droplet countercurrent chromatography DESE: extraction by means of deep eutectic solvents; DP: Defatting procedure; ECD: electronic circular dichroism; FC: flash chromatography; HPLC-DAD: high-performance liquid chromatography coupled to diode array detector; HPLC-DAD-CL: high-performance liquid chromatography coupled to diode array detector and chemiluminescence detector; HPLC-DAD-ELSD: high-performance liquid chromatography coupled to diode array detector and evaporative light scattering detector; HPLC-DAD-MS: high-performance liquid chromatography coupled to diode array detector and mass spectrometry; HPLC-DAD-MS^n^: high-performance liquid chromatography coupled to diode array detector and tandem mass spectrometry; HPLC-ELSD: high-performance liquid chromatography coupled to evaporative light scattering detector; HPLC-MS: high-performance liquid chromatography coupled to mass spectrometry; HPLC-MS^n^: high-performance liquid chromatography coupled to tandem mass spectrometry; HPLC-PDA: high-performance liquid chromatography coupled to photo diode array spectroscopy; HPLC-PDA-MS^n^: high-performance liquid chromatography coupled to photo diode array spectroscopy and tandem mass spectrometry; HPLC-UV: high-performance liquid chromatography coupled to ultraviolet spectroscopy; HR-MS: high resolution mass spectrometry; HSE = hot solvent extraction by maceration; IR = infrared spectroscopy; LPLC: low pressure liquid chromatography; MP = melting point; MPLC: medium pressure liquid chromatography; MS: mass spectrometry; MS^n^: tandem mass spectrometry; n.a.: not accessible; NMR: nuclear magnetic resonance spectroscopy; PC: paper chromatography; p-HPLC-UV: preparative high-performance liquid chromatography coupled to ultraviolet spectroscopy; PP: partition procedure; p-rp-HPLC-UV: preparative reversed-phase high-performance liquid chromatography coupled to ultraviolet spectroscopy; p-TLC: preparative thin-layer chromatography; rp-CC: reversed-phase column chromatography; rp-FC: reversed-phase flash chromatography; rp-HPLC-DAD: reversed-phase high-performance liquid chromatography coupled to diode array detector; rp-HPLC-UV: reversed-phase high-performance liquid chromatography coupled to ultraviolet spectroscopy; rp-LPLC: reversed-phase low pressure liquid chromatography; rp-MPLC: reversed-phase medium pressure liquid chromatography; rp-UHPLC-PDA-MS^n^: reversed-phase ultra-high-performance liquid chromatography coupled to photo diode array spectroscopy and tandem mass spectrometry; rp-VLC: reversed-phase vacuum liquid chromatography; -: solvent extraction by maceration; -R: solvent extraction under reflux; SXE: extraction by Soxhlet; sp-HPLC-UV: semi-preparative high-performance liquid chromatography coupled to ultraviolet spectroscopy; sp-rp-HPLC-UV: semi-preparative reversed-phase high-performance liquid chromatography coupled to ultraviolet spectroscopy; TLC: thin-layer chromatography; UFLC-MS^n^: ultra-fast liquid chromatography coupled to tandem mass spectrometry; UHPLC-MS^n^: ultra-high-performance liquid chromatography coupled to tandem mass spectrometry; UHPLC-PDA: ultra-high-performance liquid chromatography coupled to photo diode array spectroscopy; UHPLC-PDA-MS^n^: ultra-high-performance liquid chromatography coupled to photo diode array spectroscopy and tandem mass spectrometry; UHPLC-PDA: ultra-performing liquid chromatography coupled to photo diode array spectroscopy; UHPLC-UV: ultra-performing liquid chromatography coupled to ultraviolet spectroscopy; UHPLC-PDA: ultra-performing liquid chromatography coupled to photo diode array spectroscopy; UHPLC-PDA-MS^n^: ultra-performing liquid chromatography coupled to photo diode array spectroscopy and tandem mass spectrometry; UPLC-HR-MS: ultra-performing liquid chromatography coupled to high resolution mass spectrometry; UPLC-MS: ultra-performing liquid chromatography coupled to mass spectrometry; UPLC-MS^n^: ultra-performing liquid chromatography coupled to tandem mass spectrometry; USE: extraction with ultrasound; UV: ultraviolet spectroscopy; VLC: vacuum liquid chromatography.

**Table 2:** Associated biological activities of all the identified *bis*-iridoids in plants.

Compound	Type of Biological Activity	Employed Methodology or Cells or Strains	Effectiveness Value	Positive Control with Effectiveness Value	Reference
(3*R*,5*S*)-5-carboxy-vincosidic acid 22-loganin ester	Anti-inflammatory	Inhibition of NO production in LPS-activated RAW264.7 macrophage cells	IC_50_ = 21.3 μM	L-NMMA (IC_50_ = 22.6 μM)	[108]
5-hydroxy-2‴-*O*-caffeoyl-caryocanoside B	Enzymatic	α-glucosidase	No effect	Acarbose (IC_50_ = 3.49 μM)	[10]
7-*O*-caffeoyl-sylvestroside I	Antioxidant	DPPH^·^	No effect	Ascorbic acid (IC_50_ = 6.3 μg/mL)	[12]
	Antibacterial	*Enterococcus faecalis* ATCC1054	MIC = 31.2 μg/mL	Gentamycin (MIC = 16 μg/mL)	
				Vancomycin (MIC > 64 μg/mL)	
		*Staphylococcus aureus* CIP53.154	MIC = 62.5 μg/mL	Gentamycin (MIC = 4 μg/mL)	
				Vancomycin (MIC > 64 μg/mL)	
		*Escherichia coli* CIP54.127	MIC = 250 μg/mL	Gentamycin (MIC = 4 μg/mL)	
				Vancomycin (MIC > 16 μg/mL)	
		*Staphylococcus epidermis*	MIC = 31.2 μg/mL	Gentamycin (MIC = 0.25 μg/mL)	
				Vancomycin (MIC = 4 μg/mL)	
		*Pseudomonas aeruginosa* ATCC9027	MIC = 125 μg/mL	Gentamycin (MIC = 8 μg/mL)	
				Vancomycin (MIC > 64 μg/mL)	
	Antitumoral	HT1080 (MTT assay)	IC_50_ = 35.9 μg/mL	Not reported	
	Enzymatic	Mushroom anti-tyrosinase	No effect	Kojic acid (IC_50_ = 6.8 μg/mL)	
7-*O*-(*p*-coumaroyl)-sylvestroside I	Antioxidant	DPPH^·^	No effect	Ascorbic acid (IC_50_ = 6.3 μg/mL)	[12]
	Antibacterial	*Enterococcus faecalis* ATCC1054	MIC = 31.2 μg/mL	Gentamycin (MIC = 16 μg/mL)	
				Vancomycin (MIC > 64 μg/mL)	
		*Staphylococcus aureus* CIP53.154	MIC = 62.5 μg/mL	Gentamycin (MIC = 4 μg/mL)	
				Vancomycin (MIC > 64 μg/mL)	
		*Escherichia coli* CIP54.127	MIC = 125 μg/mL	Gentamycin (MIC = 4 μg/mL)	
				Vancomycin (MIC > 16 μg/mL)	
		*Staphylococcus epidermis*	MIC = 31.2 μg/mL	Gentamycin (MIC = 0.25 μg/mL)	
				Vancomycin (MIC = 4 μg/mL)	
		*Pseudomonas aeruginosa* ATCC9027	MIC = 125 μg/mL	Gentamycin (MIC = 8 μg/mL)	
				Vancomycin (MIC > 64 μg/mL)	
	Antitumoral	HT1080 (MTT assay)	No effect	Not reported	
	Enzymatic	Mushroom anti-tyrosinase	No effect	Kojic acid (IC_50_ = 6.8 μg/mL)	
2‴-*O*-(*E*)-*p*-coumaroyl-caryocanoside B	Enzymatic	α-glucosidase	No effect	Acarbose (IC_50_ = 3.49 μM)	[10]
2‴-*O*-(*Z*)-*p*-coumaroyl-caryocanoside B	Enzymatic	α-glucosidase	IC_50_ = 0.38 μM	Acarbose (IC_50_ = 3.49 μM)	[10]
(*Z*)-aldosecologanin	Anti-inflammatory	Inhibition of NO production in LPS-stimulated RAW 264.7	IC_50_ = 7.96 μM	Mino (IC_50_ = 20.07 μM)	[15]
	Enzymatic	α-glucosidase	IC_50_ = 0.62 μM	Acarbose (IC_50_ = 4.32 μM)	
Abeliforoside C	Enzymatic	ATP-citrate lyase	No effect	BMS303141 (IC_50_ = 0.2 μM)	[21]
		Acetyl-CoA carboxylase	No effect	ND-630 (IC_50_ = 1.6 nM)	
Abeliforoside D	Enzymatic	ATP-citrate lyase	No effect	BMS303141 (IC_50_ = 0.2 μM)	[21]
		Acetyl-CoA carboxylase	No effect	ND-630 (IC_50_ = 1.6 nM)	
Abeliforoside E	Enzymatic	ATP-citrate lyase	No effect	BMS303141 (IC_50_ = 0.2 μM)	[21]
		Acetyl-CoA carboxylase	No effect	ND-630 (IC_50_ = 1.6 nM)	
Abeliforoside F	Enzymatic	ATP-citrate lyase	No effect	BMS303141 (IC_50_ = 0.2 μM)	[21]
		Acetyl-CoA carboxylase	No effect	ND-630 (IC_50_ = 1.6 nM)	
Abelioside A	Antiviral	Inhibition of the expression of Vpr in TREx-HeLa-Vpr cells	Cell proliferation % = 107% (at the concentration of 10 μM)	Damnacanthal (Cell proliferation % = 158% at the concentration of 10 μM)	[23]
Abelioside B	Antiviral	Inhibition of the expression of Vpr in TREx-HeLa-Vpr cells	Cell proliferation % = 129% (at the concentration of 10 μM)	Damnacanthal (Cell proliferation % = 158% at the concentration of 10 μM)	[23]
Abelioside A methyl acetal	Antitumoral	Caco2 (MTT assay)	IC_50_ = 5.49 μM	Paclitaxel (IC_50_ = 2.63 μM)	[24]
		Huh-7 (MTT assay)	IC_50_ = 8.49 μM	Paclitaxel (IC_50_ = 1.71 μM)	
		SW982 (MTT assay)	IC_50_ = 7.91 μM	Paclitaxel (IC_50_ = 1.99 μM)	
Asperulosidyl-2’b-*O*-paederoside	Anti-inflammatory	Inhibition of NO production in LPS-activated RAW264.7 macrophage cells	IC_50_ = 49.76 μM	Indomethacin (IC_50_ = 23.93 μM)	[108]
Atropurpurin A	Enzymatic	α-glucosidase from *Saccharomyces cerevisiae*	IC_50_ = 86.96 μM	Acarbose (IC_50_ = 175.00 μM)	[34]
Atropurpurin B	Enzymatic	α-glucosidase from *Saccharomyces cerevisiae*	IC_50_ = 92.59 μM	Acarbose (IC_50_ = 175.00 μM)	[34]
Blumeoside B	Antioxidant	Bleaching of the H_2_O-soluble carotenoid crocin	Low effect (value not reported)	Rutin (value not reported)	[37]
				Gallic acid (value not reported)	
		DPPH^·^	No effect	Quercetin (value not reported)	
				BHT (value not reported)	
Blumeoside D	Antioxidant	Bleaching of the H_2_O-soluble carotenoid crocin	Low effect (value not reported)	Rutin (value not reported)	[37]
			Similar effect (value not reported)	Gallic acid (value not reported)	
		DPPH^·^	No effect	Quercetin (value not reported)	
				BHT (value not reported)	
Cantleyoside	Antitumoral	Caco2 (MTT assay)	No effect	Paclitaxel (IC_50_ = 2.63 μM)	[24]
		Huh-7 (MTT assay)		Paclitaxel (IC_50_ = 1.71 μM)	
		SW982 (MTT assay)		Paclitaxel (IC_50_ = 1.99 μM)	
		A549 (MTT assay)		Florouracil (IC_50_ = 0.177 μg/mL)	[48]
		Bel7402 (MTT assay)		Florouracil (IC_50_ = 0.542 μg/mL)	
		BGC-823 (MTT assay)		Florouracil (IC_50_ = 0.695 μg/mL)	
		HCT-8 (MTT assay)		Florouracil (IC_50_ = 0.67 μg/mL)	
		A2780 (MTT assay)		Florouracil (IC_50_ = 0.569 μg/mL)	
		MCF-7 (MTT assay)	IC_50_ > 50 μM	Not reported	[61]
		HepG2 (MTT assay)			
		H460 (MTT assay)			
	Enzymatic	α-glucosidase from *Saccharomyces cerevisiae*	IC_50_ = 30.2 μM	Acarbose (IC_50_ = 175.00 μM)	[34]
	Neuroprotective	Aβ25–35 induced cell death in PC12 cells	Inhibition % = 23.17% (at the concentration of 10 μM)	Salvianolic acid B (Inhibition % = 18.28% at the concentration of 10 μM)	[49]
	Anti-inflammatory	Inhibition of NO production in LPS-activated RAW264.7 macrophage cells	IC_50_ > 50 μM	*L*-NMMA (IC_50_ = 22.6 μM)	[50]
			IC_50_ = 89.48 μM	*L*-NMMA (IC_50_ = 19.36 μM)	[65]
	Anti-arthritic	Inhibition of NO production in LPS-stimulated human rheumatoid arthritis fibroblast synovial cells	Good effect (values not reported)	Not reported	[115]
		Inhibition of TNF-α production in LPS-stimulated human rheumatoid arthritis fibroblast synovial cells			
		Inhibition of IL-1β/6 production in LPS-stimulated human rheumatoid arthritis fibroblast synovial cells			
Cantleyoside dimethyl acetal	Enzymatic	α-glucosidase from *Saccharomyces cerevisiae*	IC_50_ = 35.64 μM	Acarbose (IC_50_ = 175.00 μM)	[34]
	Antibacterial	*Staphylococcus aureus* ATCC25923	DIZ = 11 mm	Amoxicillin (DIZ = 21 mm)	[70]
				Clavulanic acid (DIZ = 22 mm)	
		*Staphylococcus epidermidis* ATCC12228	DIZ = 12 mm	Amoxicillin (DIZ = 21 mm)	
				Clavulanic acid (DIZ = 24 mm)	
		*Pseudomonas aeruginosa* ATCC27853	DIZ = 10 mm	Amoxicillin (DIZ = 25 mm)	
				Clavulanic acid (DIZ = 20 mm)	
		*Escherichia coli* ATCC25922	DIZ = 10 mm	Amoxicillin (DIZ = 22 mm)	
				Clavulanic acid (DIZ = 23 mm)	
		*Enterobacter cloacae* ATCC13047	DIZ = 8 mm	Amoxicillin (DIZ = 23 mm)	
				Clavulanic acid (DIZ = 25 mm)	
		*Klebsiella pneumoniae* ATCC13883	DIZ = 10 mm	Amoxicillin (DIZ = 24 mm)	
				Clavulanic acid (DIZ = 22 mm)	
	Antifungal	*Candida albicans* ATCC10231	DIZ = 9 mm	Amphotericin (DIZ = 23 mm)	
		*Candida tropicalis* ATCC13801	DIZ = 10 mm	Amphotericin (DIZ = 24 mm)	
		*Candida glabrata* ATCC28838	DIZ = 10 mm	Amphotericin (DIZ = 25 mm)	
Caryocanoside B	Enzymatic	α-glucosidase	No effect	Acarbose (IC_50_ = 3.49 μM)	[10]
Centauroside	Antioxidant	Peroxy-nitrite spiking test	No effect	Not reported	[81]
	Anti-inflammatory	Inhibition of NO production in LPS-stimulated RAW 264.7	IC_50_ = 12.6 μM	Mino (IC_50_ = 20.07 μM)	[15]
	Enzymatic	α-glucosidase	IC_50_ = 1.08 μM	Acarbose (IC_50_ = 4.32 μM)	
	Muscle contraction	Intestine tissue motility in mice	Relative frequency motility % = 98.4%	Loperamide hydrochloride (Relative frequency motility % = 82.7%	[89]
Centauroside A	Antitumoral	MCF-7	No effect	Carboplatin (IC_50_ = 17.5 μM)	[90]
		MDA-MB-453	No effect	Carboplatin (IC_50_ = 12.5 μM)	
		3T3-L1	IC_50_ = 152.7 μM	Carboplatin (IC_50_ = 16.1 μM)	
Chrysathain	Antitumoral	HL-60 (MTT assay)	IC_50_~70 μg/mL	Etoposide (IC_50_ not reported)	[91]
Citrifolinin A-1	Enzymatic	Inhibition of UVB-induced Transcriptional Activator Protein-1 activity	No effect	Not reported	[92]
Cocculoside	Antitumoral	A549	No effect	Adriamycin (value not reported)	[94]
		H157			
		HepG2			
		MCF-7			
	Enzymatic	Acetylcholinesterase	No effect	Tacrine (value not reported)	
Coptosapside A	Antibacterial	*Salmonella enterica* serovar (broth microdilution method)	No effect	Kanamycin (MIC = 0.39 mg/mL)	[96]
		*Typhimurium* UK-1 χ8956 (broth microdilution method)			
		*Pseudomonas aeruginosa* PA01 (broth microdilution method)			
		*Proteusbacillus vulgaris* CPCC160013 (broth microdilution method)			
		*Escherichia coli* CICC10003 (broth microdilution method)			
		*Mycobacterium smegmatis* mc2155 (broth microdilution method)			
		*Staphylococcus aureus* ATCC25923 (broth microdilution method)			
Coptosapside B	Antibacterial	*Salmonella enterica* serovar (broth microdilution method)	No effect	Kanamycin (MIC = 0.39 mg/mL)	[96]
		*Typhimurium* UK-1 χ8956 (broth microdilution method)			
		*Pseudomonas aeruginosa* PA01 (broth microdilution method)			
		*Proteusbacillus vulgaris* CPCC160013 (broth microdilution method)			
		*Escherichia coli* CICC10003 (broth microdilution method)			
		*Mycobacterium smegmatis* mc2155 (broth microdilution method)			
		*Staphylococcus aureus* ATCC25923 (broth microdilution method)			
Coptosapside C	Antibacterial	*Salmonella enterica* serovar (broth microdilution method)	No effect	Kanamycin (MIC = 0.39 mg/mL)	[96]
		*Typhimurium* UK-1 χ8956 (broth microdilution method)			
		*Pseudomonas aeruginosa* PA01 (broth microdilution method)			
		*Proteusbacillus vulgaris* CPCC160013 (broth microdilution method)			
		*Escherichia coli* CICC10003 (broth microdilution method)			
		*Mycobacterium smegmatis* mc2155 (broth microdilution method)			
		*Staphylococcus aureus* ATCC25923 (broth microdilution method)			
Coptosapside D	Antibacterial	*Salmonella enterica* serovar (broth microdilution method)	No effect	Kanamycin (MIC = 0.39 mg/mL)	[96]
		*Typhimurium* UK-1 χ8956 (broth microdilution method)			
		*Pseudomonas aeruginosa* PA01 (broth microdilution method)			
		*Proteusbacillus vulgaris* CPCC160013 (broth microdilution method)			
		*Escherichia coli* CICC10003 (broth microdilution method)			
		*Mycobacterium smegmatis* mc2155 (broth microdilution method)			
		*Staphylococcus aureus* ATCC25923 (broth microdilution method)			
Coptosapside E	Antibacterial	*Salmonella enterica* serovar (broth microdilution method)	No effect	Kanamycin (MIC = 0.39 mg/mL)	[96]
		*Typhimurium* UK-1 χ8956 (broth microdilution method)			
		*Pseudomonas aeruginosa* PA01 (broth microdilution method)			
		*Proteusbacillus vulgaris* CPCC160013 (broth microdilution method)			
		*Escherichia coli* CICC10003 (broth microdilution method)			
		*Mycobacterium smegmatis* mc2155 (broth microdilution method)			
		*Staphylococcus aureus* ATCC25923 (broth microdilution method)			
Coptosapside F	Antibacterial	*Salmonella enterica* serovar (broth microdilution method)	No effect	Kanamycin (MIC = 0.39 mg/mL)	[96]
		*Typhimurium* UK-1 χ8956 (broth microdilution method)			
		*Pseudomonas aeruginosa* PA01 (broth microdilution method)			
		*Proteusbacillus vulgaris* CPCC160013 (broth microdilution method)			
		*Escherichia coli* CICC10003 (broth microdilution method)			
		*Mycobacterium smegmatis* mc2155 (broth microdilution method)			
		*Staphylococcus aureus* ATCC25923 (broth microdilution method)			
Cornuofficinaliside C	Antidiabetic	Relative glucose consumption in insulin-induced HepG2 cells	Consumption = 0.624 mM/OD at the concentration of 10 μM	Rosiglitazone (1.33 (mM/OD at the concentration of 10 μM)	[97]
Cornuofficinaliside D	Antidiabetic	Relative glucose consumption in insulin-induced HepG2 cells	Consumption = 0.887 mM/OD at the concentration of 10 μM	Rosiglitazone (1.33 (mM/OD at the concentration of 10 μM)	[97]
Cornuofficinaliside E	Antidiabetic	Relative glucose consumption in insulin-induced HepG2 cells	Consumption = 0.595 mM/OD at the concentration of 10 μM	Rosiglitazone (1.33 (mM/OD at the concentration of 10 μM)	[97]
Cornuofficinaliside F	Antidiabetic	Relative glucose consumption in insulin-induced HepG2 cells	Consumption = 1.493 mM/OD at the concentration of 10 μM	Rosiglitazone (1.33 (mM/OD at the concentration of 10 μM)	[97]
Cornuofficinaliside G	Antidiabetic	Relative glucose consumption in insulin-induced HepG2 cells	Consumption = 0.841 mM/OD at the concentration of 10 μM	Rosiglitazone (1.33 (mM/OD at the concentration of 10 μM)	[97]
Cornuofficinaliside H	Antidiabetic	Relative glucose consumption in insulin-induced HepG2 cells	Consumption = 3.249 mM/OD at the concentration of 10 μM	Rosiglitazone (1.33 (mM/OD at the concentration of 10 μM)	[97]
Cornuofficinaliside I	Antidiabetic	Relative glucose consumption in insulin-induced HepG2 cells	Consumption = 0.704 mM/OD at the concentration of 10 μM	Rosiglitazone (1.33 (mM/OD at the concentration of 10 μM)	[97]
Cornuofficinaliside J	Antidiabetic	Relative glucose consumption in insulin-induced HepG2 cells	Consumption = 1.063 mM/OD at the concentration of 10 μM	Rosiglitazone (1.33 (mM/OD at the concentration of 10 μM)	[97]
Cornuofficinaliside K	Antidiabetic	Relative glucose consumption in insulin-induced HepG2 cells	Consumption = 0.716 mM/OD at the concentration of 10 μM	Rosiglitazone (1.33 (mM/OD at the concentration of 10 μM)	[97]
Cornuofficinaliside L	Antidiabetic	Relative glucose consumption in insulin-induced HepG2 cells	Consumption = 1.886 mM/OD at the concentration of 10 μM	Rosiglitazone (1.33 (mM/OD at the concentration of 10 μM)	[97]
Cornuofficinaliside M	Antidiabetic	Relative glucose consumption in insulin-induced HepG2 cells	Consumption = 0.652 mM/OD at the concentration of 10 μM	Rosiglitazone (1.33 (mM/OD at the concentration of 10 μM)	[97]
Cornusdiridoid A	Antidiabetic	Relative glucose consumption in insulin-induced HepG2 cells	EC_50_ = 15.31 μM	Rosiglitazone (EC_50_ = 3.35 μM)	[100]
	Antioxidant	DPPH^·^	No effect	Trolox (IC_50_ = 33.12 μM)	[98]
		ABTS^·+^	IC_50_ = 79.24 μM	Trolox (IC_50_ = 23.2 μM)	
	Enzymatic	*α*-glucosidase	IC_50_ = 243.5 μM	Acarbose (IC_50_ = 276.3 μM)	
	Anti-inflammatory	Inhibition of LPS-induced NO production in RAW 264.7 cells	IC_50_ = 28.87 μM	Indomethacin (IC_50_ = 48.32 μM)	
Cornusdiridoid B	Antioxidant	DPPH^·^	IC_50_ = 78.25 μM	Trolox (IC_50_ = 33.12 μM)	[98]
		ABTS^·+^	IC_50_ = 44.16 μM	Trolox (IC_50_ = 23.2 μM)	
	Enzymatic	*α*-glucosidase	IC_50_ = 251.9 μM	Acarbose (IC_50_ = 276.3 μM)	
	Anti-inflammatory	Inhibition of LPS-induced NO production in RAW 264.7 cells	IC_50_ = 29.52 μM	Indomethacin (IC_50_ = 48.32 μM)	
Cornusdiridoid C	Antioxidant	DPPH^·^	IC_50_ = 44.89 μM	Trolox (IC_50_ = 33.12 μM)	[98]
		ABTS^·+^	No effect	Trolox (IC_50_ = 23.2 μM)	
	Enzymatic	*α*-glucosidase	IC_50_ = 267.1 μM	Acarbose (IC_50_ = 276.3 μM)	
	Anti-inflammatory	Inhibition of LPS-induced NO production in RAW 264.7 cells	No effect	Indomethacin (IC_50_ = 48.32 μM)	
Cornusdiridoid D	Antioxidant	DPPH^·^	No effect	Trolox (IC_50_ = 33.12 μM)	[98]
		ABTS^·+^	IC_50_ = 48.99 μM	Trolox (IC_50_ = 23.2 μM)	
	Enzymatic	*α*-glucosidase	IC_50_ = 516.3 μM	Acarbose (IC_50_ = 276.3 μM)	
	Anti-inflammatory	Inhibition of LPS-induced NO production in RAW 264.7 cells	IC_50_ = 34.12 μM	Indomethacin (IC_50_ = 48.32 μM)	
Cornusdiridoid E	Antioxidant	DPPH^·^	IC_50_ = 36.60 μM	Trolox (IC_50_ = 33.12 μM)	[98]
		ABTS^·+^	IC_50_ = 48.99 μM	Trolox (IC_50_ = 23.2 μM)	
	Enzymatic	*α*-glucosidase	No effect	Acarbose (IC_50_ = 276.3 μM)	
	Anti-inflammatory	Inhibition of LPS-induced NO production in RAW 264.7 cells	No effect	Indomethacin (IC_50_ = 48.32 μM)	
Cornusdiridoid F	Antioxidant	DPPH^·^	IC_50_ = 60.17 μM	Trolox (IC_50_ = 33.12 μM)	[98]
		ABTS^·+^	IC_50_ = 17.10 μM	Trolox (IC_50_ = 23.2 μM)	
	Enzymatic	*α*-glucosidase	No effect	Acarbose (IC_50_ = 276.3 μM)	
	Anti-inflammatory	Inhibition of LPS-induced NO production in RAW 264.7 cells	IC_50_ = 26.84 μM	Indomethacin (IC_50_ = 48.32 μM)	
Cornuside A	Antidiabetic	Relative glucose consumption in insulin-induced HepG2 cells	No effect	Rosiglitazone (EC_50_ = 3.35 μM)	[100]
	Anti-inflammatory	Inhibition of the activation of IL-6-induced STAT3 in HepG2 cells	No effect	Genistein (IC_50_ = 24.8 μM)	[99]
Cornuside B	Anti-inflammatory	Inhibition of the activation of IL-6-induced STAT3 in HepG2 cells	No effect	Genistein (IC_50_ = 24.8 μM)	[99]
Cornuside C	Anti-inflammatory	Inhibition of the activation of IL-6-induced STAT3 in HepG2 cells	IC_50_ = 11.9 μM	Genistein (IC_50_ = 24.8 μM)	[99]
Cornuside D	Anti-inflammatory	Inhibition of the activation of IL-6-induced STAT3 in HepG2 cells	IC_50_ = 79.1 μM	Genistein (IC_50_ = 24.8 μM)	[99]
Cornuside E	Antidiabetic	Relative glucose consumption in insulin-induced HepG2 cells	No effect	Rosiglitazone (EC_50_ = 3.35 μM)	[100]
	Anti-inflammatory	Inhibition of the activation of IL-6-induced STAT3 in HepG2 cells	IC_50_ = 47.0 μM	Genistein (IC_50_ = 24.8 μM)	[99]
Cornuside F	Anti-inflammatory	Inhibition of the activation of IL-6-induced STAT3 in HepG2 cells	IC_50_ = 29.7 μM	Genistein (IC_50_ = 24.8 μM)	[99]
Cornuside G	Anti-inflammatory	Inhibition of the activation of IL-6-induced STAT3 in HepG2 cells	IC_50_ = 27.6 μM	Genistein (IC_50_ = 24.8 μM)	[99]
Cornuside H	Anti-inflammatory	Inhibition of the activation of IL-6-induced STAT3 in HepG2 cells	IC_50_ = 19.4 μM	Genistein (IC_50_ = 24.8 μM)	[99]
Cornuside I	Anti-inflammatory	Inhibition of the activation of IL-6-induced STAT3 in HepG2 cells	IC_50_ = 21.9 μM	Genistein (IC_50_ = 24.8 μM)	[99]
Cornuside J	Anti-inflammatory	Inhibition of the activation of IL-6-induced STAT3 in HepG2 cells	IC_50_ = 43.0 μM	Genistein (IC_50_ = 24.8 μM)	[99]
Cornuside K	Antidiabetic	Relative glucose consumption in insulin-induced HepG2 cells	EC_50_ = 70.43 μM	Rosiglitazone (EC_50_ = 3.35 μM)	[100]
	Anti-inflammatory	Inhibition of the activation of IL-6-induced STAT3 in HepG2 cells	No effect	Genistein (IC_50_ = 24.8 μM)	[99]
Cornuside L	Anti-inflammatory	Inhibition of the activation of IL-6-induced STAT3 in HepG2 cells	IC_50_ = 12.2 μM	Genistein (IC_50_ = 24.8 μM)	[99]
Cornuside M	Anti-inflammatory	Inhibition of the activation of IL-6-induced STAT3 in HepG2 cells	IC_50_ = 40.5 μM	Genistein (IC_50_ = 24.8 μM)	[99]
Cornuside N	Anti-inflammatory	Inhibition of the activation of IL-6-induced STAT3 in HepG2 cells	IC_50_ = 52.6 μM	Genistein (IC_50_ = 24.8 μM)	[99]
Cornuside O	Anti-inflammatory	Inhibition of the activation of IL-6-induced STAT3 in HepG2 cells	IC_50_ = 71.9 μM	Genistein (IC_50_ = 24.8 μM)	[99]
Demethyl-hydroxy-oleonuezhenide	Anti-inflammatory	Inhibition of CD11b expression in cytochalasin A and f-MLP stimulated neutrophils	Inhibition % = 1.5% (at the concentration of 50 μM)	Quercetin (No effect)	[103]
				Oleuropein (Inhibition % = 19.5% at the concentration of 50 μM)	
		Inhibition of ROS production in f-MLP stimulated neutrophils	Inhibition % = 59% (at the concentration of 50 μM)	Quercetin (Inhibition % = 93.2% at the concentration of 50 μM)	
				Oleuropein (Inhibition % = 73.7% at the concentration of 50 μM)	
		Inhibition of IL-8 expression in LPS stimulated macrophages	Inhibition % = 47.6% (at the concentration of 50 μM)	Quercetin (Inhibition % = 78.3% at the concentration of 50 μM)	
				Oleuropein (Inhibition % = 13.5% at the concentration of 50 μM)	
		Inhibition of IL-10 expression in LPS stimulated macrophages	No effect	Oleuropein (Induction % = +172% at the concentration of 50 μM)	
		Inhibition of TNF-α expression in LPS stimulated macrophages	Inhibition % = 38.1% (at the concentration of 50 μM)	Quercetin (Inhibition % = 91.1% at the concentration of 50 μM)	
				Oleuropein (Inhibition % = 71.7% at the concentration of 50 μM)	
Demethyl-oleonuezhenide	Anti-inflammatory	Inhibition of CD11b expression in cytochalasin A and f-MLP stimulated neutrophils	No effect	Quercetin (No effect)	[103]
				Oleuropein (Inhibition % = 19.5% at the concentration of 50 μM)	
		Inhibition of ROS production in f-MLP stimulated neutrophils	Inhibition % = 44.4% (at the concentration of 50 μM)	Quercetin (Inhibition % = 93.2% at the concentration of 50 μM)	
				Oleuropein (Inhibition % = 73.7% at the concentration of 50 μM)	
		Inhibition of IL-8 expression in LPS stimulated macrophages	Inhibition % = 62.3% (at the concentration of 50 μM)	Quercetin (Inhibition % = 78.3% at the concentration of 50 μM)	
				Oleuropein (Inhibition % = 13.5% at the concentration of 50 μM)	
		Inhibition of IL-10 expression in LPS stimulated macrophages	Induction % = +65.4% (at the concentration of 50 μM)	Oleuropein (Induction % = +172% at the concentration of 50 μM)	
		Inhibition of TNF-α expression in LPS stimulated macrophages	Inhibition % = 16.2% (at the concentration of 50 μM)	Quercetin (Inhibition % = 91.1% at the concentration of 50 μM)	
				Oleuropein (Inhibition % = 71.7% at the concentration of 50 μM)	
Dioscoridin C	Antitumoral	HeLa (MTT assay)	Inhibition % = 12.23% (at the concentration of 30 μM)	Cisplatin (Inhibition % = 99.93% at the concentration of 30 μM)	[105]
		A2780 (MTT assay)	Inhibition % = 12.29% (at the concentration of 30 μM)	Cisplatin (Inhibition % = 95.02% at the concentration of 30 μM)	[105]
		T47D (MTT assay)	Inhibition % = 33.42% (at the concentration of 30 μM)	Cisplatin (Inhibition % = 57.95% at the concentration of 30 μM)	[105]
Dipsanoside C	Antitumoral	A549 (MTT assay)	No effect	Florouracil (IC_50_ = 0.177 μg/mL)	[48]
		Bel7402 (MTT assay)		Florouracil (IC_50_ = 0.542 μg/mL)	
		BGC-823 (MTT assay)		Florouracil (IC_50_ = 0.695 μg/mL)	
		HCT-8 (MTT assay)		Florouracil (IC_50_ = 0.67 μg/mL)	
		A2780 (MTT assay)		Florouracil (IC_50_ = 0.569 μg/mL)	
Dipsanoside D	Antitumoral	A549 (MTT assay)	No effect	Florouracil (IC_50_ = 0.177 μg/mL)	[48]
		Bel7402 (MTT assay)		Florouracil (IC_50_ = 0.542 μg/mL)	
		BGC-823 (MTT assay)		Florouracil (IC_50_ = 0.695 μg/mL)	
		HCT-8 (MTT assay)		Florouracil (IC_50_ = 0.67 μg/mL)	
		A2780 (MTT assay)		Florouracil (IC_50_ = 0.569 μg/mL)	
Dipsanoside E	Antitumoral	A549 (MTT assay)	No effect	Florouracil (IC_50_ = 0.177 μg/mL)	[48]
		Bel7402 (MTT assay)		Florouracil (IC_50_ = 0.542 μg/mL)	
		BGC-823 (MTT assay)		Florouracil (IC_50_ = 0.695 μg/mL)	
		HCT-8 (MTT assay)		Florouracil (IC_50_ = 0.67 μg/mL)	
		A2780 (MTT assay)		Florouracil (IC_50_ = 0.569 μg/mL)	
Dipsanoside F	Antitumoral	A549 (MTT assay)	No effect	Florouracil (IC_50_ = 0.177 μg/mL)	[48]
		Bel7402 (MTT assay)		Florouracil (IC_50_ = 0.542 μg/mL)	
		BGC-823 (MTT assay)		Florouracil (IC_50_ = 0.695 μg/mL)	
		HCT-8 (MTT assay)		Florouracil (IC_50_ = 0.67 μg/mL)	
		A2780 (MTT assay)		Florouracil (IC_50_ = 0.569 μg/mL)	
Dipsanoside G	Antitumoral	A549 (MTT assay)	No effect	Florouracil (IC_50_ = 0.177 μg/mL)	[48]
		Bel7402 (MTT assay)		Florouracil (IC_50_ = 0.542 μg/mL)	
		BGC-823 (MTT assay)		Florouracil (IC_50_ = 0.695 μg/mL)	
		HCT-8 (MTT assay)		Florouracil (IC_50_ = 0.67 μg/mL)	
		A2780 (MTT assay)		Florouracil (IC_50_ = 0.569 μg/mL)	
Dipsanoside J	Anti-inflammatory	Inhibition of LPS-induced NO production in RAW264.7 macrophages	No effect	Not reported	[106]
Dipsanoside M	Antiviral	HIV-1 integrase inhibition activities (microplate screening assay)	IC_50_ = 84.03 μM	Baicalein (IC_50_ = 1.37 μM)	[107]
Dipsanoside N	Antiviral	HIV-1 integrase inhibition activities (microplate screening assay)	IC_50_ = 92.67 μM	Baicalein (IC_50_ = 1.37 μM)	[107]
Dipasaperine	Antitumoral	A549	No effect	Adriamycin (value not reported)	[94]
		H157			
		HepG2			
		MCF-7			
	Enzymatic	Acetylcholinesterase	No effect	Tacrine (value not reported)	
	Anti-inflammatory	Inhibition of NO production in LPS-activated RAW264.7 macrophage cells	IC_50_ = 20.5 μM	L-NMMA (IC_50_ = 22.6 μM)	[108]
Disperoside A	Enzymatic	A-glucosidase	IC_50_ > 50 μM	Not reported	[109]
Disperoside B	Enzymatic	A-glucosidase	IC_50_ > 50 μM	Not reported	[109]
GI-3	Enzymatic	MMP-2	IC_50_ < 100 μM	Doxycycline (IC_50_ > 100 μM)	[122]
		MMP-9	IC_50_ < 100 μM		
	Immunosupressive	Inhibition of IL-2 production in T activated cells after treatment with PMA	No effect	Not reported	[121]
	Weight losing	Adipogenesis inhibition	Inhibition % = 2.1% (at the concentration of 1 mg/mL)	Not reported	[115]
		Activation of PPARα-mediated pathways	Activation % = 21.0% (at the concentration of 10^−4^ M)	WY14,643 (Activation % = 100% at the concentration of 10^−5^ M)	
		GTS inhibition in 3T3-L1 preadipocytes	No effect	Not reported	
	Pain killing	Induction of ERK and CREB phosphorylation in primary cortical neuron	No effect	Not reported	[116]
GI-5	Weight losing	Adipogenesis inhibition	Inhibition % = 100% (at the concentration of 1 mg/mL)	Not reported	[115]
		Activation of PPARα-mediated pathways	Activation % = 14.2% (at the concentration of 10^−4^ M)	WY14,643 (Activation % = 100% at the concentration of 10^−5^ M)	
		GTS inhibition in 3T3-L1 preadipocytes	No effect	Not reported	
Hydroxy-oleonuezhenide	Anti-inflammatory	Inhibition of CD11b expression in cytochalasin A and f-MLP stimulated neutrophils	Inhibition % = 12.8% (at the concentration of 50 μM)	Quercetin (No effect)	[103]
				Oleuropein (Inhibition % = 19.5% at the concentration of 50 μM)	
		Inhibition of ROS production in f-MLP stimulated neutrophils	Inhibition % = 59% (at the concentration of 50 μM)	Quercetin (Inhibition % = 93.2% at the concentration of 50 μM)	
				Oleuropein (Inhibition % = 73.7% at the concentration of 50 μM)	
		Inhibition of IL-8 expression in LPS stimulated macrophages	Inhibition % = 48.6% (at the concentration of 50 μM)	Quercetin (Inhibition % = 78.3% at the concentration of 50 μM)	
				Oleuropein (Inhibition % = 13.5% at the concentration of 50 μM)	
		Inhibition of IL-10 expression in LPS stimulated macrophages	Induction % = +58.9% (at the concentration of 50 μM)	Oleuropein (Induction % = +172% at the concentration of 50 μM)	
		Inhibition of TNF-α expression in LPS stimulated macrophages	Inhibition % = 11.8% (at the concentration of 50 μM)	Quercetin (Inhibition % = 91.1% at the concentration of 50 μM)	
				Oleuropein (Inhibition % = 71.7% at the concentration of 50 μM)	
Hookerinoid A	Anti-inflammatory	Inhibition of NF-kB pathway in a luciferase reporter gene	LC_50_ = 18 mM	Not reported	[130]
Hookerinoid B	Anti-inflammatory	Inhibition of NF-kB pathway in a luciferase reporter gene	LC_50_ = 16 mM	Not reported	[130]
*Iso*-jaspolyoside A	Antioxidant	DPPH^·^	EC_50_ = 100 μg/mL	BHT (EC_50_ = 111 μg/mL)	[135]
*Iso*-oleonuzhenide	Pain killing	Induction of ERK and CREB phosphorylation in primary cortical neuron	Not reported	Not reported	[116]
	Immunosupressive	Inhibition of IL-2 production in T activated cells after treatment with PMA	No effect	Not reported	[121]
Jasmigeniposide B	Antiviral	H1N1	No effect	Not reported	[138]
		H3N2			
		EV-71			
Japonicoside E	Anti-inflammatory	Inhibition of PGE2 in LPS-stimulated Raw 246.7 cells	No effect	Not reported	[137]
Jasnervoside F	Antioxidant	DPPH^·^	Inhibition % = 28.31% (at the concentration of 5 μg/mL)	Ascorbic acid (IC_50_ = 0.88 μg/mL)	[139]
	Anti-inflammatory	Inhibition of NO production in LPS-treated BV2 cells	Inhibition % = 43.15% (at the concentration of 10 μM)	Curcumin (Inhibition % = 41.78% at the concentration of 1 μM)	
		Inhibition of TNF-α production in LPS-treated BV2 cells	Inhibition % = 13.8% (at the concentration of 10 μM)	Curcumin (Inhibition % = 60.37% at the concentration of 1 μM)	
		Inhibition of IL-1b production in LPS-treated BV2 cells	Inhibition % = 23.35% (at the concentration of 10 μM)	Curcumin (Inhibition % = 46.67% at the concentration of 1 μM)	
	Antitumoral	A-549	No effect	Florouracil (value not reported)	
		HC-T8			
		BEL-7402			
Jaspolyanoside	Antioxidant	DPPH^·^	EC_50_ = 711 μg/mL	BHT (EC_50_ = 111 μg/mL)	[135]
	Neuroprotection	NGF secretion in C6 cells	Secretion % = 114.4% (at the concentration of 50 μg/mL)	6-shogaol (Secretion % = 168.58%)	[142]
Jaspolyoside	Antioxidant	DPPH^·^	EC_50_ = 51 μg/mL	BHT (EC_50_ = 111 μg/mL)	[135]
			No effect	BHA (EC_50_ = 26.46 μg/mL)	[144]
		Superoxide anion	EC_50_ = 4.97 μM	BHA (EC_50_ = 16.5 μg/mL)	[144]
	Neuroprotection	NGF secretion in C6 cells	Secretion % = 171.64 % (at the concentration of 50 μg/mL)	6-shogaol (Secretion % = 168.58%)	[142]
Korolkoside	Toxicity	Mice	Not lethal but weakening (LD_50_ not calculated)	Not reported	[149]
Laciniatoside I	Antibacterial	*Staphylococcus aureus*	MIC = 64 μg/mL	Gentamycin (MIC = 1 μg/mL)	[151]
		*Staphylococcus epidermidis*	MIC = 32 μg/mL		
		*Salmonella typhimurium*	MIC = 64 μg/mL		
		*Escherichia coli*	MIC = 16 μg/mL		
		*Bacillus cereus*	MIC = 16 μg/mL	Gentamycin (MIC = 4 μg/mL)	
		*Klebsiella pneumoniae*	MIC = 32 μg/mL		
		*Enterococcus faecalis*	MIC = 16 μg/mL	Gentamycin (MIC = 16 μg/mL)	
		*Pseudomonas aeruginosa*	MIC = 16 μg/mL	Gentamycin (MIC = 2 μg/mL)	
Laciniatoside II	Antitumoral	Caco2 (MTT assay)	No effect	Paclitaxel (IC_50_ = 2.63 μM)	[24]
		Huh-7 (MTT assay)		Paclitaxel (IC_50_ = 1.71 μM)	
		SW982 (MTT assay)		Paclitaxel (IC_50_ = 1.99 μM)	
Laciniatoside V	Enzymatic	α-glucosidase from *Saccharomyces cerevisiae*	IC_50_ = 25.01 μM	Acarbose (IC_50_ = 175.00 μM)	[34]
Lisianthoside	Toxicity	Brine shrimp	LC_50_ = 150 ppm	Not reported	[160]
	Antifungal	*Cladosporium cucumcvinum*	No effect	Propiconazole (MIC = 1 μg/mL)	[209]
	Antitumoral	A549 (MTT assay)	No effect	Florouracil (IC_50_ = 0.177 μg/mL)	[48]
		Bel7402 (MTT assay)		Florouracil (IC_50_ = 0.542 μg/mL)	
		BGC-823 (MTT assay)		Florouracil (IC_50_ = 0.695 μg/mL)	
		HCT-8 (MTT assay)		Florouracil (IC_50_ = 0.67 μg/mL)	
		A2780 (MTT assay)		Florouracil (IC_50_ = 0.569 μg/mL)	
Minutifloroside	Antioxidant	DPPH^·^	Not reported	Not reported	[163]
	Antifungal	*Candida albicans* ATCC90028	MIC = 9.765 μg/mL	Fluconazole (MIC not reported)	
		*Candida glabrata* ATCC90030	MIC = 1250 μg/mL		
*Neo*-cornuside C	Antidiabetic	Relative glucose consumption in insulin-induced HepG2 cells	EC_50_ = 1.275 μM	Rosiglitazone (EC_50_ = 1.127 μM)	[167]
*Neo*-cornuside D	Antidiabetic	Relative glucose consumption in insulin-induced HepG2 cells	No effect	Rosiglitazone (EC_50_ = 1.127 μM)	[167]
*Neo*-cornuside F	Antidiabetic	Relative glucose consumption in insulin-induced HepG2 cells	EC_50_ = 40.12 μM	Rosiglitazone (EC_50_ = 3.35 μM)	[167]
Officinaloside A	Antibacterial	*Bacillus cereus*	MIC = 25 μg/mL	Ampicillin (MIC = 6.25 μg/mL)	[169]
		*Bacillus subtilis*	MIC = 12.5 μg/mL		
		*Staphylococcus aureus*	MIC = 50 μg/mL	Ampicillin (MIC = 12.5 μg/mL)	
		*Escherichia coli*	No effect	Ampicillin (No effect)	
Oleonuezhenide	Anti-inflammatory	Inhibition of CD11b expression in cytochalasin A and f-MLP stimulated neutrophils	Inhibition % = 2% (at the concentration of 50 μM)	Quercetin (No effect)	[103]
				Oleuropein (Inhibition % = 19.5% at the concentration of 50 μM)	
		Inhibition of ROS production in f-MLP stimulated neutrophils	Inhibition % = 42.4% (at the concentration of 50 μM)	Quercetin (Inhibition % = 93.2% at the concentration of 50 μM)	
				Oleuropein (Inhibition % = 73.7% at the concentration of 50 μM)	
		Inhibition of IL-8 expression in LPS stimulated macrophages	Inhibition % = 40% (at the concentration of 50 μM)	Quercetin (Inhibition % = 78.3% at the concentration of 50 μM)	
				Oleuropein (Inhibition % = 13.5% at the concentration of 50 μM)	
		Induction of IL-10 expression in LPS stimulated macrophages	Induction % = +89.6% (at the concentration of 50 μM)	Oleuropein (Induction % = +172% at the concentration of 50 μM)	
		Inhibition of TNF-α expression in LPS stimulated macrophages	Inhibition % = 10.9% (at the concentration of 50 μM)	Quercetin (Inhibition % = 91.1% at the concentration of 50 μM)	
				Oleuropein (Inhibition % = 71.7% at the concentration of 50 μM)	
	Enzymatic	MMP-2	IC_50_ < 100 μM	Doxycycline (IC_50_ > 100 μM)	[122]
		MMP-9	IC_50_ < 100 μM		
	Pain killing	Induction of ERK and CREB phosphorylation in primary cortical neuron	No effect	Not reported	[116]
	Neuroptrection	6-OHDA-induced in SH-SY5Y cells	Relative protection % = 42.8 (at the concentration of 10 μg/mL)	EGGG (Relative protection % = 72.0 at the concentration of 10 μg/mL)	[172]
		NGF secretion in C6 cells	Secretion % = 72.39% (at the concentration of 50 μg/mL)	6-shogaol (Secretion % = 168.58%)	[142]
	Osteogenic	MC3T3-E1 proliferation	Proliferation % = 10% (at the concentration of 5 μM)	Alendronate sodium (cell proliferation % = 5% at the concentration of 5 μM)	[175]
		ALP in MC3T3-E1 cells	Activity % = +25% (at the concentration of 5 μM)	Alendronate sodium (activity % = +10% (at the concentration of 5 μM)	
Paederoscandoside	Anti-inflammatory	Inhibition of NO production in LPS-activated RAW264.7 macrophage cells	IC_50_ = 37.41 μM	Indomethacin (IC_50_ = 23.93 μM)	[108]
Patriscabiobisin A	Antitumoral	HL-60 (MTT assay)	IC_50_ = 17.9 μM	Cisplatin (IC_50_ = 2.8 μM)	[181]
				Paclitaxel (IC_50_ < 0.008 μM)	
		SMMC-7721 (MTT assay)	IC_50_ = 19.7 μM	Cisplatin (IC_50_ = 5.9 μM)	
				Paclitaxel (IC_50_ < 0.008 μM)	
		MCF-7 (MTT assay)	IC_50_ = 23.9 μM	Cisplatin (IC_50_ = 20.4 μM)	
				Paclitaxel (IC_50_ < 0.008 μM)	
		SW-480 (MTT assay)	IC_50_ = 17.6 μM	Cisplatin (IC_50_ = 7.6 μM)	
				Paclitaxel (IC_50_ < 0.008 μM)	
	Enzymatic	Acetylcholinesterase	Inhibitory % = 36.03% (at the concentration of 50 μM)	Tacrine (Inhibitory % = 51.01% at the concentration of 0.4 μM)	
Patriscabiobisin B	Antitumoral	HL-60 (MTT assay)	No effect	Cisplatin (IC_50_ = 2.8 μM) Paclitaxel (IC_50_ < 0.008 μM)	[181]
		SMMC-7721 (MTT assay)		Cisplatin (IC_50_ = 5.9 μM) Paclitaxel (IC_50_ < 0.008 μM)	
		MCF-7 (MTT assay)		Cisplatin (IC_50_ = 20.4 μM) Paclitaxel (IC_50_ < 0.008 μM)	
		SW-480 (MTT assay)		Cisplatin (IC_50_ = 7.6 μM) Paclitaxel (IC_50_ < 0.008 μM)	
	Enzymatic	Acetylcholinesterase	Inhibitory % = 21.91% (at the concentration of 50 μM)	Tacrine (Inhibitory % = 51.01% at the concentration of 0.4 μM)	
Patriscabiobisin C	Antitumoral	HL-60 (MTT assay)	No effect	Cisplatin (IC_50_ = 2.8 μM)	[181]
				Paclitaxel (IC_50_ < 0.008 μM)	
		HL-60		Not reported	[182]
		SMMC-7721 (MTT assay)	No effect	Cisplatin (IC_50_ = 5.9 μM)	[181]
				Paclitaxel (IC_50_ < 0.008 μM)	
		SMMC-7721		Not reported	[182]
		MCF-7 (MTT assay)	No effect	Cisplatin (IC_50_ = 20.4 μM)	[181]
				Paclitaxel (IC_50_ < 0.008 μM)	
		MCF-7		Not reported	[182]
		SW-480 (MTT assay)	No effect	Cisplatin (IC_50_ = 7.6 μM)	[181]
				Paclitaxel (IC_50_ < 0.008 μM)	
		SW-480	No effect	Not reported	[182]
	Enzymatic	Acetylcholinesterase	Inhibitory % = 37.87% (at the concentration of 50 μM)	Tacrine (Inhibitory % = 51.01% at the concentration of 0.4 μM)	[181]
Phukettoside A	Antioxidant	DPPH·	No effect	Ascorbic acid (IC_50_ = 32.2 μM)	[183]
		Xanthine oxidase		Allopurinol (IC_50_ = 4.6 μM)	
		HL-60 antioxidant		Superoxide dismutase (Inhibition % = 100% at the dose of 60 U/mL)	
		LOX		*Nor*-dihydro-guaiaretic acid (IC_50_ = 4.5 μM)	
		Aromatase		Letrozole (IC_50_ = 1.4 nM)	
		Superoxide anion radical formation (XXO assay)		Gallic acid (IC_50_ = 2.9 μM)	
	Antitumoral	HuCCA-1 (MTT assay)	No effect	Doxorubicin (IC_50_ = 0.79 μM)	
		A549 (MTT assay)		Doxorubicin (IC_50_ = 0.19 μM)	
		HeLa (MTT assay)		Doxorubicin (IC_50_ = 0.16 μM)	
		HepG2 (MTT assay)		Doxorubicin (IC_50_ = 0.33 μM)	
		MRC-5 (MTT assay)		Doxorubicin (IC_50_ = 1.31 μM)	
		MDA-MB-231		Doxorubicin (IC_50_ = 1.18 μM)	
		MOLT-3		Etoposide (IC_50_ = 0.018 μM)	
Phukettoside B	Antioxidant	DPPH·	No effect	Ascorbic acid (IC_50_ = 32.2 μM)	[183]
		Xanthine oxidase		Allopurinol (IC_50_ = 4.6 μM)	
		HL-60 antioxidant		Superoxide dismutase (Inhibition % = 100% at the dose of 60 U/mL)	
		LOX		*Nor*-dihydro-guaiaretic acid (IC_50_ = 4.5 μM)	
		Aromatase		Letrozole (IC_50_ = 1.4 nM)	
		Superoxide anion radical formation (XXO assay)		Gallic acid (IC_50_ = 2.9 μM)	
	Antitumoral	HuCCA-1 (MTT assay)	No effect	Doxorubicin (IC_50_ = 0.79 μM)	
		A549 (MTT assay)		Doxorubicin (IC_50_ = 0.19 μM)	
		HeLa (MTT assay)		Doxorubicin (IC_50_ = 0.16 μM)	
		HepG2 (MTT assay)		Doxorubicin (IC_50_ = 0.33 μM)	
		MRC-5 (MTT assay)		Doxorubicin (IC_50_ = 1.31 μM)	
		MDA-MB-231		Doxorubicin (IC_50_ = 1.18 μM)	
		MOLT-3		Etoposide (IC_50_ = 0.018 μM)	
Phukettoside C	Antioxidant	DPPH·	No effect	Ascorbic acid (IC_50_ = 32.2 μM)	[183]
		Xanthine oxidase		Allopurinol (IC_50_ = 4.6 μM)	
		HL-60 antioxidant		Superoxide dismutase (Inhibition % = 100% at the dose of 60 U/mL)	
		LOX		*Nor*-dihydro-guaiaretic acid (IC_50_ = 4.5 μM)	
		Aromatase		Letrozole (IC_50_ = 1.4 nM)	
		Superoxide anion radical formation (XXO assay)		Gallic acid (IC_50_ = 2.9 μM)	
	Antitumoral	HuCCA-1 (MTT assay)	No effect	Doxorubicin (IC_50_ = 0.79 μM)	
		A549 (MTT assay)		Doxorubicin (IC_50_ = 0.19 μM)	
		HeLa (MTT assay)		Doxorubicin (IC_50_ = 0.16 μM)	
		HepG2 (MTT assay)		Doxorubicin (IC_50_ = 0.33 μM)	
		MRC-5 (MTT assay)		Doxorubicin (IC_50_ = 1.31 μM)	
		MDA-MB-231		Doxorubicin (IC_50_ = 1.18 μM)	
		MOLT-3		Etoposide (IC_50_ = 0.018 μM)	
Phukettoside D	Antioxidant	DPPH·	No effect	Ascorbic acid (IC_50_ = 32.2 μM)	[183]
		Xanthine oxidase		Allopurinol (IC_50_ = 4.6 μM)	
		HL-60 antioxidant		Superoxide dismutase (Inhibition % = 100% at the dose of 60 U/mL)	
		LOX		*Nor*-dihydro-guaiaretic acid (IC_50_ = 4.5 μM)	
		Aromatase		Letrozole (IC_50_ = 1.4 nM)	
		Superoxide anion radical formation (XXO assay)		Gallic acid (IC_50_ = 2.9 μM)	
	Antitumoral	HuCCA-1 (MTT assay)	No effect	Doxorubicin (IC_50_ = 0.79 μM)	
		A549 (MTT assay)		Doxorubicin (IC_50_ = 0.19 μM)	
		HeLa (MTT assay)		Doxorubicin (IC_50_ = 0.16 μM)	
		HepG2 (MTT assay)		Doxorubicin (IC_50_ = 0.33 μM)	
		MRC-5 (MTT assay)		Doxorubicin (IC_50_ = 1.31 μM)	
		MDA-MB-231		Doxorubicin (IC_50_ = 1.18 μM)	
		MOLT-3		Etoposide (IC_50_ = 0.018 μM)	
Picconioside I	Enzymatic	A-glucosidase	Inhibition % = 63.8%	Acarbose (Inhibition % = 95.1%)	[185]
Picrorhizaoside E	Enzymatic	Hyaluronidase	IC_50_ = 35.8 μg/mL	Disodium cromoglycate (IC_50_ = 64.8 μg/mL)	[186]
				Ketotifen fumarate (IC_50_ = 76.5 μg/mL)	
				Tranilast (IC_50_ = 227 μg/mL)	
Picrorhizaoside F	Enzymatic	Hyaluronidase	No effect	Disodium cromoglycate (IC_50_ = 64.8 μg/mL)	[186]
				Ketotifen fumarate (IC_50_ = 76.5 μg/mL)	
				Tranilast (IC_50_ = 227 μg/mL)	
Picrorhizaoside G	Enzymatic	Hyaluronidase	No effect	Disodium cromoglycate (IC_50_ = 64.8 μg/mL)	[186]
				Ketotifen fumarate (IC_50_ = 76.5 μg/mL)	
				Tranilast (IC_50_ = 227 μg/mL)	
Ptehoside C	Antitumoral	Caco2 (MTT assay)	No effect	Paclitaxel (IC_50_ = 2.63 μM)	[24]
		Huh-7 (MTT assay)		Paclitaxel (IC_50_ = 1.71 μM)	
		SW982 (MTT assay)		Paclitaxel (IC_50_ = 1.99 μM)	
Ptehoside D	Antitumoral	Caco2 (MTT assay)	No effect	Paclitaxel (IC_50_ = 2.63 μM)	[24]
		Huh-7 (MTT assay)		Paclitaxel (IC_50_ = 1.71 μM)	
		SW982 (MTT assay)		Paclitaxel (IC_50_ = 1.99 μM)	
Ptehoside E	Antitumoral	Caco2 (MTT assay)	No effect	Paclitaxel (IC_50_ = 2.63 μM)	[24]
		Huh-7 (MTT assay)		Paclitaxel (IC_50_ = 1.71 μM)	
		SW982 (MTT assay)		Paclitaxel (IC_50_ = 1.99 μM)	
Ptehoside F	Antitumoral	Caco2 (MTT assay)	No effect	Paclitaxel (IC_50_ = 2.63 μM)	[24]
		Huh-7 (MTT assay)		Paclitaxel (IC_50_ = 1.71 μM)	
		SW982 (MTT assay)		Paclitaxel (IC_50_ = 1.99 μM)	
Ptehoside G	Antitumoral	Caco2 (MTT assay)	No effect	Paclitaxel (IC_50_ = 2.63 μM)	[24]
		Huh-7 (MTT assay)		Paclitaxel (IC_50_ = 1.71 μM)	
		SW982 (MTT assay)		Paclitaxel (IC_50_ = 1.99 μM)	
Ptehoside H	Antitumoral	Caco2 (MTT assay)	No effect	Paclitaxel (IC_50_ = 2.63 μM)	[24]
		Huh-7 (MTT assay)		Paclitaxel (IC_50_ = 1.71 μM)	
		SW982 (MTT assay)		Paclitaxel (IC_50_ = 1.99 μM)	
Pterocephaline	Anti-inflammatory	Inhibition of LPS-induced NO production in RAW264.7 macrophages	No effect	Not reported	[101]
Pterocenoid B	Anti-inflammatory	Inhibition of NO release in RAW264.7 macrophages	IC_50_ = 36.0 μM	Quercetin (IC_50_ = 22.8 μM)	[193]
		Inhibition of the production of TNF-α in in LPS-induced RAW264.7 macrophages	Inhibition %~60% (at the concentration of 50 μM)	Not reported	
		Inhibition of TNF-induced NF-κB activation in a luciferase reporter gene	Not reported	Not reported	[192]
Pterocenoid C	Anti-inflammatory	Inhibition of TNF-induced NF-κB activation in a luciferase reporter gene	Not reported	Not reported	[192]
Pterocenoid E	Anti-inflammatory	Inhibition of NO release in RAW264.7 macrophages	No effect	Quercetin (IC_50_ = 22.8 μM)	[193]
Pterocenoid F	Anti-inflammatory	Inhibition of NO release in RAW264.7 macrophages	No effect	Quercetin (IC_50_ = 22.8 μM)	[193]
Pterocenoid G	Anti-inflammatory	Inhibition of NO release in RAW264.7 macrophages	No effect	Quercetin (IC_50_ = 22.8 μM)	[193]
Pterocenoid H	Anti-inflammatory	Inhibition of NO release in RAW264.7 macrophages	No effect	Quercetin (IC_50_ = 22.8 μM)	[193]
Pteroceside A	Enzymatic	α-glucosidase from *Saccharomyces cerevisiae*	IC_50_ = 38.46 μM	Acarbose (IC_50_ = 175.00 μM)	[34]
Pteroceside C	Enzymatic	α-glucosidase from *Saccharomyces cerevisiae*	IC_50_ = 82.01 μM	Acarbose (IC_50_ = 175.00 μM)	[34]
Pubescensoside	Antitumoral	A459 (MTT assay)	IC_50_ = 13.9 μg/mL	Not reported	[194]
Rapulaside A	Platelet aggregation	Effect after induction by PAF in rabbits	Aggregation % = 42.9%	BN52021 (Aggregation % = 0.6%)	[200]
		Effect after induction by AA in rabbits	Aggregation % = 69.2%	Aspirin (Aggregation % = 4.7%)	
		Effect after induction by ADP in rabbits	Aggregation % = 68.9%	Aspirin (Aggregation % = 65.9%)	
Rapulaside B	Platelet aggregation	Effect after induction by PAF in rabbits	Aggregation % = 53.9%	BN52021 (Aggregation % = 0.6%)	[200]
		Effect after induction by AA in rabbits	Aggregation % = 73.6%	Aspirin (Aggregation % = 4.7%)	
		Effect after induction by ADP in rabbits	Aggregation % = 66.8%	Aspirin (Aggregation % = 65.9%)	
Reticunin A	Anti-inflammatory	Inhibition of NO production in LPS-stimulated RAW264.7 macrophages	No effect	Indomethacin (IC_50_ = 46.71 μg/mL)	[201]
Reticunin B	Anti-inflammatory	Inhibition of NO production in LPS-stimulated RAW264.7 macrophages	No effect	Indomethacin (IC_50_ = 46.71 μg/mL)	[201]
Rotunduside	Antibacterial	Inhibitory activity on MRB (chemiluminescence)	IC_50_ = 198.09 μmol/L	Rutin (IC_50_ = 15.07 μmol/L)	[202]
				Dexamethasone (IC_50_ = 355.14 μmol/L)	
Rotundoside A	Antibacterial	Inhibitory activity on MRB (chemiluminescence)	IC_50_ = 217.13 μmol/L	Rutin (IC_50_ = 15.07 μmol/L)	[203]
				Dexamethasone (IC_50_ = 355.14 μmol/L)	
Saprosmoside E	Anti-inflammatory	Inhibition of NO production in LPS-activated RAW264.7 macrophage cells	No effect	Indomethacin (IC_50_ = 23.93 μM)	[108]
Saprosmoside F	Anti-inflammatory	Inhibition of NO production in LPS-activated RAW264.7 macrophage cells	IC_50_ = 39.57 μM	Indomethacin (IC_50_ = 23.93 μM)	[108]
Saungmaygaoside A	Antiviral	Inhibition of the expression of Vpr in TREx-HeLa-Vpr cells	Cell proliferation % = 79% (at the concentration of 10 μM)	Damnacanthal (Cell proliferation % = 158% at the concentration of 10 μM)	[23]
Saungmaygaoside B	Antiviral	Inhibition of the expression of Vpr in TREx-HeLa-Vpr cells	Cell proliferation % = 105% (at the concentration of 10 μM)		
Saungmaygaoside C	Antiviral	Inhibition of the expression of Vpr in TREx-HeLa-Vpr cells	Cell proliferation % = 120% (at the concentration of 10 μM)		
Saungmaygaoside D	Antiviral	Inhibition of the expression of Vpr in TREx-HeLa-Vpr cells	Cell proliferation % = 144% (at the concentration of 10 μM)		
Sclerochitonoside C	Insecticidal	Mortality of immature *Frankliniella occidentalis*	Mortality % = 15% (at the concentration of 0.10 mM)	Not reported	[208]
Seemannoside A	Antifungal	*Cladosporium cucumcvinum*	No effect	Propiconazole (MIC = 1 μg/mL)	[209]
Seemannoside B	Antifungal	*Cladosporium cucumcvinum*	No effect	Propiconazole (MIC = 1 μg/mL)	[209]
Septemfidoside	Antioxidant	DPPH^·^	No effect	Ascorbic acid (IC_50_ = 6.3 μg/mL)	[12]
	Antibacterial	*Enterococcus faecalis* ATCC1054	MIC = 125 μg/mL	Gentamycin (MIC = 16 μg/mL)	
				Vancomycin (MIC > 64 μg/mL)	
		*Staphylococcus aureus* CIP53.154	MIC = 250 μg/mL	Gentamycin (MIC = 4 μg/mL)	
				Vancomycin (MIC > 64 μg/mL)	
		*Escherichia coli* CIP54.127	MIC = 500 μg/mL	Gentamycin (MIC = 4 μg/mL)	
				Vancomycin (MIC > 16 μg/mL)	
		*Staphylococcus epidermis*	MIC = 250 μg/mL	Gentamycin (MIC = 0.25 μg/mL)	
				Vancomycin (MIC = 4 μg/mL)	
		*Pseudomonas aeruginosa* ATCC9027	MIC = 250 μg/mL	Gentamycin (MIC = 8 μg/mL)	
				Vancomycin (MIC > 64 μg/mL)	
	Antitumoral	HT1080 (MTT assay)	No effect	Not reported	
	Enzymatic	Mushroom anti-tyrosinase	No effect	Kojic acid (IC_50_ = 6.8 μg/mL)	
Sylvestroside I	Antioxidant	DPPH^·^	No effect	Ascorbic acid (IC_50_ = 6.3 μg/mL)	[12]
	Antibacterial	*Enterococcus faecalis* ATCC1054	MIC = 500 μg/mL	Gentamycin (MIC = 16 μg/mL)	
				Vancomycin (MIC > 64 μg/mL)	
		*Staphylococcus aureus* CIP53.154	MIC = 62.5 μg/mL	Gentamycin (MIC = 4 μg/mL)	
				Vancomycin (MIC > 64 μg/mL)	
		*Escherichia coli* CIP54.127	MIC = 62.5 μg/mL	Gentamycin (MIC = 4 μg/mL)	
				Vancomycin (MIC > 16 μg/mL)	
		*Staphylococcus epidermis*	MIC = 125 μg/mL	Gentamycin (MIC = 0.25 μg/mL)	
				Vancomycin (MIC = 4 μg/mL)	
		*Pseudomonas aeruginosa* ATCC9027	MIC = 125 μg/mL	Gentamycin (MIC = 8 μg/mL)	
				Vancomycin (MIC > 64 μg/mL)	
	Antitumoral	HT1080 (MTT assay)	No effect	Not reported	
	Enzymatic	Mushroom anti-tyrosinase	No effect	Kojic acid (IC_50_ = 6.8 μg/mL)	
	Spasmolytic	Inhibitory effects on the electrically-induced contractions in guinea-pig ileum	Inhibition % > 45% (at the concentration of 0.001 M)	Vancomycin (MIC > 64 μg/mL)	[218]
	Anti-inflammatory	Inhibition of NO production in LPS-activated RAW264.7 macrophage cells	IC_50_ > 50 μM	L-NMMA (IC_50_ = 22.6 μM)	[50]
			IC_50_ = 101.42 μM	L-NMMA (IC_50_ = 19.36 μM)	[65]
Sylvestroside III	Spasmolytic	Inhibitory effects on the electrically-induced contractions in guinea-pig ileum	Inhibition % > 40% (at the concentration of 0.001 M)	Vancomycin (MIC > 64 μg/mL)	[218]
Sylvestroside IV	Antitumoral	Caco2 (MTT assay)	IC_50_ = 7.27 μM	Paclitaxel (IC_50_ = 2.63 μM)	[24]
		Huh-7 (MTT assay)	IC_50_ = 11.41 μM	Paclitaxel (IC_50_ = 1.71 μM)	
		SW982 (MTT assay)	IC_50_ = 7.23 μM	Paclitaxel (IC_50_ = 1.99 μM)	
Sylvestroside IV dimethyl acetal	Antiviral	Inhibition of the expression of Vpr in TREx-HeLa-Vpr cells	Cell proliferation % = 171% (at the concentration of 10 μM)	Damnacanthal (Cell proliferation % = 158% at the concentration of 10 μM)	[23]
	Antitumoral	Caco2 (MTT assay)	No effect	Paclitaxel (IC_50_ = 2.63 μM)	[24]
		Huh-7 (MTT assay)	No effect	Paclitaxel (IC_50_ = 1.71 μM)	
		SW982 (MTT assay)	No effect	Paclitaxel (IC_50_ = 1.99 μM)	
Swerilactone A	Antiviral	HBV virus (inhibition of the secretion of HBsAg in HepG 2.2.15 cells)	IC_50_ = 3.66 mM	Not reported	[215]
		HBV virus (inhibition of the secretion of HBeAg in HepG 2.2.15 cells)	IC_50_ = 3.58 mM		
Swerilactone B	Antiviral	HBV virus (inhibition of the secretion of HBsAg in HepG 2.2.15 cells)	No effect	Not reported	[215]
		HBV virus (inhibition of the secretion of HBeAg in HepG 2.2.15 cells)			
Swertianoside A	Antiviral	Hepatitis B virus effects (inhibition on the secretion of HBsAg)	IC_50_ = 0.18 mM	Tenofovir (IC_50_ = 1.31 mM)	[217]
		Hepatitis B virus effects (inhibition on the secretion of HBeAg)	IC_50_ = 0.12 mM	Tenofovir (IC_50_ = 1.15 mM)	[217]
Triplostoside A	Anti-inflammatory	Inhibition of NO production in LPS-activated RAW264.7 macrophage cells	No effect	Not reported	[106]
			No effect	L-NMMA (IC_50_ = 19.36 μM)	[65]
			IC_50_ > 50 μM	L-NMMA (IC_50_ = 22.6 μM)	[50]
	Antitumoral	A549 (MTT assay)	No effect	Florouracil (IC_50_ = 0.177 μg/mL)	[48]
		Bel7402 (MTT assay)		Florouracil (IC_50_ = 0.542 μg/mL)	
		BGC-823 (MTT assay)		Florouracil (IC_50_ = 0.695 μg/mL)	
		HCT-8 (MTT assay)		Florouracil (IC_50_ = 0.67 μg/mL)	
		A2780 (MTT assay)		Florouracil (IC_50_ = 0.569 μg/mL)	
Valeridoid B	Antitumoral	GSC-3 (MTT assay)	No effect	Not reported	[233]
		GSC-12 (MTT assay)			
		GSC-18 (MTT assay)			
Valeridoid C	Antitumoral	GSC-3 (MTT assay)	No effect	Not reported	[233]
		GSC-12 (MTT assay)			
		GSC-18 (MTT assay)			
Valeridoid D	Antitumoral	GSC-3 (MTT assay)	No effect	Not reported	[233]
		GSC-12 (MTT assay)			
		GSC-18 (MTT assay)			
Valeridoid E	Antitumoral	GSC-3 (MTT assay)	No effect	Not reported	[233]
		GSC-12 (MTT assay)			
		GSC-18 (MTT assay)			
Valeridoid F	Antitumoral	GSC-3 (MTT assay)	IC_50_ = 42.42 μM	Not reported	[233]
		GSC-12 (MTT assay)	IC_50_ = 41.4 μM		
		GSC-18 (MTT assay)	IC_50_ = 47.55 μM		

Legend: DIZ = diameter of inhibition zone; EC_50_ = half-maximal effective response; IC_50_ = half-maximal inhibitory concentration; LC_50_ = half-maximal lethal concentration; MIC = minimum inhibitory concentration.
